# BertSNR: an interpretable deep learning framework for single-nucleotide resolution identification of transcription factor binding sites based on DNA language model

**DOI:** 10.1093/bioinformatics/btae461

**Published:** 2024-08-06

**Authors:** Hanyu Luo, Li Tang, Min Zeng, Rui Yin, Pingjian Ding, Lingyun Luo, Min Li

**Affiliations:** School of Computer Science and Engineering, Central South University, Changsha, Hunan 410083, China; School of Computer Science, University of South China, Hengyang, Hunan 421001, China; School of Computer Science and Engineering, Central South University, Changsha, Hunan 410083, China; School of Computer Science and Engineering, Central South University, Changsha, Hunan 410083, China; Department of Health Outcome and Biomedical Informatics, University of Florida, Gainesville, FL 32611, United States; Center for Artificial Intelligence in Drug Discovery, School of Medicine, Case Western Reserve University, Cleveland, OH 44106, United States; School of Computer Science, University of South China, Hengyang, Hunan 421001, China; School of Computer Science and Engineering, Central South University, Changsha, Hunan 410083, China

## Abstract

**Motivation:**

Transcription factors are pivotal in the regulation of gene expression, and accurate identification of transcription factor binding sites (TFBSs) at high resolution is crucial for understanding the mechanisms underlying gene regulation. The task of identifying TFBSs from DNA sequences is a significant challenge in the field of computational biology today. To address this challenge, a variety of computational approaches have been developed. However, these methods face limitations in their ability to achieve high-resolution identification and often lack interpretability.

**Results:**

We propose BertSNR, an interpretable deep learning framework for identifying TFBSs at single-nucleotide resolution. BertSNR integrates sequence-level and token-level information by multi-task learning based on pre-trained DNA language models. Benchmarking comparisons show that our BertSNR outperforms the existing state-of-the-art methods in TFBS predictions. Importantly, we enhanced the interpretability of the model through attentional weight visualization and motif analysis, and discovered the subtle relationship between attention weight and motif. Moreover, BertSNR effectively identifies TFBSs in promoter regions, facilitating the study of intricate gene regulation.

**Availability and implementation:**

The BertSNR source code can be found at https://github.com/lhy0322/BertSNR.

## Graphical Abstract

**Graphical Abstract:**
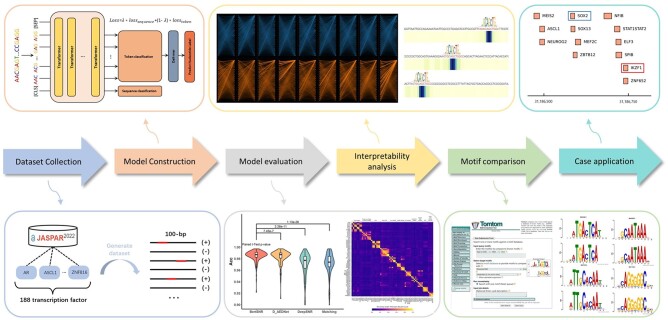

## 1 Introduction

Transcription factors (TFs) are a class of protein molecules that play a pivotal role for the regulation of gene transcription. They are crucial in the intricate process of gene expression by recognizing and binding to specific segments within DNA, thereby modulating the transcription of particular genes (Wasserman and Sandelin 2004, Lambert *et al.* 2018). These specific DNA segments, recognized as transcription factor binding sites (TFBSs), are frequently situated on enhancers or promoters (Stormo 2000, Andersson and Sandelin 2020). Enhancers and promoters serve as crucial regulatory elements in gene expression, typically found in the upstream region of genes (Li *et al.* 2022). They exert control over gene transcription levels by interacting with TFs (Shlyueva *et al.* 2014, Tippens *et al.* 2018). It is noteworthy that the nucleic acid sequence to which a TF binds resides within non-coding regions, and it cannot be directly translated into protein. Nevertheless, it possesses the capability to regulate the expression of genes located downstream of its binding site. It is recognized that the onset and progression of certain diseases can be attributed to the aberrant expression or dysfunction of TFs (Weirauch *et al.* 2014, Gomez-Pastor *et al.* 2018, Tiwari and Pal 2022). Therefore, the identification of TF binding regions and their impact on genes not only enhances our understanding of cellular gene expression regulation mechanisms but also provides a critical theoretical and practical foundation for disease prevention and treatment strategies (Bulyk 2003, Chai *et al.* 2016, Cuadrado *et al.* 2018).

Chromatin Immunoprecipitation followed by Sequencing (ChIP-seq) (Furey 2012) is a powerful molecular biology technique used to investigate protein-DNA interactions and identify the binding sites of specific proteins within the genome. ChIP-seq data have yielded a substantial corpus of information regarding TFBSs (Hu *et al.* 2010, Yevshin *et al.* 2016). This extensive dataset presents a unique opportunity for the development of predictive models aimed at identifying TF motifs and pinpointing TFBSs (Bailey *et al.* 2006). Leveraging this abundant biological sequence data available, deep learning techniques have been widely applied to TFBSs modeling (He *et al.* 2021). For example, DeepBind (Alipanahi *et al.* 2015) was one of the pioneering methods to employ convolutional neural networks (CNNs) for DNA sequence modeling, surpassing the performance of traditional computational approaches. Nevertheless, CNN-based methods ignore positional information between nucleotides due to the convolutional kernel that only focus on the local features. To address the limitation, DeepTF (Bao *et al.* 2019) was proposed, employing long short-term memory (LSTM) networks for feature integration following CNN-based feature extraction. This approach significantly enhances the accuracy of TFBS prediction. Subsequently, more methods based on deep learning to predict TFBSs have been proposed, each surpassing its predecessor in performance (Shen *et al.* 2021, Zhang *et al.* 2022, Luo *et al.* 2023). However, these methods are primarily designed to determine the presence or absence of TFBSs within a sequence, lacking the ability to accurate pinpoint the location of TFBSs at single-nucleotide resolution. This challenge remains a significant issue in the field of TFBS prediction (Shi *et al.* 2023).

DeepSNR (Salekin *et al.* 2018) is the first deep learning model that accomplishes the single-nucleotide resolution prediction of TFBSs, using a combination of convolutional and deconvolutional neural networks. It is inspired by the similarity between TFBS localization tasks and image segmentation tasks. Just as image segmentation involves categorizing each pixel as belonging to either a target object or background, DeepSNR performs one-hot encoding on DNA sequences and uses an image segmentation approach to classify individual nucleotides as either constituting a binding site or representing background sequence. This innovative approach empowers the accurate prediction of TFBSs at the nucleotide-level. Subsequently, D-AEDNet (Zhang *et al.* 2021) emerged as an advancement upon DeepSNR, introducing an encoder–decoder architecture for to further improve TFBS prediction. D-AEDNet incorporates several sophisticated deep learning components, including the utilization of the attention mechanism with residual concatenation. However, both DeepSNR and D-AEDNet rely on one-hot encoding and CNN networks, which inherently possess limitations in capturing and responding to contextual semantic information embedded within sequences. This limitation arises from the fact that the ability of CNN to extract localized features is limited by filter size.

**Figure 1.**
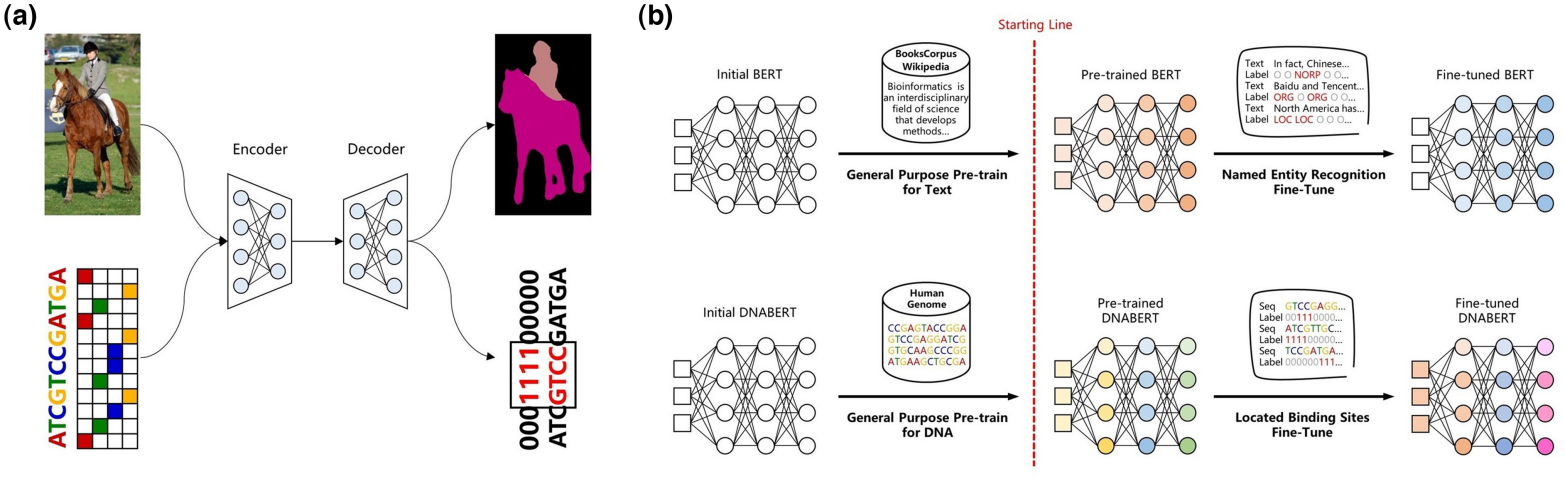
Single-nucleotide resolution TFBS prediction models draw inspiration from other tasks. (a) Drawing inspiration from the image segmentation task, the sequence is treated as a one-dimensional four-channel image, which undergoes segmentation through the encoder and decoder. (b) Drawing inspiration from LLMs and the principles of named entity recognition, sequences were conceptualized as DNA languages, subsequently undergoing fine-tuning via pre-trained language models to identify binding sites

In recent years, the advent of large language models (LLMs), e.g. GPT-3 (Brown *et al.* 2020) and BERT (Devlin *et al.* 2018), has revolutionized the field of natural language processing (NLP) (Wen *et al.* 2023). These models have demonstrated unparalleled performance across a broad range of NLP tasks (Chang *et al.* 2023). They undergo training on extensive textual datasets to acquire comprehensive knowledge the syntactic, semantic, and contextual aspects of natural language. Subsequently, they are applied to various downstream tasks. In the field of bioinformatics, several LLMs have emerged (Zhang *et al.* 2023). DNABERT (Ji *et al.* 2021), a DNA-specific language model, serves as a notable example. DNABERT was pre-trained on the entire human reference genome, achieving state-of-the-art performance in downstream tasks, including promoter prediction. Inspired by these breakthroughs, we propose a novel model for predicting TFBSs. Unlike previous methods (Salekin *et al.* 2018, Zhang *et al.* 2021) that treat DNA sequences as images for segmentation (Fig. 1a), our model considers sequences as DNA languages. As illustrated in Fig. 1b, the task of single-nucleotide resolution TFBS prediction finds an analogy with named entity recognition (NER) task in natural language processing (Li *et al.* 2020). This paradigm shift allows us to explore TFBS prediction from innovative insights and methodologies.

In the study, we introduce BertSNR, a novel and interpretable deep learning framework, which leverages a DNA language model for the accurate identification of TFBSs at single-nucleotide resolution. We applied our model to a comprehensive collection of 188 distinct TF datasets for TFBS prediction. Through meticulous evaluation, our proposed model, BertSNR, exhibited superior performance compared to state-of-the-art methods across all datasets, demonstrating its efficacy in TFBS prediction. Furthermore, we conducted a thorough analysis of the model’s attention weights, providing clear insights into its decision-making process and enhancing overall interpretability. The motifs generated by BertSNR were found to closely align with reference motifs in the database, underscoring the model’s ability to capture meaningful sequence patterns. Additionally, we successfully identified TFBSs in the POU5F1 promoter region, corroborating previous research findings and uncovering numerous potentially unexplored TFBSs. These results collectively highlight the effectiveness and interpretability of BertSNR in the accurate prediction and analysis of TFBSs.

## 2 Materials and methods

### 2.1 Data collection and processing

To develop an effective and generalized model, it is imperative to collect a comprehensive TFBS datasets. JASPAR (Castro-Mondragon *et al.* 2022) is a widely employed repository in the fields of bioinformatics and molecular biology, renowned for its source of high-caliber information pertaining to TFBSs. In our study, we first scrutinized the TFBSs specific to *Homo sapiens* within the JASPAR database, focusing on data obtained from ChIP-seq experiments. Subsequently, we excluded datasets without locus information, and we finally obtained a total of 188 TFBS datasets, complemented by an additional 33 datasets for a motif analysis (Supplementary Table S1).

To construct the datasets for TFBS prediction, for each of the 188 TFBSs datasets, we systematically extracted a 100-bp sequence encompassing each binding site, with the binding site randomly positioned within this sequence. This approach ensures that the TFBS is included within the sequence while allowing for variability in the surrounding sequence context. Subsequently, corresponding labels were generated at the nucleotide-level, where “1” indicated nucleotides residing within the binding site, and “0” represented those outside it. Additionally, we designated sequences that contained binding sites as positive sample sequences. Negative sample sequences were generated by shuffling the positive sample sequences while preserving dinucleotide frequencies (Jiang *et al.* 2008). In these negative sample sequences, all nucleotide labels were uniformly assigned a value of “0”. Consequently, this approach maintained a balanced ratio of positive to negative samples at the sequence level, resulting in a 1:1 ratio. However, at the nucleotide-level, the ratio of positive to negative samples approximated 1:15. Supplementary Figure S1 details the process of generating the dataset. Furthermore, for each TFBS dataset, we partitioned 80% of the data for training and the remaining 20% for testing. Detailed information pertaining to the dataset can be found in Supplementary Table S2.

### 2.2 The framework of BertSNR

Here, we leverage DNABERT (Ji *et al.* 2021) pre-trained on the human reference genome to construct the framework of our model. DNABERT’s primary objective is to acquire embeddings and representations for DNA sequences. These generated embeddings enable the model to capture and assimilate the contextual and biological information inherent in DNA sequences, which can be applied to a diverse spectrum of tasks within the domains of bioinformatics and computational biology.

**Figure 2.**
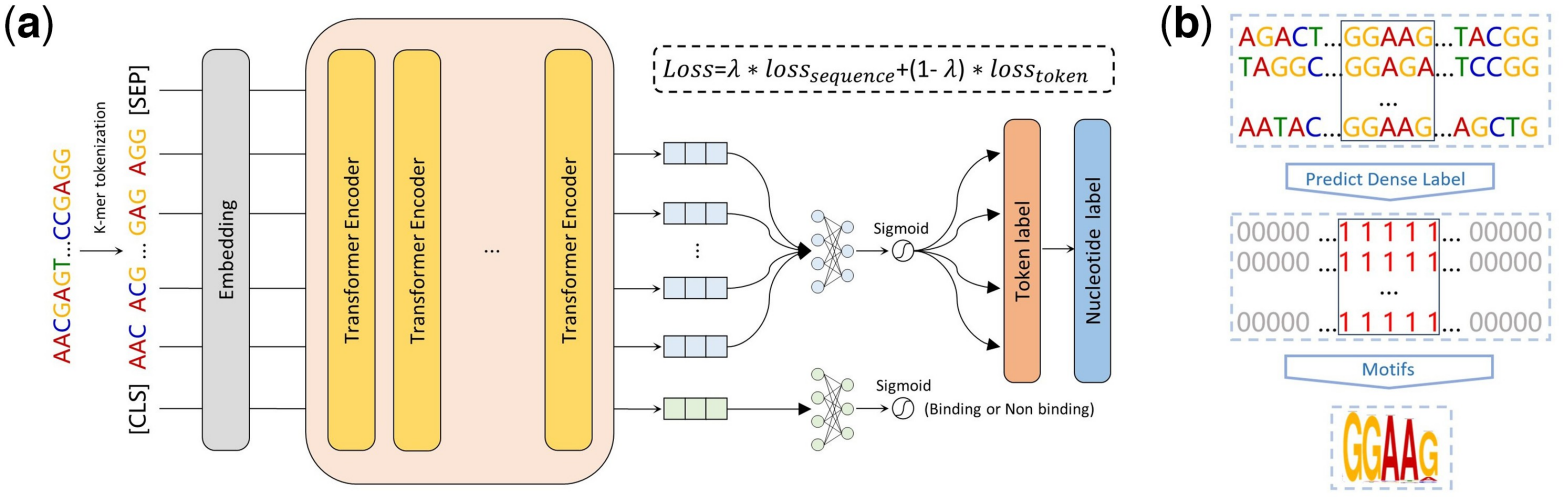
The architecture of BertSNR and the example of motif generation. (a) Upon inputting the DNA sequence into BertSNR, the initial operation involves k-mer tokenization. Following this, embedding vectors are generated for each token, and these vectors undergo feature extraction through a multi-layer Transformer. Subsequently, multi-task learning is employed to generate token labels, which are further transformed into nucleotide labels. (b) For each DNA sample, the nucleotides in the matrix were TFBSs, while the other nucleotides were non-TFBSs. All TFBSs underwent alignment, and motifs were subsequently generated based on the nucleotide frequencies at their respective positions

Based on DNABERT, we propose a novel and interpretable deep learning model BertSNR (**B**idirectional **e**ncoder **r**epresentation from **t**ransformers for **S**ingle-**N**ucleotide **R**esolution). BertSNR is designed with the aim of accurate identifying TFBSs at single-nucleotide resolution. It comprises four components: k-mer tokenization, embedding, feature extraction, and prediction (Fig. 2a and Supplementary Fig. S2).

#### 2.2.1 K-mer tokenization

Rather than treating individual nucleotides as single tokens, our DNA language model adopts a strategy of tokenization based on k-mers. Here, a k-mer refers to a contiguous substring comprising *k* consecutive nucleotides, a widely employed approach in the analysis of biological sequences (Hong *et al.* 2020). For instance, considering the DNA sequence “ATCGAT,” tokenization using 3-mers yields {ATC, TCG, CGA, GAT}, while 4-mers result in {ATCG, TCGA, CGAT}. We stipulate the value of *k* as a hyperparameter, ranging from 3 to 6, aligning with the pre-trained model of DNABERT. The vocabulary is composed of all possible permutations of k-mers encompassing nucleotides with five special tokens ([CLS], [PAD], [UNK], [SEP], and [MASK]), resulting in a total of $4^{k}$+5 tokens (69 tokens for *k* = 3)*.* To ensure consistent interchangeability between nucleotide labels and k-mer token labels, we have established a specific criterion. In this framework, the label for the *k* nucleotides, which are translated into the corresponding k-mer tokens, assumes a value of “1” solely when all *k* nucleotides carry a “1” label. Similarly, in cases where k-mer tokens bear a label of “1”, the corresponding *k* nucleotides are uniformly assigned a “1” label, while the labels for the remaining nucleotides are designated as “0”. This comprehensive schema is visually elucidated in Supplementary Fig. S3.

#### 2.2.2 Embedding

Following the tokenization of the DNA sequences, we generate two distinct categories of embeddings for each token: token embeddings and position embeddings. Token embeddings (Ng 2017) represent as the outcome of the conversion of each token into dense vector representations, which have been pre-trained employing the human genome as the reference. Within the model, every token is associated with a unique embedding vector. Position embeddings (Devlin *et al.* 2018), on the other hand, serve the purpose of encapsulating details regarding the relative positions of individual tokens within the sequence. This is realized by assigning each token a vector that signifies its specific position within the sequence. The summation of these two distinct categories of embeddings constitutes the composite input embeddings for the DNA language model. This comprehensive framework empowers the model to proficiently encapsulate both token-specific information and positional context, as explicitly defined below:

$$E_{i}^{\mathrm{input}}=E_{i}^{\mathrm{token}}+E_{i}^{\mathrm{position}}$$

where $E_{i}^{\mathrm{input}},\;E_{i}^{\mathrm{token}},\;\mathrm{and}\;E_{i}^{\mathrm{position}}$ represent the ultimate input embedding, token embedding, and position embedding of the *i*-th token, respectively.

#### 2.2.3 Feature extraction

We adopt a multilayer bi-directional Transformer encoder architecture (Vaswani *et al.* 2017). Within this Transformer encoder, two primary sub-layers are incorporated, namely, a multi-head self-attention layer and a feed-forward fully connected layer. Each of these sub-layers is endowed with residual networks and layer normalization with an output of $\mathrm{LayerNorm}\left(x+\mathrm{Sublayer}(x)\right)$. At the heart of the Transformer encoder lies the multi-head self-attention mechanism, a pivotal element that empowers the model to capture intricate relationships among all tokens within the contextual window. This, in turn, leads to the generation of a highly informative encoded vectors, facilitating nuanced contextual comprehension from diverse vantage points. The stepwise computational process for executing the multi-head self-attention mechanism on input data X unfolds as follows:

$$\mathrm{MultiHeadAtt}\left(X\right)=\mathrm{Concat}({\mathrm{head}}_{1},\;\ldots ,{\mathrm{head}}_{n})W^{O}$$

$${\mathrm{head}}_{i}=\mathrm{softmax}\left(\frac{XW_{i}^{Q}\cdot {\left(XW_{i}^{K}\right)}^{T}}{\sqrt{d_{k}}}\right){\cdot XW}_{i}^{V}$$

where $W^{O}$ and $\{W_{i}^{Q},\;W_{i}^{K},\;W_{i}^{V}\;{\}}_{i=0}^{n}$ are learned parameters for the linear projection of the ith attention head, and n denotes the total number of attentional heads. $d_{k}$ represents the dimension of each attention head. $\sqrt{d_{k}}$ denotes the feature scaling factor, which serves to adjust the size of the attention scores in order to improve the training stability of the model.

#### 2.2.4 Prediction

Multi-task learning model is an effective approach for enhancing the performance of individual tasks by leveraging knowledge from other related tasks (Ruder 2017). Typically, such models are structured into shared layers and private layers. The shared layer is responsible for extracting features that are commonly relevant to multiple tasks, while the private layer extracts task-specific features (Zhou *et al.* 2019). Ji *et al.* (2021) have demonstrated the efficacy of DNA language model in capturing inherent patterns within sequence-level binary classification tasks. Building upon this insight, we define two tasks for our TFBS prediction at the nucleotide-level: sequence-level classification (determining the presence or absence of binding sites) and token-level classification (identifying the accurate localization of binding sites). Supplementary Figure S4 provides a detailed comparison between the sequence-level and token-level classification tasks.

In our framework, a 12-layer transformer is used as the shared layer to extract features followed by dedicated private layers for each of the two tasks. These private layers consist of fully connected layers with sigmoid activation functions. The loss function for multi-task learning is defined as follows:

$$\mathrm{Loss}=\lambda *{\mathrm{loss}}_{\mathrm{sequence}}+\left(1-\lambda \right)\;*\;{\mathrm{loss}}_{\mathrm{token}}$$

where λ is a hyperparameter used to balance the weights during training of the two tasks. ${\mathrm{loss}}_{\mathrm{sequence}}$ and ${\mathrm{loss}}_{\mathrm{token}}$ are both binary cross-entropy loss functions. Subsequently, the resulting token labels are mapped to nucleotide labels, yielding accurate positions of the TFBSs (Supplementary Fig. S3).

### 2.3 Hyperparameters selection and model implementation

We trained separate models for each TF to achieve identification and prediction of TFBSs specific to each TF. In the process of model training, a five-fold cross-validation approach was employed to systematically explore and optimize hyperparameters using the training dataset. Subsequently, we assessed model performance on the testing dataset. This methodology ensures the independence of the test dataset for unbiased evaluation. We set the number of epochs to 10 and employed an early stopping method to train the optimal model. For detailed information on model training and hyperparameters, please refer to Supplementary Table S3. Both our proposed BertSNR and the comparative methods were implemented utilizing the PyTorch framework. All experiments were conducted on an NVIDIA TITAN X graphics processing unit (GPU).

### 2.4 Motif analyses

To visualize the identification of TF binding motifs, according to the model’s prediction of binding sites, we applied the motif discovery algorithm introduced by Zhang *et al.* (2021), as illustrated in Fig. 2b. The motif discovery algorithm represents a post-processing technique designed to identify subsequences exhibiting specific patterns and biological significance within the input sequences, leveraging the model’s predictions. The process begins by initializing a base sequence window, which is then scanned in the window to determine the presence of binding sites based on a confidence threshold. Subsequently, the central region of each consecutive window containing binding sites is designated as a collection of sequence primitives.

## 3 Results

### 3.1 BertSNR accurately identifies TFBSs at single-nucleotide resolution

To conduct a comprehensive evaluation of our model’s performance, we conducted an exhaustive analysis by comparing BertSNR with three existing cutting-edge nucleotide-level TFBS prediction methods: Matching (Kel *et al.* 2003), DeepSNR (Salekin *et al.* 2018), and D-AEDNet (Zhang *et al.* 2021). Supplementary methods provide illustrations of the three methods for reference. We tested these models across 188 TFBS datasets to obtained from multiple sources. To gauge the effectiveness of the models, we employed six widely recognized evaluation metrics: accuracy, precision, recall, F1-score, area under the receiver operating characteristic curve (AUC), and area under the precision–recall curve (AUPR). Detailed explanations of these metrics and their calculation methods can be found in the Supplementary methods. It is critical to note that all models were trained on the same training dataset, ensuring a fair comparison. It is worth highlighting limitation of the Matching method, which is that it can only identify whether a site is a TFBS or not, without providing probability value. Thus, we were unable to compute AUC and AUPR values for the Matching method.

**Figure 3.**
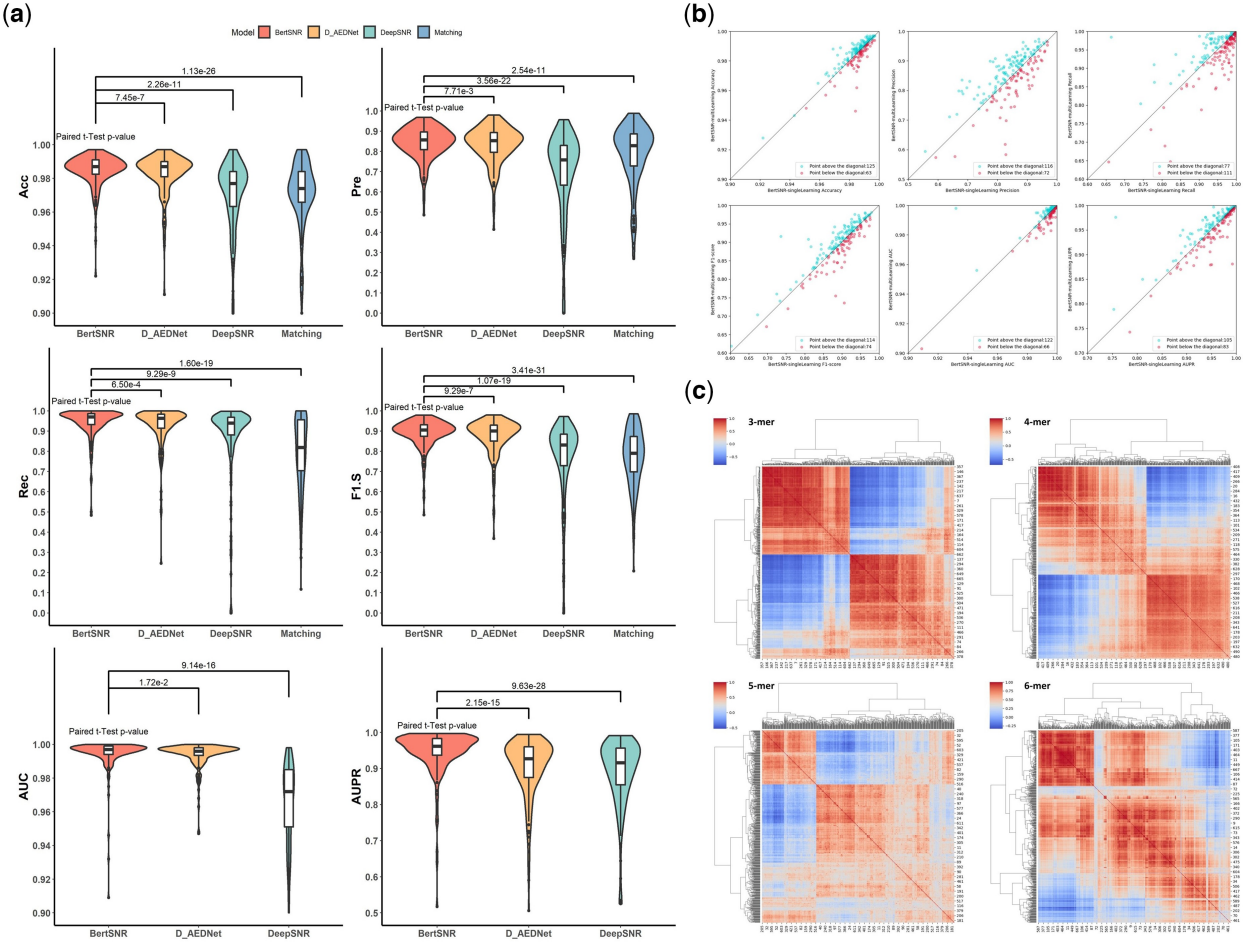
BertSNR improves TFBS prediction through multi-task learning and 3-mer DNA language modeling. (a) Violin plots illustrate the metrics of accuracy, precision, recall, F1 score, AUC, and AUPR for single-nucleotide resolution TFBS predictions with the 188 TFs datasets. Paired t-tests were employed to perform two-by-two comparisons between these metrics. (b), Head-to-head accuracy, precision, recall, F1 score, AUC and AUPR comparison of BertSNR-multi and BertSNR-single on 188 TFs datasets. (c) Heatmap comparing the degree of correlation of sequence representations generated by different k-mer modeling using the Pearson correlation coefficient

To provide a comprehensive comparison of BertSNR with the other three methods for the task of single-nucleotide resolution TFBS prediction, we visually presented the results using a violin plot in Fig. 3a. Additionally, detailed comparative data for each dataset can be found in meticulously documented Supplementary Tables S4–S7. It is noteworthy that BertSNR exhibits outstanding performance, as evidenced by the distribution of AUPR values across the 188 datasets, which surpasses that of the second-best performing method (D-AEDNet) based on a Paired Student *t*-test with a *P*-value of 2.15e−15. Additionally, BertSNR consistently outperforms existing methods across the remaining five evaluation metrics.

We also conducted a comparison of the models on the sequence-level prediction task, which involves determining the presence or absence of TFBSs within entire sequences. For this task, our results demonstrate that BertSNR consistently outperforms the other methods. Detailed performance comparisons for the sequence-level prediction task are illustrated in Supplementary Fig. S5. This consistent superiority further emphasizes the capabilities of BertSNR, which is built upon the DNA language model, to decipher implicit sequence representations from a novel perspective, a capability not readily attainable with CNN-based models.

### 3.2 Multi-task learning can effectively improve TFBS identification

To evaluate the impact of multi-task learning on the prediction of TFBS identification, we conducted a comparative study. In this study, we introduced a variant architecture called BertSNR-single by removing the sequence-level prediction task from the original model, BertSNR-multi. The same training and testing procedures were applied to both models across the 188 datasets. The results of this investigation are presented in Supplementary Table S8. The analysis reveals that BertSNR-multi exhibits enhancements in five out of six evaluation metrics. Specifically, we could observe slight improvements in accuracy (0.1%), precision (0.8%), F1-score (0.2%), AUC (0.1%), AUPR (0.3%) on average.

To visually represent the comparative analysis between multi-task learning and single-task learning, we present Fig. 3b, a scatterplot illustrating the metric values achieved by each respective method. In this scatterplot, blue points are positioned above the diagonal line, while red points are situated below it. Notably, a significant proportion of points are located above the diagonal, indicating that multi-task learning effectively harnesses knowledge from complementary tasks, leading to a modest enhancement in the model’s performance. These findings underscore the advantages of multi-task learning in bolstering the model’s predictive capabilities for TFBS identification.

### 3.3 Utilization of 3-mer modeling results in better DNA language representation

DNABERT was pre-trained on the human genome using different k-mer values (*k* = 3, 4, 5, 6) for modeling. To assess the impact of different k-mer settings, we conducted a comparative analysis of these pre-trained models that employed various k-mer settings. Specifically, during the preprocessing stage, we employed different k-mer values to generate tokens, and subsequently trained and tested on each of the 188 datasets. The experimental results are presented in Supplementary Table S9. Our findings indicate that the performance of the four k-mer modeling methods is remarkably similar, with a slight advantage observed for the 3-mer modeling method. Notably, the 3-mer modeling method shows a 0.4% improvement in the average AUPR metric compared to the second-best method (4-mer). Moreover, it exhibits reduced standard deviation, indicating increased stability in its performance.

To further analyze the advantages and disadvantages of different k-mer modeling methods, we selected the same number of positive sample sequences (containing TFBSs) and negative sample sequences (not containing TFBSs) into the four different k-mer models. We then took out the header vectors [CLS] (Devlin *et al.* 2018) that represent the entire sequence as output by the last layer of Transformer. Among these representation vectors [CLS], we calculated the Pearson correlation coefficients between each pair of vectors in different k-mer and used this to cluster the representation vectors to obtain the corresponding correlation heatmaps. Figure 3c shows the correlation heatmaps for different k-mer modeling methods. From the correlation heatmaps, it becomes evident that the 3-mer modeling method exhibits a notably superior clustering effect and a higher degree of correlation compared to the other methods. This analysis provides additional support for the assertion that the use of 3-mer modeling results in enhanced sequence representation in terms of both performance and correlation.

### 3.4 BertSNR can predict different TFBSs of the same family

TFs belonging to the same family often exhibit similar binding site motifs. This phenomenon arises from the shared DNA binding preferences among TFs within the same family, coupled with the retention of structural domains and amino acid sequences with evolutionary significance. Consequently, these similarities lead to the identification and binding of similar nucleic acid sequences on DNA. In light of this characteristic, our objective was to investigate whether a model trained on one TFBS dataset could contribute to identifying binding sites for other TFs.

To achieve this, we curated 11 TF families from our pool of 188 datasets, ensuring that each family comprising at least four TFs. This selection process yielded a total of 63 TF datasets for cross-TF prediction. More specifically, we evaluated the models trained on individual TFBS datasets by testing them on the test sets of all 63 TFBSs. The heatmap presented in Supplementary Fig. S6a illustrates the AUC and AUPR scores of BertSNR for cross-testing across these 63 datasets. Our observations indicate that the model exhibits robust generalization and adaptability within the same TF family. For example, the TFBSs within the E2F family exhibit a conserved sequence pattern as shown in Supplementary Fig. S6b. BertSNR effectively captures this sequence pattern, enabling the identification of other TFBSs within the E2F family regardless of which TFBS dataset in the E2F family is used for training. However, when applied to different TF families, the model’s performance significantly deteriorates, consistent with the inherent variability among these families. Notably, we found several exceptions to this trend. Such as the case of MYOG from the MyoD/ASC-related family and BHLHE22 from the Tal-related family, where the model’s performance remained unaffected during cross-prediction. This phenomenon can be attributed to the remarkable similarity between the two types of TFBS, as demonstrated in Supplementary Fig. S6c. In conclusion, BertSNR demonstrates a proficient capability for predicting TFBSs within the same TF family.

### 3.5 Interpretable insights from attention mechanisms in BertSNR

In the context of the DNA language model’s attention mechanism, the model computes weight values for each input position, allowing it to focus on particular segments of the input data and potentially shed light on its decision-making process. To tackle the inherent black-box nature of deep learning models, we visualized these attention weights to show their influence on the model's predictions. Furthermore, we conducted a thorough analysis to elucidate the relationship between attention patterns and sequence motifs.

**Figure 4.**
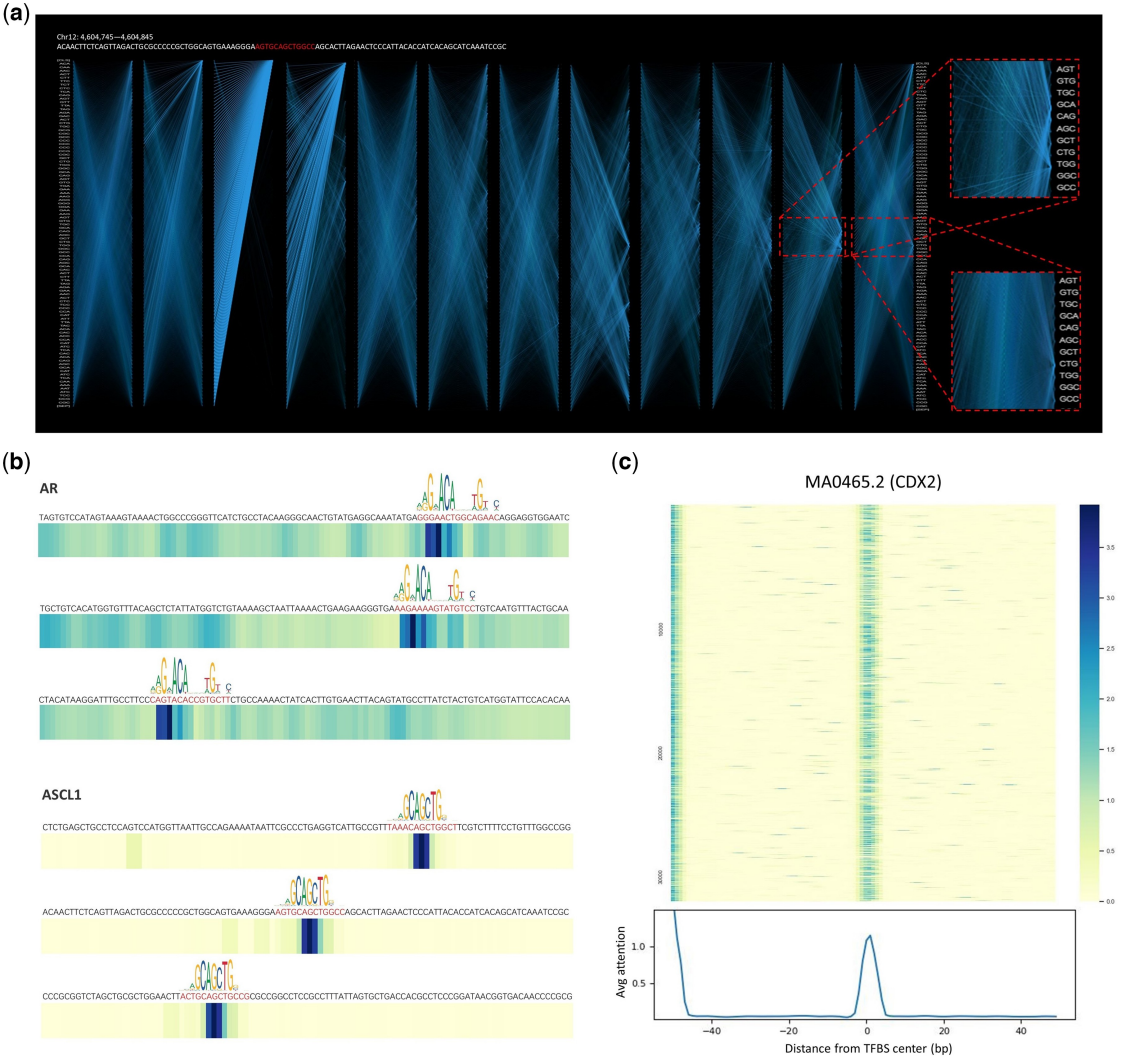
Visualization of attentional weights reveals the interpretability of BertSNR. (a) Visualization of the self-attention mechanism of the 12-layer Transformer for a sample (chr12:4,604,745–4,604,845) from the ASCL1 dataset. (b) The relationship between attention weights of the last layer Transformer and motif logo for three randomly selected samples with different binding sites. (c) Attentional weight visualization of the entire CDX2 dataset and weight averages at each position

To conduct a comprehensive analysis of the self-attention mechanism within BertSNR, we adopted a random sample from the ASCL1 TFBS dataset. Notably, this sample possessed a binding site located in the sequence’s central region and was highlighted in red. Figure 4a showcases the self-attention interaction map. An intriguing observation emerged from this examination. In the early layers of the Transformer, the attention distribution appeared more dispersed and less effective in capturing relevant information. In some cases, it even appears that all attention is focused on the head vector [CLS]. However, as we progressed through the layers, transitioning from shallow to deep representations, a discernible trend emerged. The attention mechanism gradually converged toward the specific token housing the binding site. In the penultimate and ultimate Transformer layers, attention became predominantly fixated on the critical tokens (“CAG” and “CTG”) constituting the binding site. This insightful finding underscores the capacity of the deep Transformer to better discern and harness the implicit information embedded within DNA sequences.

Given the remarkable capability of the deep attention mechanism in capturing meaningful information, we conducted a meticulous analysis of the attention weights within the last layer of the Transformer. Our investigation began by randomly selecting three distinct samples from two TF datasets (AR and ASCL1). Subsequently, we deployed the pre-trained BertSNR model to process these selected samples, meticulously recording the attention weights generated by the last Transformer layer. These recorded attention weights were then subjected to visualization and comparison with the respective motifs present in the database. Figure 4b shows the relationship between attention weight maps and motif enrichment for different samples of both AR and ASCL1 TFs, where darker colors indicate higher attention weights and vice versa. Our observations revealed intriguing insights. For instance, in the case of AR, where not all binding sites are conserved, the model’s attention predominantly converges on the conserved regions within the binding sites. Conversely, regions lacking conservation exhibit relatively lower attention. This phenomenon suggests that the model’s attention may, at times, be dispersed to other regions within the sequence, potentially affecting the model’s decision-making process. In stark contrast, the analysis of ASCL1, characterized by a centrally conserved binding site, unveils a distinct pattern. Here, the model’s attention is notably concentrated and exhibits a strong trend toward the accurate location of the binding site. This compelling finding underscores a strong correlation between the attention mechanism and the conservativeness of binding sites.

To further explore whether BertSNR can discover important patterns in sequences, we visualized an attention map of an entire TF dataset. Specifically, we fixed the binding site of the CDX2 dataset in the center of the sequences and extended it up and down to a sequence length of 100 bp, yielding a total of 32 021 sequences. Subsequently, these sequences were input into the BertSNR model to obtain the attention map. As shown in Fig. 4c, we aggregated the attention maps of all the sequences and calculated their average attention weights for each position. We found that the attention was focused on the center of the sequences, with few distractions. This is consistent with the regions where the TFBSs are typically located. Notably, we found unusually high attention values in the head region of the sequence, presumably due to the effect of the model bias (Ji *et al.* 2021).

Overall, these results significantly enhance our understanding of the model’s inner workings and its applicability in the field of bioinformatics.

### 3.6 BertSNR better matches reference motifs in the database

The motif logo is a graphical representation commonly used to visualize the motif of a TFBSs (Schneider and Stephens 1990), usually consisting of columns of letters, with each column representing the position of a nucleotide. The height of the letters indicates the frequency of nucleotides at that position. In this section, we first identify nucleotide-level labels with three deep learning models, BertSNR, D-AEDNet, and DeepSNR. Subsequently, motif mining is performed on the above labels using the motif discovery algorithm. Next, the mined motifs are used to generate position weight matrix (PWM) (Stormo 2000). Finally, motif comparison is performed using the TOMTOM tool (Bailey *et al.* 2009). Experiments were performed on 33 additional datasets (Supplementary Table S1). The evaluation metrics were *P*-value, *e*-value and *q*-value (Supplementary methods).

Supplementary Tables S10 and S11 provides a visual representation of BertSNR’s performance relative to the other two methods regarding motif generation for TFBSs. Our observations reveal that the motif logos generated by BertSNR closely align with the reference motif logos provided in the database. Conversely, D-AEDNet and DeepSNR exhibit greater dissimilarity from the reference motif logos, sometimes even failing to identify motif-enriched regions in certain datasets. Additionally, BertSNR exhibits superior performance across all three metrics—*P*-value, *e*-value, and *q*-value—when compared to the other two methods. This observation underscores BertSNR’s capacity to harness contextual information and detect underlying patterns within sequences more effectively than CNN-based models.

### 3.7 BertSNR can identify TFBSs in promoter regions

POU5F1 (Oct4), the principal TF in embryonic stem cells (ESC), plays a pivotal role in conferring these cells with the capacity for self-renewal and differentiation into a wide range of tissue types (De Los Angeles *et al.* 2015). Given its crucial function, POU5F1 expression must be tightly regulated (Renčiuk *et al.* 2017). Research has unveiled the existence of multiple TFBSs within the POU5F1 gene’s promoter region, working collaboratively to regulate POU5F1 expression (Yeom *et al.* 1996). In this section, we attempted to leverage BertSNR to identify multiple types of TFBSs within the POU5F1 promoter region.

We acquired the genomic coordinates of the human POU5F1 promoter from the Eukaryotic Promoter Database (Dreos *et al.* 2017). The region of interest, considered as the promoter region for our study, encompassed a 1000-bp region both upstream and downstream from the central promoter point, totaling 2000 bp. Subsequently, we scanned this 2000-bp sequence using 188 distinct BertSNR identifying different TFBSs and annotated the exact locations of the identified TFBSs. As shown in Supplementary Fig. S7a, a total of 58 TFBSs were identified by BertSNR in the POU5F1 promoter region (Supplementary Table S12). To validate the reliability of the identified TFBSs, we visualized TF ChIP-seq clusters for the promoter region using the UCSC Genome Browser (https://genome.ucsc.edu/), as illustrated in Supplementary Fig. S7b. We found that the TFBSs identified by BertSNR, namely YY1, IKZF1, FOSL2, HNF4G, and ELF1, have corresponding ChIP-seq clusters in the promoter region, which are assembled from a large number of peaks of ChIP-seq experiments (Nassar *et al.* 2023). Moreover, many of the identified TFBSs in the POU5F1 promoter region aligned closely with prior research. Among the TFBSs identified by BertSNR, EGR1 regulates POU5F1 expression in human lung cancer (Feng *et al.* 2019); GATA1 can replace POU5F1 to reprogram cells into pluripotency (Shu *et al.* 2015); STAT3 promotes POU5F1 to regulate cellular self-renewal (Yin *et al.* 2015); ELF3 affects transcription of the POU5F1 gene (Park *et al.* 2014); and SOX2 and POU5F1 are linked through the Oct4/Sox2 complex to mutually regulate transcription (Chew *et al.* 2005). Additionally, our study unveiled several previously unstudied potential TFBSs. Collectively, these findings underscore the intricate nature of promoter formation, which is often subject to precise regulation through multiple TFBSs. In summary, BertSNR offers valuable insights into the study of TFBSs in promoter regions at single-nucleotide resolution.

## 4 Discussion

Identifying TFBSs in genomic sequences at a high-resolution level represents a fundamental challenge in contemporary research. Historically, a majority of prior efforts have been directed toward low-resolution TFBS identification. In this study, we introduce BertSNR, an innovative deep learning framework designed for the accurate identification of TFBSs at single-nucleotide resolution level, leveraging the capabilities of DNA language models. BertSNR is established combining a variety of cutting-edge techniques, including LLMs, attention mechanism, and multi-task learning. BertSNR effectively overcomes the constraints associated with PWM-based methods and CNN-based approaches, outperforming these methodologies across a set of 188 ChIP-seq TFBS datasets. Furthermore, our study delves into a comprehensive exploration of the model’s attention mechanism. These analyses involve dissecting the intricate decision-making processes within the model, scrutinizing the correlation between attention weights and motifs, and affirming the interpretability of the model. Additionally, we conduct a comparative analysis of the motifs generated by our model predictions against motifs contained in established databases. Finally, we successfully identified specific TFBSs within the POU5F1 promoter region, enhancing our understanding of the intricate regulation at single-nucleotide resolution within the non-coding region.

Despite BertSNR’s impressive performance, there remains room for further enhancement. Currently, our model is exclusively trained for a specific TF, limiting its capacity to identify binding sites of other TFs. In future endeavors, we can collect as many datasets of TFBSs as possible, and then fuse them, with each kind of TF grouped into a category. We can train a large model to predict various TFBSs at single-nucleotide resolution. In addition, recent investigations have unveiled the possibility of directly predicting epigenomic data, including TF ChIP-seq signals, histone modification signals, and ATAC-seq signals, from the underlying DNA sequences (Avsec *et al.* 2021). By integrating the data with our existing BertSNR model, we can potentially enhance the modeling of TFBSs.

## Supplementary Material

btae461_Supplementary_Data

## Data Availability

All the TFBS data can be found in the JASPAR database website (https://jaspar.elixir.no). We provided the ID of JASPAR data used in this study in Supplementary material.
