# Supplementary material for: BertSNR: an interpretable deep learning framework for single-nucleotide resolution identification of transcription factor binding sites based on DNA language model
Source: Bioinformatics. 2024 Aug 6;40(8):btae461. doi: 10.1093/bioinformatics/btae461 (PMC11310455; doi:10.1093/bioinformatics/btae461)
Supplement: btae461_Supplementary_Data [file btae461_supplementary_data.docx]

**Supplementary Materials**

**BertSNR: an interpretable deep learning framework for single nucleotide resolution identification of transcription factor binding sites based on DNA language model**

# Supplementary Methods

**Motifs discovery method**

The computational procedure of TF-MoDSW is delineated in Algorithm 1. Within this algorithm, LBrdr and RBrdr are utilized to denote the left and right endpoints of the sliding window, respectively. ContMo is indicative of the presence of motifs in the current sequence, while PLbl represents the predicted dense labels generated by deep learning models. MidPoint corresponds to the midpoint between the first positive window and the current window.

| **Algorithm 1** Motif Discovery Method (DnaSeq, MoLen, Cfds) |
| --- |
| **Input:** DnaSeq is the sequence which may contains TFBSs or not.  MoLen is the size of related TFBSs.  Cfds is the confidence for deciding window status.  **Output:** TFs-DNA binding motifs ${\{MotifSeq\}}_{i=1}^{\mathrm{Num}}$  01: LBrdr = 0 **and** RBrdr = LBrdr + MLen  02: ContMo, Num = 0  03: PLbl = D-AEDNet (DnaSeq)  04: **while** RBrdr <= len (DnaSeq) **do**  05: **if** PLbl [LBrdr: RBrdr] >= Cfds **then**  06: ContMo = 1  07: Treat current window as a positive one  08: Record LBrdr of first positive window and RBrdr of current window, **and** compute MidPoint  09: **end if**  10: LBrdr++ **and** RBrdr++  11: **if** current window is negative **and** prior window is positive **then**  12: $\mathrm{MotifSeq}^{++Num}$=DNASeq[MidPoint– 0.5 * MLen: MidPoint + 0.5 *Mlen]  13: Reset information of positive and negative windows  14: **end if**  15: **end while**  16: **if** ContMo ==0 **then**  17: **return** MLen * ‘N’  18: **else**  19: **return** ${\{MotifSeq\}}_{i=1}^{N}$  20: **end if** |

**Evaluation metrics**

To assess the model's performance comprehensively, we employ a diverse array of metrics encompassing accuracy (Acc), precision (Pre), recall (Rec), F1-score (F1-S), area under the receiver operating characteristic curve (AUC), and area under the precision-recall curve (AUPR). The calculation formula of them is as follows:

$$Accuracy=\frac{T_{+}+T_{-}}{T_{+}+T_{-}+F_{+}+F_{-}}$$

$$Recall=\frac{T_{+}}{T_{+}+F_{-}}$$

$$Precision=\frac{T_{+}}{T_{+}+F_{+}}$$

$$F1-score=2*\frac{Precision*Recall}{Precision+Recall}$$

where T_+, T_-, F_+ and F_- denote the numbers of true positives, true negatives, false positives and false negative, respectively. In addition, both AUC and AUPR provide valuable insights into the performance of classification models, with AUC emphasizing overall classification ability and AUPR focusing on precision and recall, which can be especially important in scenarios where class imbalance is a concern.

To facilitate a comparative analysis between the identified motifs and the ground truth, we also employ statistical metrics, including p-value, e-value, and q-value, to effectively showcase the performance of our developed motif discovery algorithm.

**p-value**: denotes probability which score of random matching is greater than or equal to $x$. The calculation formula of p-value is as follows:

$$p-value=P(X\geq x{|H}_{0},S)$$

where $S$ is reference motif spectrum.

**e-value**: denotes mathematical expectation of the number of motifs with score of random matching greater than $x$. The calculation formula of e-value is as follows:

$$e-value=p-value*n$$

where $n$ is number of candidate motifs in spectrum.

**q-value**: denotes minimum FDR (False Discovery Rate) threshold required to filter out the matching motifs with score of $x$. The calculation formula of q-value is as follows:

$$q-value={min}_{t\leq x}FDR(t)$$

where $FDR$ is mathematical expectation which the number of false discovered motifs account for proportion of total numbers of motifs in the set of motifs with a given score greater than or equal to $x$.

**Competing methods**

This paper centers its attention on the prediction of transcription factor binding sites with nucleotide-level. Accordingly, we have selected two methods, DeepSNR and D-AEDNet, which are also designed for nucleotide-level predictions, for the purpose of comparison. Furthermore, we extend our comparison to encompass traditional Matching methods. The following section delineates the three aforementioned methods in detail.

**Matching**: The Matching method commences by acquiring PWM data corresponding to the transcription factor of interest, sourced either from a pertinent database (e.g., JASPAR) or gleaned from relevant literature. The PWM assumes a matrix structure, where each row signifies a nucleotide base (A, C, G, T), and each column represents a specific position within the binding site. The numerical values within the PWM convey the significance or relative occurrence frequency of a given base at a particular position. Subsequently, the PWM is systematically traversed, employing a sliding-window approach, across the target sequence to identify regions exhibiting similarity to the PWM. At each juxtaposition of the PWM with the target sequence, a scoring mechanism is applied to quantify the extent of resemblance. A predefined threshold is then applied, designating positions that surpass this threshold as putative transcription factor binding sites.

**DeepSNR**: DeepSNR is the first deep learning model to predict TF binding sites from DNA sequences from scratch with single nucleotide resolution. It is inspired by image segmentation tasks. The model feeds a convolutional neural network with maximum pooling to extract features after one-hot encoding the input DNA sequence. Subsequently, the feature vector is fed back into the inverse convolutional neural network to recover the size of the original feature mapping, and finally a sigmoid function is applied to map each nucleotide output value to 0 or 1.

**D-AEDNet**: The D-AEDNet model consists of five components: an encoder network, a bridging network, a decoder network, an attention gate and a classifier. Sequences are first fed into an encoder network with convolutional operations as the basic component to learn binding site-related features. The encoder network encodes the feature map by gradually decreasing the feature space dimension and expanding the channel dimension. The spatial location and dependencies of each pixel in the feature map are subsequently maintained through a bridge network. Finally, the transcription factor binding sites in DNA sequences are localized in the decoder network by gradually increasing the spatial dimensions and decreasing the channel dimensions, and the final output is mapped to either 0 or 1 by learning thresholds in the classifier.

**Reference**

1. Salekin, S., Zhang, J.M. and Huang, Y. (2018) Base-pair resolution detection of transcription factor binding site by deep deconvolutional network. *Bioinformatics*, **34**, 3446-3453.

2. Zhang, Y., Wang, Z., Zeng, Y., Zhou, J. and Zou, Q. (2021) High-resolution transcription factor binding sites prediction improved performance and interpretability by deep learning method. *Briefings in Bioinformatics*, **22**, bbab273.

3. Kel, A.E., Gossling, E., Reuter, I., Cheremushkin, E., Kel-Margoulis, O.V. and Wingender, E. (2003) MATCHTM: a tool for searching transcription factor binding sites in DNA sequences. *Nucleic acids research*, **31**, 3576-3579.

# Supplementary Figure


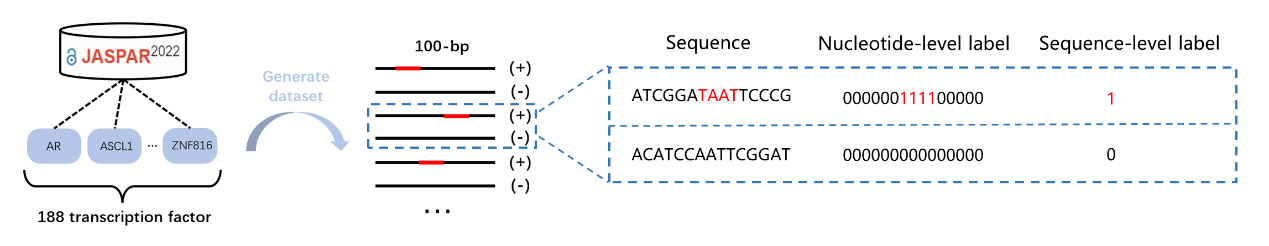


**Supplementary Figure S1. The training dataset consists of 188 transcription factor datasets collected from the JASPAR database. Each TFBS was randomly extended upstream and downstream to a total length of 100 bp to create sequence-level positive samples. These positive samples were then shuffled to generate sequence-level negative samples. All individual nucleotides within these sequences were labeled at the nucleotide level.**


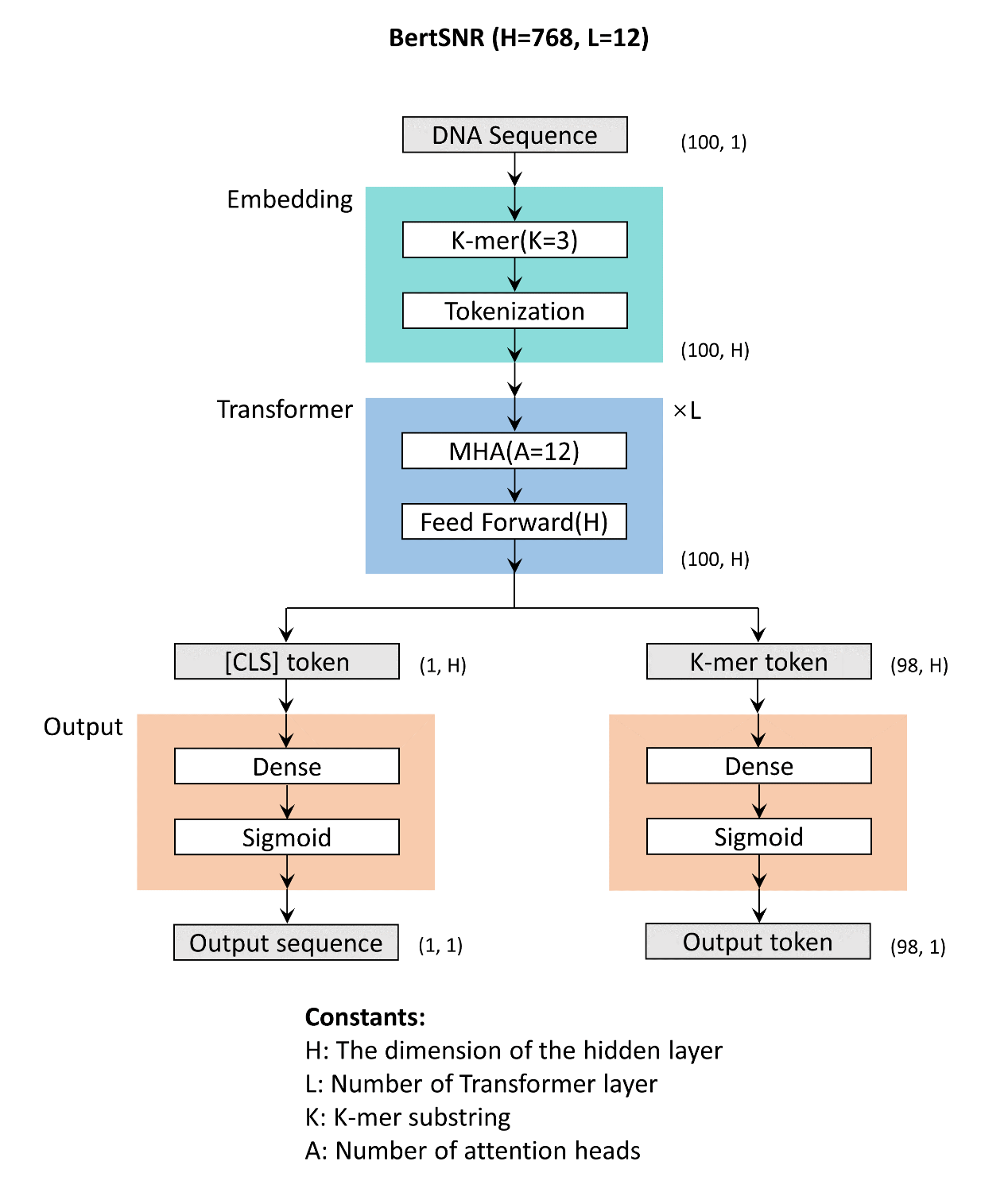


**Supplementary Figure S2. BertSNR model architecture.**


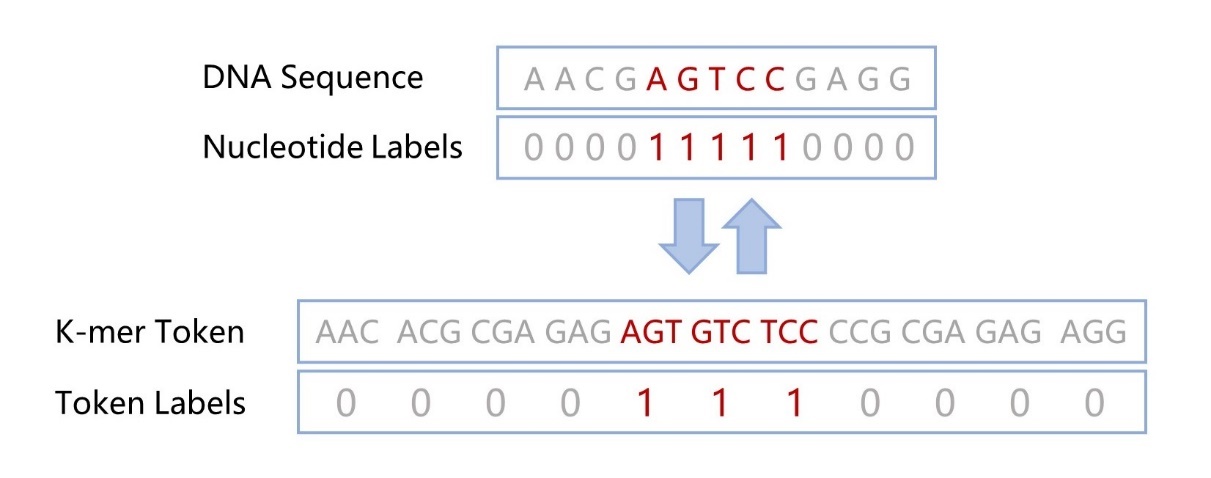


**Supplementary Figure S3. The criteria employed for the interconversion of nucleotide labels and token labels.**


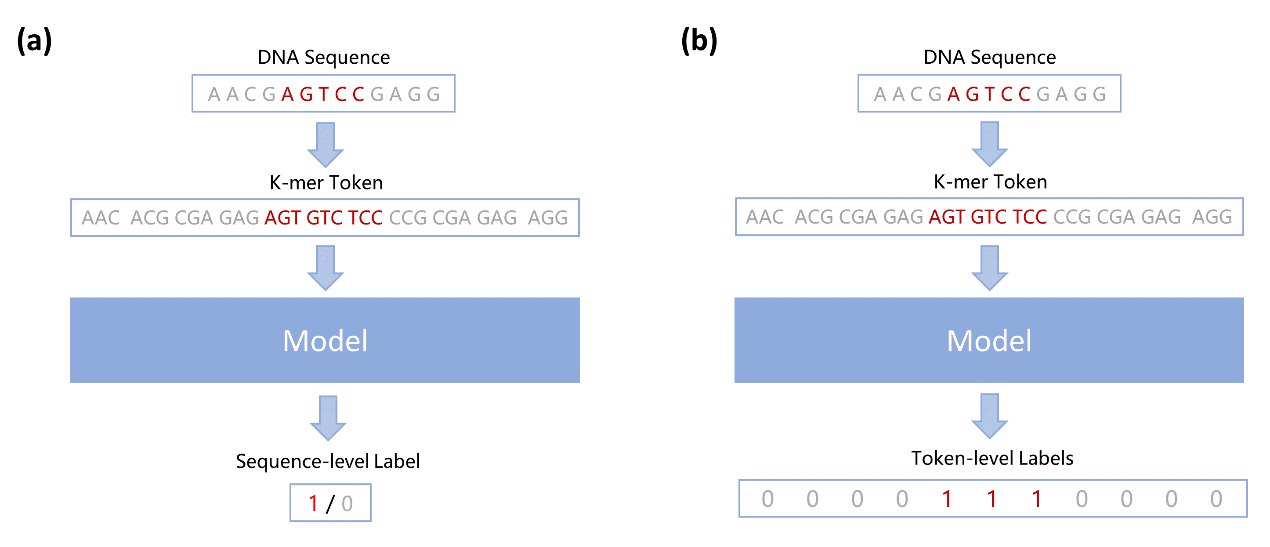


**Supplementary Figure S4. (a)** **Sequence-level classification involves determining the presence or absence of TFBSs within an entire DNA sequence. (b)** **Token-level classification involves predicting the precise locations of TFBSs at single nucleotide resolution within an entire DNA sequence.**


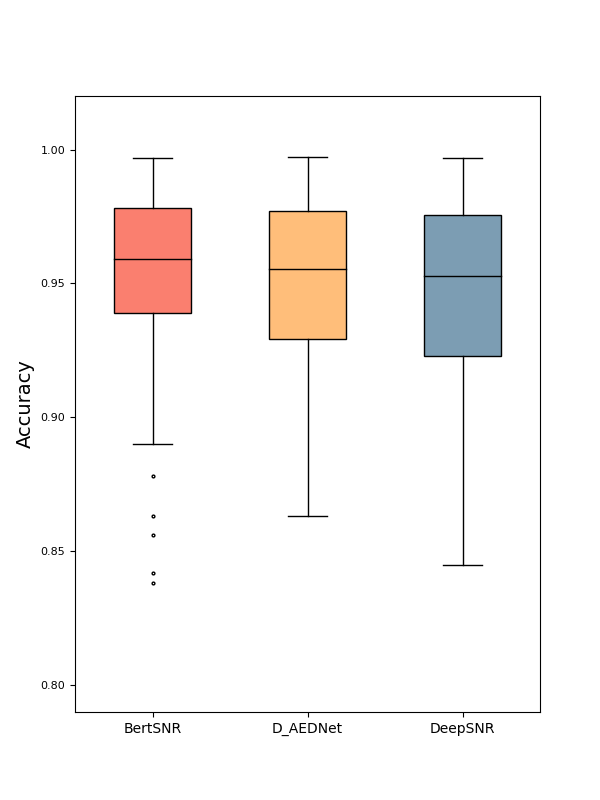
**Supplementary Figure S5. The distribution of accuracy across 188 TFs datasets for sequence-level TFBS prediction. For each box, the intermediate line indicates the median,and the top and bottom edges of the box indicate the upper and lower quartiles, respectively. The upper and lower sides indicate the maximum and minimum values.**


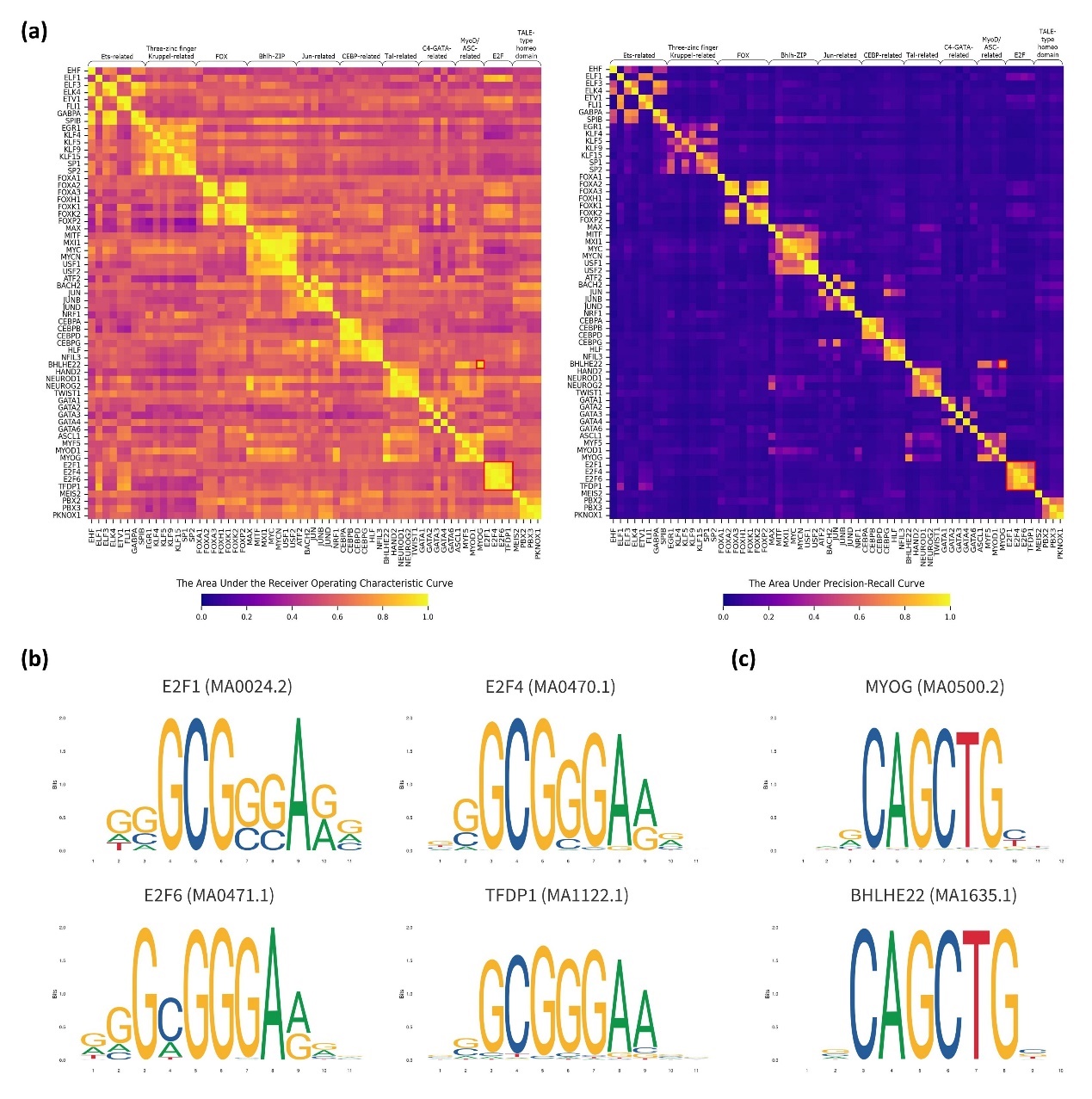


**Supplementary Figure S6. BertSNR predicts TFBSs across TFs. (a), The heatmap showing the values of AUC (left) and AUPR (right) cross-predicted on different TFs. (b), Comparison of the motif logos of the four types of TFBSs (E2F1, E2F4, E2F6 and TFDP1) from the E2F family. (c), Contrasting the motif logos of two types of TFBSs (MYOG and BHLHE22) from distinct families.**


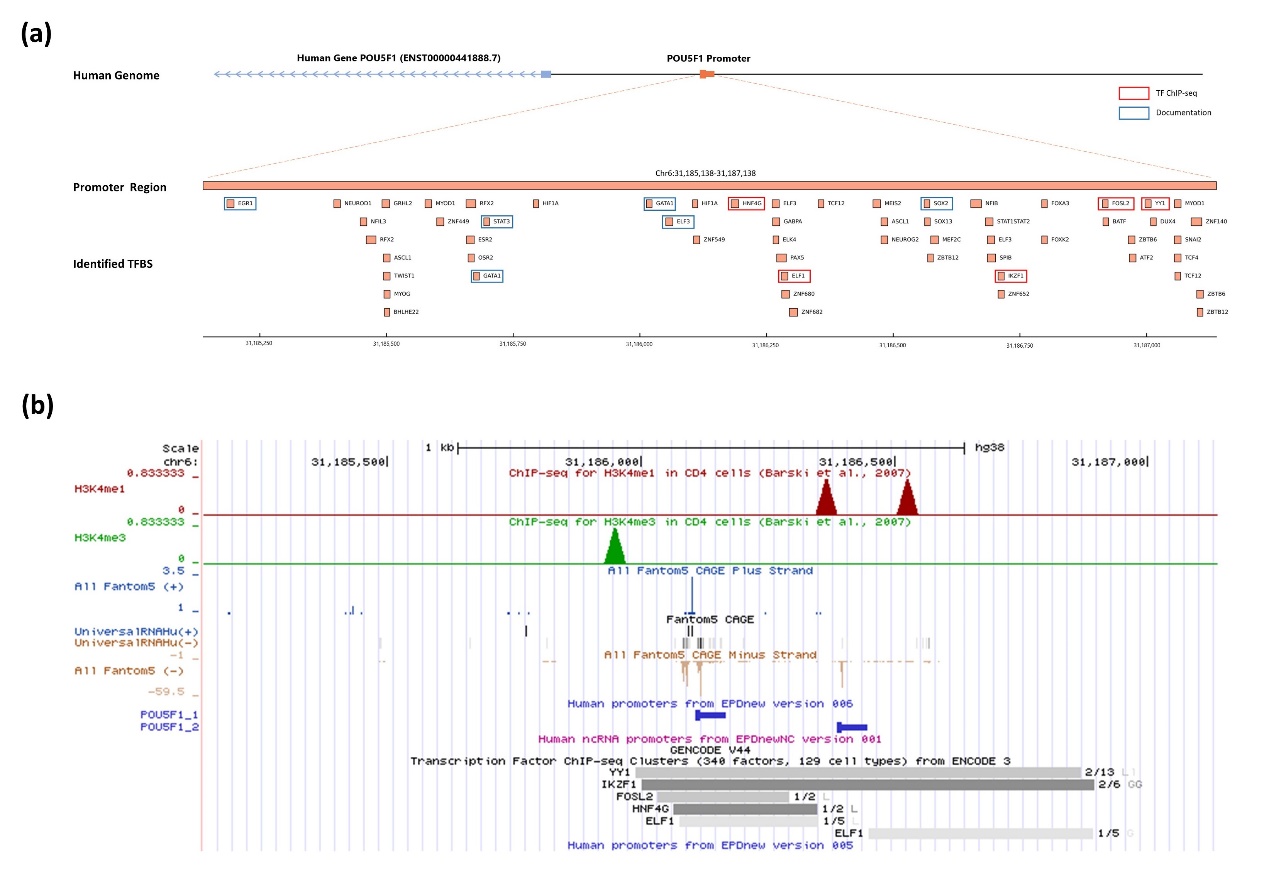


**Supplementary Figure S7. BertSNR identifies TFBSs in the POU5F1 promoter region at single nucleo-tide resolution. (a), Precise locations of TFBSs identified by BertSNR in selected POU5F1 promoter regions (chr6:31,185,138-31,187,138). (b), The visualisation of H3K4me1, H3K4me3, Fantom5 signal volume and TF ChIP-seq clusters in promoter regions using the UCSC Genome Browser.**

# Supplementary Table

**Supplementary Table S1. The source of the benchmarking dataset for transcription factor binding sites.**

| TFs | Data sources | Extra data sources |
| --- | --- | --- |
| AR | [MA0007.2](https://jaspar.genereg.net/matrix/MA0007.2) |  |
| ASCL1 | [MA1100.1](https://jaspar.genereg.net/matrix/MA1100.1) |  |
| ATF2 | [MA1632.1](https://jaspar.genereg.net/matrix/MA1632.1) |  |
| ATF4 | [MA0833.2](https://jaspar.genereg.net/matrix/MA0833.2) |  |
| BACH2 | [MA1101.1](https://jaspar.genereg.net/matrix/MA1101.1) |  |
| BATF | [MA1634.1](https://jaspar.genereg.net/matrix/MA1634.1) |  |
| BATF3 | [MA0835.2](https://jaspar.genereg.net/matrix/MA0835.2) |  |
| BATF::JUN | [MA0462.1](https://jaspar.genereg.net/matrix/MA0462.1) | [MA0462.2](https://jaspar.genereg.net/matrix/MA0462.2) |
| BHLHE22 | [MA1635.1](https://jaspar.genereg.net/matrix/MA1635.1) |  |
| CDX2 | [MA0465.1](https://jaspar.genereg.net/matrix/MA0465.1) | [MA0465.2](https://jaspar.genereg.net/matrix/MA0465.2) |
| CEBPA | [MA0102.3](https://jaspar.genereg.net/matrix/MA0102.3) | [MA0102.4](https://jaspar.genereg.net/matrix/MA0102.4) |
| CEBPB | [MA0466.1](https://jaspar.genereg.net/matrix/MA0466.1) |  |
| CEBPD | [MA0836.2](https://jaspar.genereg.net/matrix/MA0836.2) |  |
| CEBPG | [MA1636.1](https://jaspar.genereg.net/matrix/MA1636.1) |  |
| CREB1 | [MA0018.3](https://jaspar.genereg.net/matrix/MA0018.3) |  |
| CREM | [MA0609.2](https://jaspar.genereg.net/matrix/MA0609.2) |  |
| CTCFL | [MA1102.1](https://jaspar.genereg.net/matrix/MA1102.1) | [MA1102.2](https://jaspar.genereg.net/matrix/MA1102.2) |
| DUX4 | [MA0468.1](https://jaspar.genereg.net/matrix/MA0468.1) |  |
| E2F1 | [MA0024.2](https://jaspar.genereg.net/matrix/MA0024.2) |  |
| E2F4 | [MA0470.1](https://jaspar.genereg.net/matrix/MA0470.1) |  |
| E2F6 | [MA0471.1](https://jaspar.genereg.net/matrix/MA0471.1) | [MA0471.2](https://jaspar.genereg.net/matrix/MA0471.2) |
| EBF1 | [MA0154.2](https://jaspar.genereg.net/matrix/MA0154.2) | [MA0154.4](https://jaspar.genereg.net/matrix/MA0154.4) |
| EBF3 | [MA1637.1](https://jaspar.genereg.net/matrix/MA1637.1) |  |
| EGR1 | [MA0162.2](https://jaspar.genereg.net/matrix/MA0162.2) | [MA0162.4](https://jaspar.genereg.net/matrix/MA0162.4) |
| EHF | [MA0598.1](https://jaspar.genereg.net/matrix/MA0598.1) |  |
| ELF1 | [MA0473.1](https://jaspar.genereg.net/matrix/MA0473.1) | [MA0473.3](https://jaspar.genereg.net/matrix/MA0473.3) |
| ELF3 | [MA0640.2](https://jaspar.genereg.net/matrix/MA0640.2) |  |
| ELK4 | [MA0076.2](https://jaspar.genereg.net/matrix/MA0076.2) |  |
| ESR2 | [MA0258.2](https://jaspar.genereg.net/matrix/MA0258.2) |  |
| ETV1 | [MA0761.2](https://jaspar.genereg.net/matrix/MA0761.2) |  |
| FLI1 | [MA0475.1](https://jaspar.genereg.net/matrix/MA0475.1) |  |
| FOS | [MA0476.1](https://jaspar.genereg.net/matrix/MA0476.1) |  |
| FOSL1 | [MA0477.1](https://jaspar.genereg.net/matrix/MA0477.1) | [MA0477.2](https://jaspar.genereg.net/matrix/MA0477.2) |
| FOSL2 | [MA0478.1](https://jaspar.genereg.net/matrix/MA0478.1) |  |
| FOXA1 | [MA0148.3](https://jaspar.genereg.net/matrix/MA0148.3) | [MA0148.4](https://jaspar.genereg.net/matrix/MA0148.4) |
| FOXA2 | [MA0047.3](https://jaspar.genereg.net/matrix/MA0047.3) |  |
| FOXA3 | [MA1683.1](https://jaspar.genereg.net/matrix/MA1683.1) |  |
| FOXH1 | [MA0479.1](https://jaspar.genereg.net/matrix/MA0479.1) |  |
| FOXK1 | [MA0852.2](https://jaspar.genereg.net/matrix/MA0852.2) |  |
| FOXK2 | [MA1103.1](https://jaspar.genereg.net/matrix/MA1103.1) | [MA1103.2](https://jaspar.genereg.net/matrix/MA1103.2) |
| FOXP2 | [MA0593.1](https://jaspar.genereg.net/matrix/MA0593.1) |  |
| GABPA | [MA0062.3](https://jaspar.genereg.net/matrix/MA0062.3) |  |
| GATA1 | [MA0035.4](https://jaspar.genereg.net/matrix/MA0035.4) |  |
| GATA2 | [MA0036.2](https://jaspar.genereg.net/matrix/MA0036.2) | [MA0036.3](https://jaspar.genereg.net/matrix/MA0036.3) |
| GATA3 | [MA0037.2](https://jaspar.genereg.net/matrix/MA0037.2) |  |
| GATA4 | [MA0482.2](https://jaspar.genereg.net/matrix/MA0482.2) |  |
| GATA6 | [MA1104.1](https://jaspar.genereg.net/matrix/MA1104.1) | [MA1104.2](https://jaspar.genereg.net/matrix/MA1104.2) |
| GRHL2 | [MA1105.1](https://jaspar.genereg.net/matrix/MA1105.1) | [MA1105.2](https://jaspar.genereg.net/matrix/MA1105.2) |
| HAND2 | [MA1638.1](https://jaspar.genereg.net/matrix/MA1638.1) |  |
| HIF1A | [MA1106.1](https://jaspar.genereg.net/matrix/MA1106.1) |  |
| HLF | [MA0043.3](https://jaspar.genereg.net/matrix/MA0043.3) |  |
| HNF4A | [MA0114.2](https://jaspar.genereg.net/matrix/MA0114.2) | [MA0114.4](https://jaspar.genereg.net/matrix/MA0114.4) |
| HNF4G | [MA0484.1](https://jaspar.genereg.net/matrix/MA0484.1) | [MA0484.2](https://jaspar.genereg.net/matrix/MA0484.2) |
| HOXB13 | [MA0901.2](https://jaspar.genereg.net/matrix/MA0901.2) |  |
| IKZF1 | [MA1508.1](https://jaspar.genereg.net/matrix/MA1508.1) |  |
| IRF1 | [MA0050.2](https://jaspar.genereg.net/matrix/MA0050.2) |  |
| JUN | [MA0488.1](https://jaspar.genereg.net/matrix/MA0488.1) |  |
| JUNB | [MA0490.1](https://jaspar.genereg.net/matrix/MA0490.1) | [MA0490.2](https://jaspar.genereg.net/matrix/MA0490.2) |
| JUND | [MA0491.1](https://jaspar.genereg.net/matrix/MA0491.1) | [MA0491.2](https://jaspar.genereg.net/matrix/MA0491.2) |
| KLF15 | [MA1513.1](https://jaspar.genereg.net/matrix/MA1513.1) |  |
| KLF4 | [MA0039.3](https://jaspar.genereg.net/matrix/MA0039.3) | [MA0039.4](https://jaspar.genereg.net/matrix/MA0039.4) |
| KLF5 | [MA0599.1](https://jaspar.genereg.net/matrix/MA0599.1) |  |
| KLF9 | [MA1107.1](https://jaspar.genereg.net/matrix/MA1107.1) | [MA1107.2](https://jaspar.genereg.net/matrix/MA1107.2) |
| LHX2 | [MA0700.2](https://jaspar.genereg.net/matrix/MA0700.2) |  |
| MAFF | [MA0495.1](https://jaspar.genereg.net/matrix/MA0495.1) | [MA0495.3](https://jaspar.genereg.net/matrix/MA0495.3) |
| MAFG::NFE2L1 | [MA0089.2](https://jaspar.genereg.net/matrix/MA0089.2) |  |
| MAFK | [MA0496.1](https://jaspar.genereg.net/matrix/MA0496.1) | [MA0496.3](https://jaspar.genereg.net/matrix/MA0496.3) |
| MAF::NFE2 | [MA0501.1](https://jaspar.genereg.net/matrix/MA0501.1) |  |
| MAX | [MA0058.2](https://jaspar.genereg.net/matrix/MA0058.2) |  |
| MAZ | [MA1522.1](https://jaspar.genereg.net/matrix/MA1522.1) |  |
| MEF2A | [MA0052.2](https://jaspar.genereg.net/matrix/MA0052.2) | [MA0052.4](https://jaspar.genereg.net/matrix/MA0052.4) |
| MEF2C | [MA0497.1](https://jaspar.genereg.net/matrix/MA0497.1) |  |
| MEIS2 | [MA1640.1](https://jaspar.genereg.net/matrix/MA1640.1) |  |
| MITF | [MA0620.2](https://jaspar.genereg.net/matrix/MA0620.2) | [MA0620.3](https://jaspar.genereg.net/matrix/MA0620.3) |
| MXI1 | [MA1108.1](https://jaspar.genereg.net/matrix/MA1108.1) | [MA1108.2](https://jaspar.genereg.net/matrix/MA1108.2) |
| MYB | [MA0100.3](https://jaspar.genereg.net/matrix/MA0100.3) |  |
| MYC | [MA0147.3](https://jaspar.genereg.net/matrix/MA0147.3) |  |
| MYCN | [MA0104.4](https://jaspar.genereg.net/matrix/MA0104.4) |  |
| MYF5 | [MA1641.1](https://jaspar.genereg.net/matrix/MA1641.1) |  |
| MYOD1 | [MA0499.2](https://jaspar.genereg.net/matrix/MA0499.2) |  |
| MYOG | [MA0500.2](https://jaspar.genereg.net/matrix/MA0500.2) |  |
| MZF1 | [MA0056.2](https://jaspar.genereg.net/matrix/MA0056.2) |  |
| NEUROD1 | [MA1109.1](https://jaspar.genereg.net/matrix/MA1109.1) |  |
| NEUROG2 | [MA1642.1](https://jaspar.genereg.net/matrix/MA1642.1) |  |
| NFIB | [MA1643.1](https://jaspar.genereg.net/matrix/MA1643.1) |  |
| NFIC | [MA0161.2](https://jaspar.genereg.net/matrix/MA0161.2) |  |
| NFIL3 | [MA0025.2](https://jaspar.genereg.net/matrix/MA0025.2) |  |
| NFKB1 | [MA0105.3](https://jaspar.genereg.net/matrix/MA0105.3) |  |
| NFYA | [MA0060.2](https://jaspar.genereg.net/matrix/MA0060.2) | [MA0060.3](https://jaspar.genereg.net/matrix/MA0060.3) |
| NFYB | [MA0502.1](https://jaspar.genereg.net/matrix/MA0502.1) | [MA0502.2](https://jaspar.genereg.net/matrix/MA0502.2) |
| NFYC | [MA1644.1](https://jaspar.genereg.net/matrix/MA1644.1) |  |
| NKX2-2 | [MA1645.1](https://jaspar.genereg.net/matrix/MA1645.1) |  |
| NKX2-5 | [MA0063.2](https://jaspar.genereg.net/matrix/MA0063.2) |  |
| NR1H4 | [MA1110.1](https://jaspar.genereg.net/matrix/MA1110.1) |  |
| NR2F2 | [MA1111.1](https://jaspar.genereg.net/matrix/MA1111.1) |  |
| NR4A1 | [MA1112.1](https://jaspar.genereg.net/matrix/MA1112.1) | [MA1112.2](https://jaspar.genereg.net/matrix/MA1112.2) |
| NRF1 | [MA0506.1](https://jaspar.genereg.net/matrix/MA0506.1) |  |
| ONECUT1 | [MA0679.2](https://jaspar.genereg.net/matrix/MA0679.2) |  |
| OSR2 | [MA1646.1](https://jaspar.genereg.net/matrix/MA1646.1) |  |
| OTX2 | [MA0712.2](https://jaspar.genereg.net/matrix/MA0712.2) |  |
| PAX5 | [MA0014.2](https://jaspar.genereg.net/matrix/MA0014.2) |  |
| PBX2 | [MA1113.1](https://jaspar.genereg.net/matrix/MA1113.1) |  |
| PBX3 | [MA1114.1](https://jaspar.genereg.net/matrix/MA1114.1) |  |
| PHOX2B | [MA0681.2](https://jaspar.genereg.net/matrix/MA0681.2) |  |
| PKNOX1 | [MA0782.2](https://jaspar.genereg.net/matrix/MA0782.2) |  |
| POU2F2 | [MA0507.1](https://jaspar.genereg.net/matrix/MA0507.1) |  |
| POU2F3 | [MA0627.2](https://jaspar.genereg.net/matrix/MA0627.2) |  |
| POU5F1 | [MA1115.1](https://jaspar.genereg.net/matrix/MA1115.1) |  |
| PRDM1 | [MA0508.1](https://jaspar.genereg.net/matrix/MA0508.1) | [MA0508.3](https://jaspar.genereg.net/matrix/MA0508.3) |
| RBPJ | [MA1116.1](https://jaspar.genereg.net/matrix/MA1116.1) |  |
| RELB | [MA1117.1](https://jaspar.genereg.net/matrix/MA1117.1) |  |
| RFX1 | [MA0509.2](https://jaspar.genereg.net/matrix/MA0509.2) |  |
| RFX2 | [MA0600.1](https://jaspar.genereg.net/matrix/MA0600.1) |  |
| RFX5 | [MA0510.1](https://jaspar.genereg.net/matrix/MA0510.1) |  |
| RUNX2 | [MA0511.1](https://jaspar.genereg.net/matrix/MA0511.1) |  |
| RUNX3 | [MA0684.2](https://jaspar.genereg.net/matrix/MA0684.2) |  |
| SCRT1 | [MA0743.2](https://jaspar.genereg.net/matrix/MA0743.2) |  |
| SCRT2 | [MA0744.2](https://jaspar.genereg.net/matrix/MA0744.2) |  |
| SIX1 | [MA1118.1](https://jaspar.genereg.net/matrix/MA1118.1) |  |
| SIX2 | [MA1119.1](https://jaspar.genereg.net/matrix/MA1119.1) |  |
| SNAI2 | [MA0745.2](https://jaspar.genereg.net/matrix/MA0745.2) |  |
| SOX10 | [MA0442.2](https://jaspar.genereg.net/matrix/MA0442.2) |  |
| SOX13 | [MA1120.1](https://jaspar.genereg.net/matrix/MA1120.1) |  |
| SOX2 | [MA0143.4](https://jaspar.genereg.net/matrix/MA0143.4) |  |
| SP1 | [MA0079.3](https://jaspar.genereg.net/matrix/MA0079.3) |  |
| SP2 | [MA0516.1](https://jaspar.genereg.net/matrix/MA0516.1) |  |
| SPIB | [MA0081.2](https://jaspar.genereg.net/matrix/MA0081.2) |  |
| SRF | [MA0083.2](https://jaspar.genereg.net/matrix/MA0083.2) |  |
| STAT1 | [MA0137.3](https://jaspar.genereg.net/matrix/MA0137.3) |  |
| STAT1::STAT2 | [MA0517.1](https://jaspar.genereg.net/matrix/MA0517.1) |  |
| STAT3 | [MA0144.2](https://jaspar.genereg.net/matrix/MA0144.2) |  |
| TCF12 | [MA1648.1](https://jaspar.genereg.net/matrix/MA1648.1) |  |
| TCF3 | [MA0522.3](https://jaspar.genereg.net/matrix/MA0522.3) |  |
| TCF4 | [MA0830.2](https://jaspar.genereg.net/matrix/MA0830.2) |  |
| TCF7 | [MA0769.2](https://jaspar.genereg.net/matrix/MA0769.2) |  |
| TCF7L2 | [MA0523.1](https://jaspar.genereg.net/matrix/MA0523.1) |  |
| TEAD1 | [MA0090.3](https://jaspar.genereg.net/matrix/MA0090.3) |  |
| TEAD2 | [MA1121.1](https://jaspar.genereg.net/matrix/MA1121.1) |  |
| TEAD4 | [MA0809.2](https://jaspar.genereg.net/matrix/MA0809.2) |  |
| TFAP2A | [MA0003.2](https://jaspar.genereg.net/matrix/MA0003.2) | [MA0003.4](https://jaspar.genereg.net/matrix/MA0003.4) |
| TFAP2C | [MA0524.1](https://jaspar.genereg.net/matrix/MA0524.1) |  |
| TFDP1 | [MA1122.1](https://jaspar.genereg.net/matrix/MA1122.1) |  |
| TP53 | [MA0106.2](https://jaspar.genereg.net/matrix/MA0106.2) |  |
| TP63 | [MA0525.1](https://jaspar.genereg.net/matrix/MA0525.1) |  |
| TWIST1 | [MA1123.1](https://jaspar.genereg.net/matrix/MA1123.1) |  |
| USF1 | [MA0093.2](https://jaspar.genereg.net/matrix/MA0093.2) | [MA0093.3](https://jaspar.genereg.net/matrix/MA0093.3) |
| USF2 | [MA0526.1](https://jaspar.genereg.net/matrix/MA0526.1) | [MA0526.3](https://jaspar.genereg.net/matrix/MA0526.3) |
| YY1 | [MA0095.2](https://jaspar.genereg.net/matrix/MA0095.2) |  |
| YY2 | [MA0748.2](https://jaspar.genereg.net/matrix/MA0748.2) |  |
| ZBTB12 | [MA1649.1](https://jaspar.genereg.net/matrix/MA1649.1) |  |
| ZBTB14 | [MA1650.1](https://jaspar.genereg.net/matrix/MA1650.1) |  |
| ZBTB26 | [MA1579.1](https://jaspar.genereg.net/matrix/MA1579.1) |  |
| ZBTB33 | [MA0527.1](https://jaspar.genereg.net/matrix/MA0527.1) |  |
| ZBTB6 | [MA1581.1](https://jaspar.genereg.net/matrix/MA1581.1) |  |
| ZBTB7A | [MA0750.2](https://jaspar.genereg.net/matrix/MA0750.2) |  |
| ZEB1 | [MA0103.2](https://jaspar.genereg.net/matrix/MA0103.2) | [MA0103.3](https://jaspar.genereg.net/matrix/MA0103.3) |
| ZFP42 | [MA1651.1](https://jaspar.genereg.net/matrix/MA1651.1) |  |
| ZFP57 | [MA1583.1](https://jaspar.genereg.net/matrix/MA1583.1) |  |
| ZIM3 | [MA1709.1](https://jaspar.genereg.net/matrix/MA1709.1) |  |
| ZKSCAN1 | [MA1585.1](https://jaspar.genereg.net/matrix/MA1585.1) |  |
| ZKSCAN5 | [MA1652.1](https://jaspar.genereg.net/matrix/MA1652.1) |  |
| ZNF135 | [MA1587.1](https://jaspar.genereg.net/matrix/MA1587.1) |  |
| ZNF136 | [MA1588.1](https://jaspar.genereg.net/matrix/MA1588.1) |  |
| ZNF140 | [MA1589.1](https://jaspar.genereg.net/matrix/MA1589.1) |  |
| ZNF148 | [MA1653.1](https://jaspar.genereg.net/matrix/MA1653.1) |  |
| ZNF16 | [MA1654.1](https://jaspar.genereg.net/matrix/MA1654.1) |  |
| ZNF189 | [MA1725.1](https://jaspar.genereg.net/matrix/MA1725.1) |  |
| ZNF24 | [MA1124.1](https://jaspar.genereg.net/matrix/MA1124.1) |  |
| ZNF263 | [MA0528.1](https://jaspar.genereg.net/matrix/MA0528.1) |  |
| ZNF317 | [MA1593.1](https://jaspar.genereg.net/matrix/MA1593.1) |  |
| ZNF331 | [MA1726.1](https://jaspar.genereg.net/matrix/MA1726.1) |  |
| ZNF341 | [MA1655.1](https://jaspar.genereg.net/matrix/MA1655.1) |  |
| ZNF382 | [MA1594.1](https://jaspar.genereg.net/matrix/MA1594.1) |  |
| ZNF384 | [MA1125.1](https://jaspar.genereg.net/matrix/MA1125.1) |  |
| ZNF449 | [MA1656.1](https://jaspar.genereg.net/matrix/MA1656.1) |  |
| ZNF460 | [MA1596.1](https://jaspar.genereg.net/matrix/MA1596.1) |  |
| ZNF528 | [MA1597.1](https://jaspar.genereg.net/matrix/MA1597.1) |  |
| ZNF549 | [MA1728.1](https://jaspar.genereg.net/matrix/MA1728.1) |  |
| ZNF610 | [MA1713.1](https://jaspar.genereg.net/matrix/MA1713.1) |  |
| ZNF652 | [MA1657.1](https://jaspar.genereg.net/matrix/MA1657.1) |  |
| ZNF680 | [MA1729.1](https://jaspar.genereg.net/matrix/MA1729.1) |  |
| ZNF682 | [MA1599.1](https://jaspar.genereg.net/matrix/MA1599.1) |  |
| ZNF707 | [MA1715.1](https://jaspar.genereg.net/matrix/MA1715.1) |  |
| ZNF708 | [MA1730.1](https://jaspar.genereg.net/matrix/MA1730.1) |  |
| ZNF8 | [MA1718.1](https://jaspar.genereg.net/matrix/MA1718.1) |  |
| ZNF816 | [MA1719.1](https://jaspar.genereg.net/matrix/MA1719.1) |  |
| ZNF85 | [MA1720.1](https://jaspar.genereg.net/matrix/MA1720.1) |  |
| ZNF93 | [MA1721.1](https://jaspar.genereg.net/matrix/MA1721.1) |  |

**Supplementary Table S2. Comprehensive details pertaining to the** **188 transcription factor binding site datasets.**

| TFs | Sequence(train) | Sequence(test) | Nucleotide (train) | Nucleotide (test) | Pos:Neg |
| --- | --- | --- | --- | --- | --- |
| AR | 17930 | 4482 | 1793000 | 448200 | 1:12 |
| ASCL1 | 11284 | 2820 | 1128400 | 282000 | 1:14 |
| ATF2 | 95005 | 23751 | 9500500 | 2375100 | 1:14 |
| ATF4 | 53220 | 13304 | 5322000 | 1330400 | 1:13 |
| BACH2 | 3032 | 758 | 303200 | 75800 | 1:13 |
| BATF | 75191 | 18797 | 7519100 | 1879700 | 1:17 |
| BATF3 | 19143 | 4785 | 1914300 | 478500 | 1:17 |
| BATFJUN | 16836 | 4208 | 1683600 | 420800 | 1:17 |
| BHLHE22 | 29370 | 7342 | 2937000 | 734200 | 1:19 |
| CDX2 | 2556 | 638 | 255600 | 63800 | 1:17 |
| CEBPA | 24509 | 6127 | 2450900 | 612700 | 1:17 |
| CEBPB | 159191 | 39797 | 15919100 | 3979700 | 1:17 |
| CEBPD | 34322 | 8580 | 3432200 | 858000 | 1:14 |
| CEBPG | 59687 | 14921 | 5968700 | 1492100 | 1:12 |
| CREB1 | 14196 | 3548 | 1419600 | 354800 | 1:15 |
| CREM | 27244 | 6810 | 2724400 | 681000 | 1:11 |
| CTCFL | 14112 | 3528 | 1411200 | 352800 | 1:13 |
| DUX4 | 61148 | 15286 | 6114800 | 1528600 | 1:17 |
| E2F1 | 1695 | 423 | 169500 | 42300 | 1:17 |
| E2F4 | 3005 | 751 | 300500 | 75100 | 1:17 |
| E2F6 | 4412 | 1102 | 441200 | 110200 | 1:17 |
| EBF1 | 54168 | 13542 | 5416800 | 1354200 | 1:17 |
| EBF3 | 35298 | 8824 | 3529800 | 882400 | 1:14 |
| EGR1 | 19610 | 4902 | 1961000 | 490200 | 1:13 |
| EHF | 2284 | 570 | 228400 | 57000 | 1:24 |
| ELF1 | 21629 | 5407 | 2162900 | 540700 | 1:14 |
| ELF3 | 77596 | 19398 | 7759600 | 1939800 | 1:13 |
| ELK4 | 5484 | 1370 | 548400 | 137000 | 1:17 |
| ESR2 | 13189 | 3297 | 1318900 | 329700 | 1:12 |
| ETV1 | 59732 | 14932 | 5973200 | 1493200 | 1:13 |
| FLI1 | 5868 | 1466 | 586800 | 146600 | 1:17 |
| FOS | 47034 | 11758 | 4703400 | 1175800 | 1:17 |
| FOSL1 | 8436 | 2108 | 843600 | 210800 | 1:17 |
| FOSL2 | 8509 | 2127 | 850900 | 212700 | 1:17 |
| FOXA1 | 35213 | 8803 | 3521300 | 880300 | 1:12 |
| FOXA2 | 419397 | 104849 | 41939700 | 10484900 | 1:17 |
| FOXA3 | 78293 | 19573 | 7829300 | 1957300 | 1:17 |
| FOXH1 | 13138 | 3284 | 1313800 | 328400 | 1:17 |
| FOXK1 | 1690 | 422 | 169000 | 42200 | 1:13 |
| FOXK2 | 12764 | 3190 | 1276400 | 319000 | 1:17 |
| FOXP2 | 1226 | 306 | 122600 | 30600 | 1:17 |
| GABPA | 104760 | 26190 | 10476000 | 2619000 | 1:13 |
| GATA1 | 114893 | 28723 | 11489300 | 2872300 | 1:17 |
| GATA2 | 7008 | 1752 | 700800 | 175200 | 1:13 |
| GATA3 | 7405 | 1851 | 740500 | 185100 | 1:24 |
| GATA4 | 94418 | 23604 | 9441800 | 2360400 | 1:15 |
| GATA6 | 29840 | 7460 | 2984000 | 746000 | 1:14 |
| GRHL2 | 21404 | 5350 | 2140400 | 535000 | 1:12 |
| HAND2 | 48452 | 12112 | 4845200 | 1211200 | 1:19 |
| HIF1A | 1568 | 392 | 156800 | 39200 | 1:19 |
| HLF | 39076 | 9768 | 3907600 | 976800 | 1:13 |
| HNF4A | 26829 | 6707 | 2682900 | 670700 | 1:12 |
| HNF4G | 15124 | 3780 | 1512400 | 378000 | 1:12 |
| HOXB13 | 125912 | 31478 | 12591200 | 3147800 | 1:13 |
| IKZF1 | 17469 | 4367 | 1746900 | 436700 | 1:15 |
| IRF1 | 2180 | 544 | 218000 | 54400 | 1:8 |
| JUN | 33549 | 8387 | 3354900 | 838700 | 1:14 |
| JUNB | 27188 | 6796 | 2718800 | 679600 | 1:17 |
| JUND | 61936 | 15484 | 6193600 | 1548400 | 1:17 |
| KLF15 | 18191 | 4547 | 1819100 | 454700 | 1:17 |
| KLF4 | 1551 | 387 | 155100 | 38700 | 1:17 |
| KLF5 | 21778 | 5444 | 2177800 | 544400 | 1:18 |
| KLF9 | 9207 | 2301 | 920700 | 230100 | 1:14 |
| LHX2 | 10132 | 2532 | 1013200 | 253200 | 1:17 |
| MAFF | 86013 | 21503 | 8601300 | 2150300 | 1:10 |
| MAFGNFE2L1 | 5370 | 1342 | 537000 | 134200 | 1:11 |
| MAFK | 97264 | 24316 | 9726400 | 2431600 | 1:12 |
| MAFNFE2 | 1744 | 436 | 174400 | 43600 | 1:12 |
| MAX | 39304 | 9826 | 3930400 | 982600 | 1:19 |
| MAZ | 24101 | 6025 | 2410100 | 602500 | 1:17 |
| MEF2A | 2357 | 589 | 235700 | 58900 | 1:12 |
| MEF2C | 3535 | 883 | 353500 | 88300 | 1:12 |
| MEIS2 | 32277 | 8069 | 3227700 | 806900 | 1:12 |
| MITF | 38316 | 9578 | 3831600 | 957800 | 1:10 |
| MXI1 | 5925 | 1481 | 592500 | 148100 | 1:14 |
| MYB | 2442 | 610 | 244200 | 61000 | 1:19 |
| MYC | 7908 | 1976 | 790800 | 197600 | 1:15 |
| MYCN | 11175 | 2793 | 1117500 | 279300 | 1:15 |
| MYF5 | 17936 | 4484 | 1793600 | 448400 | 1:15 |
| MYOD1 | 54879 | 13719 | 5487900 | 1371900 | 1:14 |
| MYOG | 35772 | 8942 | 3577200 | 894200 | 1:15 |
| MZF1 | 13183 | 3295 | 1318300 | 329500 | 1:14 |
| NEUROD1 | 3652 | 912 | 365200 | 91200 | 1:14 |
| NEUROG2 | 55026 | 13756 | 5502600 | 1375600 | 1:14 |
| NFIB | 7066 | 1766 | 706600 | 176600 | 1:8 |
| NFIC | 18572 | 4642 | 1857200 | 464200 | 1:17 |
| NFIL3 | 36783 | 9195 | 3678300 | 919500 | 1:14 |
| NFKB1 | 8180 | 2044 | 818000 | 204400 | 1:17 |
| NFYA | 14029 | 3507 | 1402900 | 350700 | 1:10 |
| NFYB | 11232 | 2808 | 1123200 | 280800 | 1:12 |
| NFYC | 21780 | 5444 | 2178000 | 544400 | 1:17 |
| NKX2-2 | 41637 | 10409 | 4163700 | 1040900 | 1:13 |
| NKX2-5 | 8711 | 2177 | 871100 | 217700 | 1:17 |
| NR1H4 | 808 | 202 | 80800 | 20200 | 1:17 |
| NR2F2 | 3527 | 881 | 352700 | 88100 | 1:17 |
| NR4A1 | 13410 | 3352 | 1341000 | 335200 | 1:19 |
| NRF1 | 7399 | 1849 | 739900 | 184900 | 1:17 |
| ONECUT1 | 99549 | 24887 | 9954900 | 2488700 | 1:11 |
| OSR2 | 23692 | 5922 | 2369200 | 592200 | 1:15 |
| OTX2 | 193072 | 48268 | 19307200 | 4826800 | 1:15 |
| PAX5 | 1434 | 358 | 143400 | 35800 | 1:9 |
| PBX2 | 3992 | 998 | 399200 | 99800 | 1:15 |
| PBX3 | 11215 | 2803 | 1121500 | 280300 | 1:10 |
| PHOX2B | 85176 | 21294 | 8517600 | 2129400 | 1:11 |
| PKNOX1 | 63933 | 15983 | 6393300 | 1598300 | 1:12 |
| POU2F2 | 3660 | 914 | 366000 | 91400 | 1:14 |
| POU2F3 | 89642 | 22410 | 8964200 | 2241000 | 1:14 |
| POU5F1 | 20983 | 5245 | 2098300 | 524500 | 1:17 |
| PRDM1 | 7365 | 1841 | 736500 | 184100 | 1:12 |
| RBPJ | 3844 | 960 | 384400 | 96000 | 1:19 |
| RELB | 5250 | 1312 | 525000 | 131200 | 1:17 |
| RFX1 | 28783 | 7195 | 2878300 | 719500 | 1:13 |
| RFX2 | 3754 | 938 | 375400 | 93800 | 1:9 |
| RFX5 | 6189 | 1547 | 618900 | 154700 | 1:12 |
| RUNX2 | 1700 | 424 | 170000 | 42400 | 1:12 |
| RUNX3 | 43800 | 10950 | 4380000 | 1095000 | 1:15 |
| SCRT1 | 96322 | 24080 | 9632200 | 2408000 | 1:11 |
| SCRT2 | 104976 | 26244 | 10497600 | 2624400 | 1:11 |
| SIX1 | 1773 | 443 | 177300 | 44300 | 1:17 |
| SIX2 | 8725 | 2181 | 872500 | 218100 | 1:11 |
| SNAI2 | 73306 | 18326 | 7330600 | 1832600 | 1:14 |
| SOX10 | 3288 | 822 | 328800 | 82200 | 1:17 |
| SOX13 | 7284 | 1820 | 728400 | 182000 | 1:17 |
| SOX2 | 137197 | 34299 | 13719700 | 3429900 | 1:17 |
| SP1 | 13975 | 3493 | 1397500 | 349300 | 1:17 |
| SP2 | 2698 | 674 | 269800 | 67400 | 1:12 |
| SPIB | 43085 | 10771 | 4308500 | 1077100 | 1:11 |
| SRF | 3644 | 910 | 364400 | 91000 | 1:10 |
| STAT1 | 5807 | 1451 | 580700 | 145100 | 1:17 |
| STAT1STAT2 | 992 | 248 | 99200 | 24800 | 1:12 |
| STAT3 | 34592 | 8648 | 3459200 | 864800 | 1:17 |
| TCF12 | 99000 | 24750 | 9900000 | 2475000 | 1:17 |
| TCF3 | 50013 | 12503 | 5001300 | 1250300 | 1:17 |
| TCF4 | 47138 | 11784 | 4713800 | 1178400 | 1:14 |
| TCF7 | 23986 | 5996 | 2398600 | 599600 | 1:17 |
| TCF7L2 | 6701 | 1675 | 670100 | 167500 | 1:13 |
| TEAD1 | 112512 | 28128 | 11251200 | 2812800 | 1:14 |
| TEAD2 | 847 | 211 | 84700 | 21100 | 1:14 |
| TEAD4 | 124858 | 31214 | 12485800 | 3121400 | 1:15 |
| TFAP2A | 8157 | 2039 | 815700 | 203900 | 1:12 |
| TFAP2C | 29482 | 7370 | 2948200 | 737000 | 1:12 |
| TFDP1 | 3208 | 802 | 320800 | 80200 | 1:17 |
| TP53 | 1970 | 492 | 197000 | 49200 | 1:12 |
| TP63 | 15412 | 3852 | 1541200 | 385200 | 1:9 |
| TWIST1 | 29023 | 7255 | 2902300 | 725500 | 1:14 |
| USF1 | 26948 | 6736 | 2694800 | 673600 | 1:17 |
| USF2 | 22111 | 5527 | 2211100 | 552700 | 1:17 |
| YY1 | 11474 | 2868 | 1147400 | 286800 | 1:15 |
| YY2 | 9124 | 2280 | 912400 | 228000 | 1:17 |
| ZBTB12 | 7608 | 1902 | 760800 | 190200 | 1:17 |
| ZBTB14 | 7276 | 1818 | 727600 | 181800 | 1:15 |
| ZBTB26 | 2604 | 650 | 260400 | 65000 | 1:12 |
| ZBTB33 | 1128 | 282 | 112800 | 28200 | 1:12 |
| ZBTB6 | 3564 | 890 | 356400 | 89000 | 1:14 |
| ZBTB7A | 23418 | 5854 | 2341800 | 585400 | 1:14 |
| ZEB1 | 5688 | 1422 | 568800 | 142200 | 1:21 |
| ZFP42 | 2802 | 700 | 280200 | 70000 | 1:8 |
| ZFP57 | 10132 | 2532 | 1013200 | 253200 | 1:14 |
| ZIM3 | 27552 | 6888 | 2755200 | 688800 | 1:12 |
| ZKSCAN1 | 9804 | 2450 | 980400 | 245000 | 1:19 |
| ZKSCAN5 | 5324 | 1330 | 532400 | 133000 | 1:13 |
| ZNF135 | 1517 | 379 | 151700 | 37900 | 1:13 |
| ZNF136 | 5122 | 1280 | 512200 | 128000 | 1:12 |
| ZNF140 | 2944 | 736 | 294400 | 73600 | 1:8 |
| ZNF148 | 17911 | 4477 | 1791100 | 447700 | 1:15 |
| ZNF16 | 1333 | 333 | 133300 | 33300 | 1:7 |
| ZNF189 | 25514 | 6378 | 2551400 | 637800 | 1:14 |
| ZNF24 | 37287 | 9321 | 3728700 | 932100 | 1:14 |
| ZNF263 | 24376 | 6094 | 2437600 | 609400 | 1:8 |
| ZNF317 | 18341 | 4585 | 1834100 | 458500 | 1:15 |
| ZNF331 | 1844 | 460 | 184400 | 46000 | 1:13 |
| ZNF341 | 29960 | 7490 | 2996000 | 749000 | 1:15 |
| ZNF382 | 6804 | 1700 | 680400 | 170000 | 1:7 |
| ZNF384 | 48672 | 12168 | 4867200 | 1216800 | 1:15 |
| ZNF449 | 12253 | 3063 | 1225300 | 306300 | 1:13 |
| ZNF460 | 2580 | 644 | 258000 | 64400 | 1:11 |
| ZNF528 | 986 | 246 | 98600 | 24600 | 1:10 |
| ZNF549 | 7720 | 1930 | 772000 | 193000 | 1:15 |
| ZNF610 | 3818 | 954 | 381800 | 95400 | 1:13 |
| ZNF652 | 19797 | 4949 | 1979700 | 494900 | 1:15 |
| ZNF680 | 9221 | 2305 | 922100 | 230500 | 1:12 |
| ZNF682 | 1693 | 423 | 169300 | 42300 | 1:11 |
| ZNF707 | 1852 | 462 | 185200 | 46200 | 1:12 |
| ZNF708 | 1359 | 339 | 135900 | 33900 | 1:12 |
| ZNF8 | 4304 | 1076 | 430400 | 107600 | 1:9 |
| ZNF816 | 1685 | 421 | 168500 | 42100 | 1:10 |
| ZNF85 | 935 | 233 | 93500 | 23300 | 1:11 |
| ZNF93 | 3821 | 955 | 382100 | 95500 | 1:11 |

**Supplementary Table S3. The optimal hyper-parameters of BertSNR and the corresponding search space.**

| Calibration parameters | Search space | Best parameters |
| --- | --- | --- |
| Learning rate | [0.001, 0.0001] | 0.0002 |
| K(-mer) | {3, 4, 5, 6} | 3 |
| $\lambda$ | [0.05, 0.95] | 0.15 |
| Batch size | {16, 32} | 32 |
| Threshold | {0.4, 0.5, 0.6} | 0.5 |
| Dropout | {0.1, 0.2, 0.3} | 0.2 |
| Optimizer | {Adam, AdamW} | AdamW |

**Supplementary Table S4. The performance evaluation of BertSNR on the 188 transcription factor binding site datasets.**

| TFs | Acc | Pre | Rec | F1-S | AUC | AUPR |
| --- | --- | --- | --- | --- | --- | --- |
| AR | 0.993 | 0.926 | 0.99 | 0.956 | 0.999 | 0.992 |
| ASCL1 | 0.984 | 0.831 | 0.955 | 0.888 | 0.996 | 0.95 |
| ATF2 | 0.995 | 0.949 | 0.977 | 0.963 | 0.999 | 0.991 |
| ATF4 | 0.992 | 0.919 | 0.968 | 0.943 | 0.998 | 0.981 |
| BACH2 | 0.986 | 0.85 | 0.965 | 0.904 | 0.995 | 0.964 |
| BATF | 0.991 | 0.902 | 0.929 | 0.915 | 0.997 | 0.96 |
| BATF3 | 0.99 | 0.899 | 0.916 | 0.907 | 0.996 | 0.957 |
| BATFJUN | 0.993 | 0.941 | 0.929 | 0.935 | 0.998 | 0.982 |
| BHLHE22 | 0.986 | 0.85 | 0.882 | 0.866 | 0.995 | 0.939 |
| CDX2 | 0.99 | 0.855 | 0.985 | 0.915 | 0.999 | 0.976 |
| CEBPA | 0.996 | 0.94 | 0.988 | 0.963 | 1.0 | 0.993 |
| CEBPB | 0.994 | 0.9 | 0.996 | 0.945 | 0.999 | 0.988 |
| CEBPD | 0.989 | 0.869 | 0.986 | 0.924 | 0.998 | 0.982 |
| CEBPG | 0.988 | 0.874 | 0.984 | 0.925 | 0.998 | 0.982 |
| CREB1 | 0.99 | 0.916 | 0.91 | 0.913 | 0.998 | 0.972 |
| CREM | 0.99 | 0.908 | 0.98 | 0.942 | 0.999 | 0.986 |
| CTCFL | 0.989 | 0.885 | 0.969 | 0.925 | 0.997 | 0.974 |
| DUX4 | 0.997 | 0.948 | 0.994 | 0.97 | 1.0 | 0.996 |
| E2F1 | 0.977 | 0.734 | 0.915 | 0.814 | 0.988 | 0.909 |
| E2F4 | 0.988 | 0.844 | 0.96 | 0.898 | 0.995 | 0.951 |
| E2F6 | 0.989 | 0.836 | 0.993 | 0.908 | 0.999 | 0.976 |
| EBF1 | 0.992 | 0.897 | 0.971 | 0.932 | 0.997 | 0.98 |
| EBF3 | 0.992 | 0.908 | 0.979 | 0.942 | 0.999 | 0.984 |
| EGR1 | 0.971 | 0.798 | 0.788 | 0.793 | 0.984 | 0.887 |
| EHF | 0.997 | 0.94 | 0.995 | 0.967 | 0.999 | 0.995 |
| ELF1 | 0.988 | 0.856 | 0.984 | 0.916 | 0.998 | 0.976 |
| ELF3 | 0.989 | 0.885 | 0.963 | 0.923 | 0.997 | 0.971 |
| ELK4 | 0.989 | 0.862 | 0.946 | 0.902 | 0.998 | 0.969 |
| ESR2 | 0.988 | 0.906 | 0.943 | 0.924 | 0.998 | 0.98 |
| ETV1 | 0.985 | 0.836 | 0.981 | 0.903 | 0.998 | 0.969 |
| FLI1 | 0.987 | 0.859 | 0.921 | 0.889 | 0.997 | 0.96 |
| FOS | 0.995 | 0.93 | 0.991 | 0.96 | 0.999 | 0.992 |
| FOSL1 | 0.993 | 0.902 | 0.98 | 0.94 | 0.999 | 0.986 |
| FOSL2 | 0.991 | 0.868 | 0.984 | 0.923 | 0.999 | 0.982 |
| FOXA1 | 0.991 | 0.904 | 0.978 | 0.939 | 0.999 | 0.987 |
| FOXA2 | 0.986 | 0.89 | 0.848 | 0.869 | 0.995 | 0.93 |
| FOXA3 | 0.985 | 0.805 | 0.955 | 0.874 | 0.995 | 0.942 |
| FOXH1 | 0.997 | 0.953 | 0.991 | 0.972 | 1.0 | 0.995 |
| FOXK1 | 0.987 | 0.886 | 0.933 | 0.909 | 0.994 | 0.949 |
| FOXK2 | 0.986 | 0.834 | 0.936 | 0.882 | 0.997 | 0.951 |
| FOXP2 | 0.995 | 0.915 | 1.0 | 0.956 | 0.999 | 0.981 |
| GABPA | 0.99 | 0.923 | 0.941 | 0.932 | 0.998 | 0.979 |
| GATA1 | 0.988 | 0.837 | 0.981 | 0.903 | 0.998 | 0.97 |
| GATA2 | 0.995 | 0.935 | 0.996 | 0.965 | 0.999 | 0.991 |
| GATA3 | 0.997 | 0.935 | 0.987 | 0.96 | 0.999 | 0.984 |
| GATA4 | 0.987 | 0.843 | 0.967 | 0.901 | 0.997 | 0.961 |
| GATA6 | 0.979 | 0.777 | 0.958 | 0.858 | 0.994 | 0.918 |
| GRHL2 | 0.98 | 0.794 | 0.995 | 0.883 | 0.998 | 0.964 |
| HAND2 | 0.993 | 0.914 | 0.945 | 0.929 | 0.998 | 0.976 |
| HIF1A | 0.988 | 0.842 | 0.926 | 0.882 | 0.988 | 0.932 |
| HLF | 0.992 | 0.903 | 0.989 | 0.944 | 0.999 | 0.986 |
| HNF4A | 0.991 | 0.907 | 0.984 | 0.944 | 0.999 | 0.991 |
| HNF4G | 0.991 | 0.91 | 0.979 | 0.943 | 0.998 | 0.982 |
| HOXB13 | 0.987 | 0.877 | 0.947 | 0.911 | 0.997 | 0.966 |
| IKZF1 | 0.981 | 0.785 | 0.937 | 0.854 | 0.994 | 0.921 |
| IRF1 | 0.975 | 0.849 | 0.93 | 0.888 | 0.993 | 0.962 |
| JUN | 0.994 | 0.923 | 0.997 | 0.958 | 1.0 | 0.996 |
| JUNB | 0.995 | 0.916 | 0.995 | 0.954 | 1.0 | 0.992 |
| JUND | 0.997 | 0.963 | 0.99 | 0.976 | 1.0 | 0.997 |
| KLF15 | 0.972 | 0.682 | 0.916 | 0.782 | 0.986 | 0.868 |
| KLF4 | 0.977 | 0.762 | 0.845 | 0.801 | 0.985 | 0.876 |
| KLF5 | 0.975 | 0.81 | 0.648 | 0.72 | 0.978 | 0.816 |
| KLF9 | 0.978 | 0.814 | 0.852 | 0.832 | 0.992 | 0.921 |
| LHX2 | 0.99 | 0.872 | 0.957 | 0.912 | 0.995 | 0.936 |
| MAFF | 0.995 | 0.958 | 0.991 | 0.974 | 1.0 | 0.997 |
| MAFGNFE2L1 | 0.993 | 0.934 | 0.982 | 0.957 | 0.999 | 0.992 |
| MAFK | 0.995 | 0.939 | 0.996 | 0.967 | 0.999 | 0.994 |
| MAFNFE2 | 0.994 | 0.931 | 0.996 | 0.963 | 1.0 | 0.997 |
| MAX | 0.991 | 0.862 | 0.985 | 0.919 | 0.999 | 0.977 |
| MAZ | 0.978 | 0.765 | 0.857 | 0.808 | 0.981 | 0.878 |
| MEF2A | 0.996 | 0.95 | 0.998 | 0.973 | 1.0 | 0.993 |
| MEF2C | 0.99 | 0.894 | 0.981 | 0.935 | 0.998 | 0.977 |
| MEIS2 | 0.989 | 0.889 | 0.979 | 0.932 | 0.998 | 0.979 |
| MITF | 0.994 | 0.954 | 0.982 | 0.968 | 0.999 | 0.992 |
| MXI1 | 0.988 | 0.873 | 0.957 | 0.913 | 0.997 | 0.962 |
| MYB | 0.992 | 0.878 | 0.984 | 0.928 | 0.998 | 0.949 |
| MYC | 0.983 | 0.787 | 0.987 | 0.876 | 0.996 | 0.935 |
| MYCN | 0.99 | 0.883 | 0.966 | 0.922 | 0.998 | 0.978 |
| MYF5 | 0.986 | 0.883 | 0.89 | 0.886 | 0.994 | 0.924 |
| MYOD1 | 0.985 | 0.817 | 0.987 | 0.894 | 0.997 | 0.963 |
| MYOG | 0.979 | 0.771 | 0.931 | 0.844 | 0.993 | 0.916 |
| MZF1 | 0.981 | 0.823 | 0.911 | 0.865 | 0.991 | 0.916 |
| NEUROD1 | 0.979 | 0.789 | 0.914 | 0.847 | 0.991 | 0.917 |
| NEUROG2 | 0.985 | 0.881 | 0.882 | 0.882 | 0.992 | 0.942 |
| NFIB | 0.996 | 0.964 | 0.999 | 0.981 | 1.0 | 0.999 |
| NFIC | 0.987 | 0.824 | 0.98 | 0.895 | 0.997 | 0.949 |
| NFIL3 | 0.988 | 0.858 | 0.972 | 0.912 | 0.998 | 0.975 |
| NFKB1 | 0.987 | 0.817 | 0.991 | 0.896 | 0.997 | 0.936 |
| NFYA | 0.928 | 0.594 | 0.647 | 0.619 | 0.956 | 0.693 |
| NFYB | 0.943 | 0.574 | 0.91 | 0.704 | 0.978 | 0.789 |
| NFYC | 0.976 | 0.698 | 0.996 | 0.821 | 0.996 | 0.925 |
| NKX2-2 | 0.99 | 0.902 | 0.964 | 0.932 | 0.998 | 0.969 |
| NKX2-5 | 0.987 | 0.829 | 0.953 | 0.886 | 0.993 | 0.936 |
| NR1H4 | 0.985 | 0.832 | 0.915 | 0.872 | 0.984 | 0.909 |
| NR2F2 | 0.980 | 0.762 | 0.931 | 0.838 | 0.992 | 0.897 |
| NR4A1 | 0.991 | 0.866 | 0.97 | 0.915 | 0.996 | 0.948 |
| NRF1 | 0.98 | 0.771 | 0.896 | 0.829 | 0.992 | 0.926 |
| ONECUT1 | 0.984 | 0.901 | 0.9 | 0.901 | 0.996 | 0.968 |
| OSR2 | 0.987 | 0.869 | 0.924 | 0.896 | 0.996 | 0.954 |
| OTX2 | 0.987 | 0.832 | 0.986 | 0.903 | 0.996 | 0.932 |
| PAX5 | 0.983 | 0.846 | 1.0 | 0.916 | 0.999 | 0.992 |
| PBX2 | 0.985 | 0.807 | 0.98 | 0.885 | 0.997 | 0.958 |
| PBX3 | 0.988 | 0.894 | 0.981 | 0.935 | 0.998 | 0.981 |
| PHOX2B | 0.98 | 0.806 | 0.984 | 0.886 | 0.996 | 0.959 |
| PKNOX1 | 0.989 | 0.902 | 0.964 | 0.932 | 0.997 | 0.978 |
| POU2F2 | 0.994 | 0.929 | 0.98 | 0.954 | 0.999 | 0.993 |
| POU2F3 | 0.983 | 0.84 | 0.919 | 0.878 | 0.992 | 0.943 |
| POU5F1 | 0.982 | 0.8 | 0.903 | 0.848 | 0.987 | 0.906 |
| PRDM1 | 0.973 | 0.746 | 0.98 | 0.847 | 0.995 | 0.937 |
| RBPJ | 0.991 | 0.868 | 0.958 | 0.911 | 0.996 | 0.96 |
| RELB | 0.988 | 0.855 | 0.938 | 0.894 | 0.993 | 0.936 |
| RFX1 | 0.988 | 0.914 | 0.915 | 0.914 | 0.996 | 0.968 |
| RFX2 | 0.985 | 0.881 | 0.977 | 0.926 | 0.998 | 0.984 |
| RFX5 | 0.991 | 0.9 | 0.989 | 0.942 | 0.999 | 0.982 |
| RUNX2 | 0.963 | 0.708 | 0.862 | 0.777 | 0.985 | 0.87 |
| RUNX3 | 0.989 | 0.861 | 0.967 | 0.911 | 0.997 | 0.963 |
| SCRT1 | 0.991 | 0.92 | 0.973 | 0.946 | 0.998 | 0.98 |
| SCRT2 | 0.992 | 0.937 | 0.97 | 0.953 | 0.999 | 0.988 |
| SIX1 | 0.984 | 0.789 | 0.959 | 0.866 | 0.994 | 0.948 |
| SIX2 | 0.99 | 0.915 | 0.97 | 0.942 | 0.998 | 0.983 |
| SNAI2 | 0.991 | 0.898 | 0.966 | 0.931 | 0.998 | 0.981 |
| SOX10 | 0.987 | 0.841 | 0.949 | 0.892 | 0.993 | 0.955 |
| SOX13 | 0.986 | 0.808 | 0.969 | 0.881 | 0.996 | 0.946 |
| SOX2 | 0.986 | 0.825 | 0.946 | 0.881 | 0.995 | 0.935 |
| SP1 | 0.957 | 0.577 | 0.804 | 0.672 | 0.976 | 0.743 |
| SP2 | 0.972 | 0.758 | 0.925 | 0.833 | 0.991 | 0.903 |
| SPIB | 0.985 | 0.883 | 0.943 | 0.912 | 0.997 | 0.971 |
| SRF | 0.988 | 0.904 | 0.975 | 0.938 | 0.998 | 0.983 |
| STAT1 | 0.994 | 0.912 | 0.984 | 0.946 | 0.999 | 0.987 |
| STAT1STAT2 | 0.97 | 0.772 | 0.851 | 0.81 | 0.987 | 0.888 |
| STAT3 | 0.99 | 0.855 | 0.99 | 0.918 | 0.998 | 0.968 |
| TCF12 | 0.983 | 0.792 | 0.946 | 0.862 | 0.994 | 0.931 |
| TCF3 | 0.985 | 0.834 | 0.917 | 0.873 | 0.994 | 0.933 |
| TCF4 | 0.982 | 0.799 | 0.957 | 0.871 | 0.996 | 0.948 |
| TCF7 | 0.987 | 0.849 | 0.934 | 0.889 | 0.992 | 0.934 |
| TCF7L2 | 0.993 | 0.913 | 0.997 | 0.953 | 0.999 | 0.991 |
| TEAD1 | 0.991 | 0.888 | 0.984 | 0.934 | 0.998 | 0.977 |
| TEAD2 | 0.976 | 0.766 | 0.909 | 0.831 | 0.985 | 0.903 |
| TEAD4 | 0.990 | 0.880 | 0.968 | 0.922 | 0.998 | 0.973 |
| TFAP2A | 0.990 | 0.892 | 0.992 | 0.939 | 0.999 | 0.990 |
| TFAP2C | 0.989 | 0.888 | 0.970 | 0.928 | 0.998 | 0.978 |
| TFDP1 | 0.978 | 0.738 | 0.922 | 0.820 | 0.989 | 0.884 |
| TP53 | 0.946 | 0.583 | 0.996 | 0.736 | 0.992 | 0.881 |
| TP63 | 0.985 | 0.878 | 0.984 | 0.928 | 0.999 | 0.988 |
| TWIST1 | 0.985 | 0.820 | 0.994 | 0.899 | 0.998 | 0.969 |
| USF1 | 0.995 | 0.920 | 0.994 | 0.956 | 1.000 | 0.992 |
| USF2 | 0.993 | 0.898 | 0.993 | 0.943 | 0.999 | 0.989 |
| YY1 | 0.984 | 0.827 | 0.936 | 0.878 | 0.996 | 0.936 |
| YY2 | 0.987 | 0.899 | 0.870 | 0.884 | 0.993 | 0.942 |
| ZBTB12 | 0.986 | 0.812 | 0.963 | 0.881 | 0.996 | 0.942 |
| ZBTB14 | 0.983 | 0.840 | 0.886 | 0.862 | 0.992 | 0.932 |
| ZBTB26 | 0.970 | 0.759 | 0.872 | 0.812 | 0.989 | 0.873 |
| ZBTB33 | 0.986 | 0.862 | 0.966 | 0.911 | 0.997 | 0.964 |
| ZBTB6 | 0.977 | 0.744 | 0.979 | 0.845 | 0.995 | 0.935 |
| ZBTB7A | 0.983 | 0.810 | 0.973 | 0.884 | 0.996 | 0.949 |
| ZEB1 | 0.978 | 0.799 | 0.690 | 0.741 | 0.983 | 0.849 |
| ZFP42 | 0.986 | 0.893 | 0.989 | 0.938 | 0.998 | 0.982 |
| ZFP57 | 0.980 | 0.793 | 0.930 | 0.856 | 0.995 | 0.932 |
| ZIM3 | 0.984 | 0.842 | 0.967 | 0.900 | 0.996 | 0.962 |
| ZKSCAN1 | 0.992 | 0.897 | 0.957 | 0.926 | 0.998 | 0.979 |
| ZKSCAN5 | 0.976 | 0.780 | 0.917 | 0.843 | 0.993 | 0.932 |
| ZNF135 | 0.987 | 0.891 | 0.925 | 0.908 | 0.992 | 0.952 |
| ZNF136 | 0.997 | 0.974 | 0.982 | 0.978 | 0.999 | 0.996 |
| ZNF140 | 0.996 | 0.966 | 0.995 | 0.980 | 1.000 | 0.998 |
| ZNF148 | 0.972 | 0.789 | 0.734 | 0.760 | 0.979 | 0.850 |
| ZNF16 | 0.994 | 0.962 | 0.987 | 0.974 | 1.000 | 0.997 |
| ZNF189 | 0.981 | 0.799 | 0.955 | 0.870 | 0.996 | 0.943 |
| ZNF24 | 0.948 | 0.720 | 0.331 | 0.454 | 0.969 | 0.655 |
| ZNF263 | 0.899 | 0.522 | 0.398 | 0.452 | 0.903 | 0.524 |
| ZNF317 | 0.982 | 0.823 | 0.893 | 0.857 | 0.995 | 0.950 |
| ZNF331 | 0.975 | 0.742 | 0.981 | 0.845 | 0.996 | 0.952 |
| ZNF341 | 0.988 | 0.857 | 0.957 | 0.904 | 0.997 | 0.954 |
| ZNF382 | 0.992 | 0.964 | 0.967 | 0.965 | 0.999 | 0.995 |
| ZNF384 | 0.977 | 0.781 | 0.859 | 0.818 | 0.985 | 0.894 |
| ZNF449 | 0.988 | 0.874 | 0.976 | 0.922 | 0.998 | 0.975 |
| ZNF460 | 0.966 | 0.782 | 0.794 | 0.788 | 0.981 | 0.882 |
| ZNF528 | 0.996 | 0.968 | 0.984 | 0.976 | 1.000 | 0.998 |
| ZNF549 | 0.962 | 0.618 | 0.970 | 0.755 | 0.988 | 0.879 |
| ZNF610 | 0.965 | 0.711 | 0.844 | 0.772 | 0.987 | 0.862 |
| ZNF652 | 0.993 | 0.909 | 0.978 | 0.942 | 0.998 | 0.985 |
| ZNF680 | 0.992 | 0.925 | 0.973 | 0.948 | 0.999 | 0.988 |
| ZNF682 | 0.981 | 0.809 | 0.997 | 0.893 | 1.0 | 0.993 |
| ZNF707 | 0.992 | 0.918 | 0.98 | 0.948 | 0.998 | 0.989 |
| ZNF708 | 0.987 | 0.869 | 0.966 | 0.915 | 0.997 | 0.976 |
| ZNF8 | 0.992 | 0.931 | 0.995 | 0.962 | 0.999 | 0.995 |
| ZNF816 | 0.98 | 0.848 | 0.941 | 0.892 | 0.992 | 0.955 |
| ZNF85 | 0.994 | 0.946 | 0.982 | 0.964 | 0.999 | 0.994 |
| ZNF93 | 0.982 | 0.857 | 0.932 | 0.893 | 0.997 | 0.971 |

**Supplementary Table S5. The performance evaluation of D-AEDNet on the 188 transcription factor binding site datasets.**

| TFs | Acc | Pre | Rec | F1-S | AUC | AUPR |
| --- | --- | --- | --- | --- | --- | --- |
| AR | 0.987 | 0.852 | 0.999 | 0.92 | 0.998 | 0.962 |
| ASCL1 | 0.982 | 0.842 | 0.885 | 0.863 | 0.994 | 0.872 |
| ATF2 | 0.995 | 0.952 | 0.978 | 0.965 | 0.999 | 0.983 |
| ATF4 | 0.992 | 0.947 | 0.937 | 0.942 | 0.999 | 0.978 |
| BACH2 | 0.983 | 0.857 | 0.909 | 0.882 | 0.99 | 0.915 |
| BATF | 0.988 | 0.844 | 0.967 | 0.901 | 0.997 | 0.921 |
| BATF3 | 0.986 | 0.809 | 0.967 | 0.881 | 0.996 | 0.89 |
| BATFJUN | 0.992 | 0.885 | 0.989 | 0.934 | 0.999 | 0.967 |
| BHLHE22 | 0.983 | 0.766 | 0.938 | 0.843 | 0.994 | 0.843 |
| CDX2 | 0.989 | 0.854 | 0.964 | 0.906 | 0.995 | 0.91 |
| CEBPA | 0.994 | 0.901 | 0.999 | 0.947 | 0.999 | 0.966 |
| CEBPB | 0.997 | 0.958 | 0.995 | 0.977 | 0.999 | 0.988 |
| CEBPD | 0.989 | 0.878 | 0.97 | 0.922 | 0.998 | 0.949 |
| CEBPG | 0.99 | 0.915 | 0.958 | 0.936 | 0.998 | 0.967 |
| CREB1 | 0.989 | 0.86 | 0.971 | 0.912 | 0.997 | 0.914 |
| CREM | 0.989 | 0.896 | 0.974 | 0.933 | 0.997 | 0.95 |
| CTCFL | 0.986 | 0.864 | 0.952 | 0.906 | 0.997 | 0.949 |
| DUX4 | 0.997 | 0.96 | 0.988 | 0.974 | 0.999 | 0.986 |
| E2F1 | 0.975 | 0.707 | 0.923 | 0.801 | 0.988 | 0.765 |
| E2F4 | 0.985 | 0.795 | 0.971 | 0.874 | 0.995 | 0.88 |
| E2F6 | 0.978 | 0.717 | 0.997 | 0.834 | 0.995 | 0.886 |
| EBF1 | 0.991 | 0.874 | 0.979 | 0.924 | 0.998 | 0.959 |
| EBF3 | 0.991 | 0.907 | 0.96 | 0.933 | 0.998 | 0.964 |
| EGR1 | 0.96 | 0.665 | 0.865 | 0.752 | 0.986 | 0.803 |
| EHF | 0.996 | 0.915 | 0.993 | 0.952 | 0.998 | 0.932 |
| ELF1 | 0.987 | 0.838 | 0.999 | 0.912 | 0.998 | 0.948 |
| ELF3 | 0.988 | 0.869 | 0.969 | 0.917 | 0.997 | 0.941 |
| ELK4 | 0.985 | 0.78 | 0.999 | 0.876 | 0.995 | 0.877 |
| ESR2 | 0.979 | 0.788 | 0.992 | 0.878 | 0.996 | 0.921 |
| ETV1 | 0.988 | 0.886 | 0.958 | 0.921 | 0.997 | 0.94 |
| FLI1 | 0.974 | 0.685 | 0.992 | 0.81 | 0.992 | 0.806 |
| FOS | 0.994 | 0.908 | 0.988 | 0.947 | 0.998 | 0.957 |
| FOSL1 | 0.992 | 0.875 | 0.995 | 0.931 | 0.998 | 0.953 |
| FOSL2 | 0.99 | 0.858 | 0.976 | 0.913 | 0.998 | 0.943 |
| FOXA1 | 0.984 | 0.824 | 0.998 | 0.903 | 0.997 | 0.942 |
| FOXA2 | 0.987 | 0.833 | 0.954 | 0.89 | 0.997 | 0.923 |
| FOXA3 | 0.985 | 0.827 | 0.91 | 0.867 | 0.996 | 0.91 |
| FOXH1 | 0.995 | 0.922 | 0.998 | 0.959 | 0.999 | 0.971 |
| FOXK1 | 0.981 | 0.803 | 0.962 | 0.875 | 0.991 | 0.902 |
| FOXK2 | 0.985 | 0.851 | 0.89 | 0.87 | 0.996 | 0.913 |
| FOXP2 | 0.989 | 0.944 | 0.856 | 0.898 | 0.997 | 0.942 |
| GABPA | 0.988 | 0.874 | 0.966 | 0.918 | 0.997 | 0.933 |
| GATA1 | 0.988 | 0.846 | 0.947 | 0.893 | 0.997 | 0.927 |
| GATA2 | 0.992 | 0.898 | 0.999 | 0.946 | 0.999 | 0.971 |
| GATA3 | 0.996 | 0.902 | 0.998 | 0.948 | 0.998 | 0.907 |
| GATA4 | 0.988 | 0.843 | 0.974 | 0.904 | 0.996 | 0.924 |
| GATA6 | 0.985 | 0.851 | 0.923 | 0.886 | 0.996 | 0.927 |
| GRHL2 | 0.988 | 0.879 | 0.969 | 0.922 | 0.997 | 0.946 |
| HAND2 | 0.991 | 0.864 | 0.983 | 0.92 | 0.998 | 0.928 |
| HIF1A | 0.984 | 0.796 | 0.914 | 0.851 | 0.993 | 0.839 |
| HLF | 0.99 | 0.893 | 0.974 | 0.932 | 0.998 | 0.969 |
| HNF4A | 0.99 | 0.901 | 0.977 | 0.937 | 0.998 | 0.969 |
| HNF4G | 0.988 | 0.876 | 0.983 | 0.927 | 0.998 | 0.964 |
| HOXB13 | 0.987 | 0.875 | 0.956 | 0.914 | 0.997 | 0.941 |
| IKZF1 | 0.98 | 0.785 | 0.922 | 0.848 | 0.994 | 0.868 |
| IRF1 | 0.971 | 0.819 | 0.93 | 0.871 | 0.988 | 0.893 |
| JUN | 0.996 | 0.945 | 0.989 | 0.966 | 0.999 | 0.986 |
| JUNB | 0.994 | 0.908 | 0.987 | 0.946 | 0.998 | 0.966 |
| JUND | 0.997 | 0.957 | 0.996 | 0.976 | 1.0 | 0.99 |
| KLF15 | 0.972 | 0.693 | 0.869 | 0.771 | 0.99 | 0.786 |
| KLF4 | 0.97 | 0.681 | 0.872 | 0.765 | 0.982 | 0.726 |
| KLF5 | 0.97 | 0.671 | 0.787 | 0.724 | 0.988 | 0.744 |
| KLF9 | 0.971 | 0.765 | 0.793 | 0.779 | 0.989 | 0.837 |
| LHX2 | 0.986 | 0.814 | 0.967 | 0.884 | 0.996 | 0.895 |
| MAFF | 0.995 | 0.95 | 0.997 | 0.973 | 1.0 | 0.992 |
| MAFGNFE2L1 | 0.991 | 0.946 | 0.94 | 0.943 | 0.998 | 0.979 |
| MAFK | 0.997 | 0.965 | 0.993 | 0.979 | 1.0 | 0.993 |
| MAFNFE2 | 0.992 | 0.942 | 0.945 | 0.944 | 0.995 | 0.946 |
| MAX | 0.991 | 0.854 | 0.985 | 0.915 | 0.998 | 0.94 |
| MAZ | 0.976 | 0.721 | 0.908 | 0.804 | 0.992 | 0.826 |
| MEF2A | 0.986 | 0.847 | 0.996 | 0.916 | 0.999 | 0.977 |
| MEF2C | 0.989 | 0.883 | 0.979 | 0.929 | 0.998 | 0.952 |
| MEIS2 | 0.986 | 0.885 | 0.938 | 0.91 | 0.997 | 0.949 |
| MITF | 0.994 | 0.964 | 0.973 | 0.968 | 0.999 | 0.988 |
| MXI1 | 0.987 | 0.875 | 0.931 | 0.902 | 0.996 | 0.918 |
| MYB | 0.99 | 0.841 | 0.978 | 0.904 | 0.994 | 0.887 |
| MYC | 0.984 | 0.825 | 0.929 | 0.874 | 0.994 | 0.869 |
| MYCN | 0.987 | 0.822 | 0.997 | 0.901 | 0.996 | 0.91 |
| MYF5 | 0.983 | 0.82 | 0.925 | 0.869 | 0.994 | 0.866 |
| MYOD1 | 0.99 | 0.902 | 0.944 | 0.923 | 0.998 | 0.95 |
| MYOG | 0.98 | 0.785 | 0.921 | 0.848 | 0.994 | 0.873 |
| MZF1 | 0.978 | 0.772 | 0.948 | 0.851 | 0.994 | 0.887 |
| NEUROD1 | 0.968 | 0.689 | 0.939 | 0.795 | 0.989 | 0.805 |
| NEUROG2 | 0.985 | 0.862 | 0.923 | 0.891 | 0.996 | 0.918 |
| NFIB | 0.996 | 0.98 | 0.979 | 0.98 | 0.998 | 0.994 |
| NFIC | 0.992 | 0.885 | 0.974 | 0.927 | 0.998 | 0.936 |
| NFIL3 | 0.988 | 0.856 | 0.972 | 0.911 | 0.997 | 0.947 |
| NFKB1 | 0.987 | 0.813 | 0.992 | 0.894 | 0.996 | 0.905 |
| NFYA | 0.911 | 0.505 | 0.51 | 0.507 | 0.947 | 0.506 |
| NFYB | 0.94 | 0.572 | 0.785 | 0.662 | 0.973 | 0.669 |
| NFYC | 0.983 | 0.779 | 0.961 | 0.861 | 0.995 | 0.872 |
| NKX2-2 | 0.989 | 0.874 | 0.979 | 0.923 | 0.997 | 0.931 |
| NKX2-5 | 0.978 | 0.845 | 0.729 | 0.783 | 0.993 | 0.868 |
| NR1H4 | 0.981 | 0.854 | 0.792 | 0.822 | 0.987 | 0.861 |
| NR2F2 | 0.98 | 0.798 | 0.849 | 0.823 | 0.993 | 0.865 |
| NR4A1 | 0.992 | 0.889 | 0.971 | 0.928 | 0.998 | 0.951 |
| NRF1 | 0.971 | 0.675 | 0.912 | 0.776 | 0.99 | 0.804 |
| ONECUT1 | 0.982 | 0.874 | 0.91 | 0.892 | 0.996 | 0.945 |
| OSR2 | 0.984 | 0.838 | 0.913 | 0.874 | 0.995 | 0.884 |
| OTX2 | 0.988 | 0.859 | 0.958 | 0.906 | 0.997 | 0.927 |
| PAX5 | 0.99 | 0.909 | 0.991 | 0.948 | 0.999 | 0.986 |
| PBX2 | 0.981 | 0.795 | 0.925 | 0.855 | 0.994 | 0.9 |
| PBX3 | 0.989 | 0.896 | 0.985 | 0.938 | 0.997 | 0.96 |
| PHOX2B | 0.987 | 0.878 | 0.974 | 0.923 | 0.997 | 0.951 |
| PKNOX1 | 0.988 | 0.874 | 0.979 | 0.924 | 0.998 | 0.96 |
| POU2F2 | 0.994 | 0.923 | 0.982 | 0.952 | 0.999 | 0.98 |
| POU2F3 | 0.985 | 0.832 | 0.957 | 0.89 | 0.996 | 0.925 |
| POU5F1 | 0.982 | 0.782 | 0.932 | 0.851 | 0.994 | 0.871 |
| PRDM1 | 0.983 | 0.828 | 0.97 | 0.893 | 0.997 | 0.962 |
| RBPJ | 0.988 | 0.859 | 0.91 | 0.884 | 0.996 | 0.902 |
| RELB | 0.981 | 0.81 | 0.866 | 0.837 | 0.994 | 0.879 |
| RFX1 | 0.989 | 0.899 | 0.943 | 0.92 | 0.998 | 0.955 |
| RFX2 | 0.968 | 0.752 | 0.99 | 0.855 | 0.994 | 0.926 |
| RFX5 | 0.991 | 0.896 | 0.997 | 0.943 | 0.998 | 0.964 |
| RUNX2 | 0.946 | 0.691 | 0.5 | 0.58 | 0.948 | 0.59 |
| RUNX3 | 0.987 | 0.848 | 0.961 | 0.901 | 0.996 | 0.918 |
| SCRT1 | 0.993 | 0.928 | 0.989 | 0.958 | 0.999 | 0.981 |
| SCRT2 | 0.991 | 0.913 | 0.988 | 0.949 | 0.999 | 0.975 |
| SIX1 | 0.98 | 0.84 | 0.797 | 0.818 | 0.99 | 0.887 |
| SIX2 | 0.986 | 0.87 | 0.964 | 0.914 | 0.997 | 0.953 |
| SNAI2 | 0.991 | 0.904 | 0.971 | 0.936 | 0.998 | 0.971 |
| SOX10 | 0.982 | 0.801 | 0.904 | 0.849 | 0.992 | 0.871 |
| SOX13 | 0.98 | 0.757 | 0.925 | 0.833 | 0.992 | 0.806 |
| SOX2 | 0.989 | 0.863 | 0.942 | 0.901 | 0.997 | 0.914 |
| SP1 | 0.951 | 0.538 | 0.814 | 0.647 | 0.978 | 0.64 |
| SP2 | 0.954 | 0.629 | 0.928 | 0.75 | 0.982 | 0.753 |
| SPIB | 0.983 | 0.84 | 0.968 | 0.899 | 0.996 | 0.945 |
| SRF | 0.984 | 0.858 | 0.99 | 0.92 | 0.996 | 0.932 |
| STAT1 | 0.992 | 0.873 | 0.994 | 0.929 | 0.998 | 0.946 |
| STAT1STAT2 | 0.966 | 0.747 | 0.832 | 0.787 | 0.963 | 0.744 |
| STAT3 | 0.993 | 0.891 | 0.997 | 0.941 | 0.998 | 0.932 |
| TCF12 | 0.985 | 0.817 | 0.941 | 0.875 | 0.995 | 0.891 |
| TCF3 | 0.986 | 0.808 | 0.97 | 0.881 | 0.996 | 0.902 |
| TCF4 | 0.981 | 0.819 | 0.918 | 0.866 | 0.995 | 0.893 |
| TCF7 | 0.987 | 0.827 | 0.963 | 0.89 | 0.996 | 0.919 |
| TCF7L2 | 0.988 | 0.853 | 1.0 | 0.92 | 0.999 | 0.985 |
| TEAD1 | 0.993 | 0.919 | 0.982 | 0.95 | 0.999 | 0.97 |
| TEAD2 | 0.969 | 0.777 | 0.743 | 0.76 | 0.98 | 0.773 |
| TEAD4 | 0.991 | 0.887 | 0.966 | 0.925 | 0.998 | 0.947 |
| TFAP2A | 0.982 | 0.81 | 0.993 | 0.892 | 0.998 | 0.975 |
| TFAP2C | 0.983 | 0.814 | 0.997 | 0.897 | 0.998 | 0.959 |
| TFDP1 | 0.975 | 0.738 | 0.84 | 0.786 | 0.989 | 0.805 |
| TP53 | 0.989 | 0.869 | 0.999 | 0.929 | 0.998 | 0.964 |
| TP63 | 0.983 | 0.867 | 0.984 | 0.922 | 0.998 | 0.971 |
| TWIST1 | 0.989 | 0.877 | 0.974 | 0.923 | 0.997 | 0.943 |
| USF1 | 0.994 | 0.909 | 0.995 | 0.95 | 0.999 | 0.974 |
| USF2 | 0.994 | 0.91 | 0.987 | 0.947 | 0.999 | 0.966 |
| YY1 | 0.987 | 0.83 | 0.991 | 0.903 | 0.997 | 0.946 |
| YY2 | 0.982 | 0.824 | 0.867 | 0.845 | 0.995 | 0.888 |
| ZBTB12 | 0.981 | 0.779 | 0.914 | 0.841 | 0.994 | 0.864 |
| ZBTB14 | 0.974 | 0.787 | 0.769 | 0.778 | 0.99 | 0.836 |
| ZBTB26 | 0.957 | 0.645 | 0.945 | 0.767 | 0.981 | 0.69 |
| ZBTB33 | 0.968 | 0.708 | 0.984 | 0.824 | 0.992 | 0.867 |
| ZBTB6 | 0.981 | 0.79 | 0.956 | 0.865 | 0.994 | 0.887 |
| ZBTB7A | 0.987 | 0.872 | 0.944 | 0.907 | 0.996 | 0.927 |
| ZEB1 | 0.969 | 0.636 | 0.731 | 0.68 | 0.982 | 0.71 |
| ZFP42 | 0.983 | 0.882 | 0.973 | 0.925 | 0.995 | 0.946 |
| ZFP57 | 0.974 | 0.738 | 0.937 | 0.826 | 0.991 | 0.825 |
| ZIM3 | 0.989 | 0.89 | 0.97 | 0.928 | 0.998 | 0.962 |
| ZKSCAN1 | 0.987 | 0.802 | 0.976 | 0.881 | 0.997 | 0.909 |
| ZKSCAN5 | 0.966 | 0.761 | 0.752 | 0.756 | 0.987 | 0.795 |
| ZNF135 | 0.98 | 0.844 | 0.87 | 0.857 | 0.978 | 0.861 |
| ZNF136 | 0.996 | 0.971 | 0.978 | 0.975 | 0.997 | 0.982 |
| ZNF140 | 0.992 | 0.945 | 0.985 | 0.965 | 0.996 | 0.988 |
| ZNF148 | 0.961 | 0.734 | 0.56 | 0.635 | 0.982 | 0.753 |
| ZNF16 | 0.982 | 0.936 | 0.903 | 0.919 | 0.993 | 0.969 |
| ZNF189 | 0.983 | 0.803 | 0.973 | 0.88 | 0.995 | 0.904 |
| ZNF24 | 0.945 | 0.734 | 0.246 | 0.368 | 0.968 | 0.629 |
| ZNF263 | 0.869 | 0.414 | 0.6 | 0.49 | 0.896 | 0.497 |
| ZNF317 | 0.979 | 0.784 | 0.907 | 0.841 | 0.995 | 0.904 |
| ZNF331 | 0.978 | 0.807 | 0.893 | 0.847 | 0.991 | 0.884 |
| ZNF341 | 0.987 | 0.839 | 0.976 | 0.902 | 0.997 | 0.927 |
| ZNF382 | 0.99 | 0.936 | 0.983 | 0.959 | 0.998 | 0.989 |
| ZNF384 | 0.973 | 0.783 | 0.766 | 0.775 | 0.991 | 0.863 |
| ZNF449 | 0.987 | 0.869 | 0.964 | 0.914 | 0.997 | 0.945 |
| ZNF460 | 0.953 | 0.668 | 0.81 | 0.732 | 0.977 | 0.776 |
| ZNF528 | 0.992 | 0.939 | 0.965 | 0.952 | 0.99 | 0.967 |
| ZNF549 | 0.977 | 0.784 | 0.854 | 0.817 | 0.99 | 0.831 |
| ZNF610 | 0.954 | 0.645 | 0.764 | 0.699 | 0.979 | 0.721 |
| ZNF652 | 0.992 | 0.896 | 0.978 | 0.936 | 0.999 | 0.971 |
| ZNF680 | 0.989 | 0.913 | 0.946 | 0.929 | 0.998 | 0.957 |
| ZNF682 | 0.985 | 0.966 | 0.837 | 0.897 | 0.995 | 0.956 |
| ZNF707 | 0.993 | 0.964 | 0.941 | 0.953 | 0.991 | 0.971 |
| ZNF708 | 0.981 | 0.87 | 0.881 | 0.875 | 0.987 | 0.915 |
| ZNF8 | 0.991 | 0.932 | 0.983 | 0.957 | 0.997 | 0.978 |
| ZNF816 | 0.966 | 0.745 | 0.949 | 0.835 | 0.987 | 0.85 |
| ZNF85 | 0.988 | 0.934 | 0.918 | 0.926 | 0.996 | 0.957 |
| ZNF93 | 0.979 | 0.867 | 0.873 | 0.87 | 0.996 | 0.957 |

**Supplementary Table S6. The performance evaluation of DeepSNR on the 188 transcription factor binding site datasets.**

| TFs | Acc | Pre | Rec | F1-S | AUC | AUPR |
| --- | --- | --- | --- | --- | --- | --- |
| AR | 0.987 | 0.87 | 0.98 | 0.922 | 0.989 | 0.973 |
| ASCL1 | 0.96 | 0.634 | 0.926 | 0.753 | 0.956 | 0.834 |
| ATF2 | 0.993 | 0.935 | 0.957 | 0.946 | 0.978 | 0.965 |
| ATF4 | 0.99 | 0.923 | 0.939 | 0.931 | 0.969 | 0.954 |
| BACH2 | 0.977 | 0.762 | 0.97 | 0.854 | 0.983 | 0.948 |
| BATF | 0.984 | 0.83 | 0.883 | 0.855 | 0.939 | 0.878 |
| BATF3 | 0.977 | 0.724 | 0.931 | 0.815 | 0.962 | 0.87 |
| BATFJUN | 0.986 | 0.816 | 0.965 | 0.884 | 0.982 | 0.96 |
| BHLHE22 | 0.978 | 0.719 | 0.92 | 0.807 | 0.955 | 0.834 |
| CDX2 | 0.968 | 0.655 | 0.883 | 0.752 | 0.938 | 0.867 |
| CEBPA | 0.993 | 0.908 | 0.98 | 0.943 | 0.989 | 0.973 |
| CEBPB | 0.996 | 0.946 | 0.99 | 0.968 | 0.995 | 0.986 |
| CEBPD | 0.975 | 0.745 | 0.946 | 0.834 | 0.97 | 0.91 |
| CEBPG | 0.987 | 0.9 | 0.934 | 0.917 | 0.966 | 0.943 |
| CREB1 | 0.984 | 0.819 | 0.94 | 0.875 | 0.968 | 0.907 |
| CREM | 0.984 | 0.855 | 0.96 | 0.904 | 0.977 | 0.927 |
| CTCFL | 0.977 | 0.812 | 0.865 | 0.838 | 0.93 | 0.896 |
| DUX4 | 0.995 | 0.925 | 0.986 | 0.955 | 0.993 | 0.983 |
| E2F1 | 0.613 | 0.088 | 0.648 | 0.156 | 0.662 | 0.128 |
| E2F4 | 0.969 | 0.645 | 0.958 | 0.771 | 0.976 | 0.908 |
| E2F6 | 0.98 | 0.749 | 0.96 | 0.841 | 0.978 | 0.928 |
| EBF1 | 0.99 | 0.87 | 0.966 | 0.916 | 0.982 | 0.959 |
| EBF3 | 0.983 | 0.813 | 0.958 | 0.879 | 0.977 | 0.938 |
| EGR1 | 0.93 | 0.0 | 0.0 | 0.0 | 0.5 | 0.535 |
| EHF | 0.962 | 0.515 | 0.988 | 0.677 | 0.993 | 0.965 |
| ELF1 | 0.99 | 0.885 | 0.977 | 0.929 | 0.988 | 0.97 |
| ELF3 | 0.981 | 0.816 | 0.94 | 0.874 | 0.967 | 0.907 |
| ELK4 | 0.979 | 0.742 | 0.944 | 0.831 | 0.969 | 0.899 |
| ESR2 | 0.981 | 0.823 | 0.953 | 0.883 | 0.974 | 0.935 |
| ETV1 | 0.984 | 0.846 | 0.939 | 0.89 | 0.966 | 0.916 |
| FLI1 | 0.966 | 0.633 | 0.927 | 0.752 | 0.959 | 0.877 |
| FOS | 0.994 | 0.921 | 0.975 | 0.947 | 0.987 | 0.973 |
| FOSL1 | 0.982 | 0.768 | 0.976 | 0.86 | 0.987 | 0.951 |
| FOSL2 | 0.985 | 0.807 | 0.967 | 0.88 | 0.982 | 0.953 |
| FOXA1 | 0.988 | 0.884 | 0.962 | 0.921 | 0.979 | 0.95 |
| FOXA2 | 0.982 | 0.806 | 0.874 | 0.839 | 0.934 | 0.874 |
| FOXA3 | 0.98 | 0.779 | 0.884 | 0.828 | 0.938 | 0.859 |
| FOXH1 | 0.877 | 0.307 | 0.988 | 0.468 | 0.992 | 0.962 |
| FOXK1 | 0.95 | 0.592 | 0.898 | 0.713 | 0.944 | 0.877 |
| FOXK2 | 0.981 | 0.801 | 0.881 | 0.839 | 0.938 | 0.868 |
| FOXP2 | 0.95 | 0.524 | 0.979 | 0.682 | 0.988 | 0.951 |
| GABPA | 0.983 | 0.827 | 0.949 | 0.884 | 0.972 | 0.92 |
| GATA1 | 0.981 | 0.808 | 0.86 | 0.833 | 0.927 | 0.856 |
| GATA2 | 0.977 | 0.756 | 0.998 | 0.86 | 0.998 | 0.988 |
| GATA3 | 0.982 | 0.69 | 0.989 | 0.813 | 0.993 | 0.957 |
| GATA4 | 0.983 | 0.822 | 0.924 | 0.87 | 0.959 | 0.895 |
| GATA6 | 0.979 | 0.806 | 0.902 | 0.851 | 0.948 | 0.89 |
| GRHL2 | 0.966 | 0.698 | 0.964 | 0.81 | 0.979 | 0.929 |
| HAND2 | 0.988 | 0.844 | 0.94 | 0.89 | 0.968 | 0.925 |
| HIF1A | 0.954 | 0.524 | 0.943 | 0.674 | 0.968 | 0.897 |
| HLF | 0.99 | 0.921 | 0.945 | 0.933 | 0.971 | 0.954 |
| HNF4A | 0.988 | 0.882 | 0.974 | 0.926 | 0.986 | 0.964 |
| HNF4G | 0.983 | 0.829 | 0.977 | 0.897 | 0.988 | 0.966 |
| HOXB13 | 0.98 | 0.807 | 0.935 | 0.866 | 0.964 | 0.902 |
| IKZF1 | 0.973 | 0.73 | 0.867 | 0.793 | 0.928 | 0.795 |
| IRF1 | 0.952 | 0.709 | 0.915 | 0.799 | 0.951 | 0.876 |
| JUN | 0.994 | 0.933 | 0.982 | 0.957 | 0.991 | 0.983 |
| JUNB | 0.992 | 0.885 | 0.978 | 0.929 | 0.988 | 0.977 |
| JUND | 0.997 | 0.957 | 0.988 | 0.972 | 0.994 | 0.99 |
| KLF15 | 0.968 | 0.686 | 0.776 | 0.728 | 0.882 | 0.749 |
| KLF4 | 0.932 | 0.441 | 0.879 | 0.587 | 0.929 | 0.76 |
| KLF5 | 0.95 | 0.0 | 0.0 | 0.0 | 0.5 | 0.525 |
| KLF9 | 0.963 | 0.69 | 0.77 | 0.728 | 0.879 | 0.781 |
| LHX2 | 0.976 | 0.794 | 0.773 | 0.783 | 0.883 | 0.81 |
| MAFF | 0.991 | 0.912 | 0.993 | 0.951 | 0.996 | 0.991 |
| MAFGNFE2L1 | 0.974 | 0.758 | 0.985 | 0.857 | 0.992 | 0.98 |
| MAFK | 0.994 | 0.931 | 0.99 | 0.96 | 0.995 | 0.99 |
| MAFNFE2 | 0.96 | 0.655 | 0.981 | 0.786 | 0.989 | 0.975 |
| MAX | 0.989 | 0.85 | 0.956 | 0.9 | 0.977 | 0.945 |
| MAZ | 0.941 | 0.414 | 0.192 | 0.262 | 0.588 | 0.281 |
| MEF2A | 0.847 | 0.329 | 0.995 | 0.494 | 0.996 | 0.979 |
| MEF2C | 0.957 | 0.639 | 0.976 | 0.773 | 0.986 | 0.941 |
| MEIS2 | 0.983 | 0.84 | 0.949 | 0.891 | 0.972 | 0.928 |
| MITF | 0.991 | 0.924 | 0.979 | 0.951 | 0.988 | 0.977 |
| MXI1 | 0.966 | 0.66 | 0.979 | 0.789 | 0.986 | 0.917 |
| MYB | 0.658 | 0.089 | 0.635 | 0.157 | 0.658 | 0.088 |
| MYC | 0.979 | 0.755 | 0.97 | 0.849 | 0.981 | 0.908 |
| MYCN | 0.972 | 0.693 | 0.964 | 0.806 | 0.979 | 0.9 |
| MYF5 | 0.978 | 0.755 | 0.937 | 0.836 | 0.964 | 0.85 |
| MYOD1 | 0.983 | 0.823 | 0.941 | 0.878 | 0.968 | 0.921 |
| MYOG | 0.973 | 0.744 | 0.85 | 0.793 | 0.92 | 0.825 |
| MZF1 | 0.973 | 0.739 | 0.896 | 0.81 | 0.943 | 0.847 |
| NEUROD1 | 0.954 | 0.594 | 0.904 | 0.717 | 0.944 | 0.8 |
| NEUROG2 | 0.98 | 0.803 | 0.912 | 0.854 | 0.953 | 0.891 |
| NFIB | 0.982 | 0.89 | 0.946 | 0.917 | 0.972 | 0.969 |
| NFIC | 0.983 | 0.785 | 0.945 | 0.858 | 0.97 | 0.906 |
| NFIL3 | 0.975 | 0.814 | 0.805 | 0.81 | 0.9 | 0.846 |
| NFKB1 | 0.977 | 0.708 | 0.974 | 0.82 | 0.985 | 0.939 |
| NFYA | 0.879 | 0.345 | 0.379 | 0.361 | 0.659 | 0.343 |
| NFYB | 0.913 | 0.435 | 0.533 | 0.479 | 0.747 | 0.526 |
| NFYC | 0.974 | 0.749 | 0.803 | 0.775 | 0.897 | 0.797 |
| NKX2-2 | 0.983 | 0.829 | 0.951 | 0.886 | 0.973 | 0.907 |
| NKX2-5 | 0.976 | 0.726 | 0.891 | 0.8 | 0.941 | 0.848 |
| NR1H4 | 0.873 | 0.281 | 0.836 | 0.421 | 0.9 | 0.713 |
| NR2F2 | 0.961 | 0.614 | 0.796 | 0.693 | 0.89 | 0.734 |
| NR4A1 | 0.987 | 0.825 | 0.936 | 0.877 | 0.966 | 0.923 |
| NRF1 | 0.966 | 0.66 | 0.806 | 0.725 | 0.898 | 0.789 |
| ONECUT1 | 0.978 | 0.855 | 0.878 | 0.866 | 0.936 | 0.905 |
| OSR2 | 0.978 | 0.756 | 0.929 | 0.834 | 0.96 | 0.872 |
| OTX2 | 0.984 | 0.842 | 0.906 | 0.873 | 0.951 | 0.894 |
| PAX5 | 0.885 | 0.45 | 0.971 | 0.615 | 0.981 | 0.949 |
| PBX2 | 0.959 | 0.615 | 0.827 | 0.706 | 0.909 | 0.831 |
| PBX3 | 0.978 | 0.801 | 0.983 | 0.883 | 0.989 | 0.961 |
| PHOX2B | 0.98 | 0.839 | 0.933 | 0.884 | 0.963 | 0.911 |
| PKNOX1 | 0.984 | 0.846 | 0.96 | 0.899 | 0.978 | 0.941 |
| POU2F2 | 0.978 | 0.788 | 0.902 | 0.841 | 0.949 | 0.916 |
| POU2F3 | 0.98 | 0.816 | 0.895 | 0.854 | 0.944 | 0.891 |
| POU5F1 | 0.976 | 0.739 | 0.859 | 0.794 | 0.925 | 0.825 |
| PRDM1 | 0.9 | 0.427 | 0.963 | 0.592 | 0.977 | 0.918 |
| RBPJ | 0.869 | 0.131 | 0.286 | 0.18 | 0.593 | 0.101 |
| RELB | 0.966 | 0.632 | 0.909 | 0.745 | 0.95 | 0.871 |
| RFX1 | 0.983 | 0.827 | 0.958 | 0.888 | 0.977 | 0.943 |
| RFX2 | 0.972 | 0.798 | 0.949 | 0.867 | 0.972 | 0.95 |
| RFX5 | 0.959 | 0.647 | 0.984 | 0.781 | 0.99 | 0.968 |
| RUNX2 | 0.786 | 0.167 | 0.464 | 0.245 | 0.647 | 0.157 |
| RUNX3 | 0.98 | 0.781 | 0.929 | 0.848 | 0.961 | 0.885 |
| SCRT1 | 0.99 | 0.908 | 0.973 | 0.939 | 0.985 | 0.965 |
| SCRT2 | 0.989 | 0.904 | 0.964 | 0.933 | 0.981 | 0.957 |
| SIX1 | 0.939 | 0.475 | 0.937 | 0.63 | 0.964 | 0.905 |
| SIX2 | 0.959 | 0.671 | 0.968 | 0.792 | 0.981 | 0.945 |
| SNAI2 | 0.988 | 0.88 | 0.948 | 0.913 | 0.973 | 0.951 |
| SOX10 | 0.939 | 0.474 | 0.942 | 0.631 | 0.966 | 0.892 |
| SOX13 | 0.966 | 0.631 | 0.93 | 0.752 | 0.96 | 0.84 |
| SOX2 | 0.981 | 0.774 | 0.917 | 0.839 | 0.954 | 0.854 |
| SP1 | 0.945 | 0.0 | 0.0 | 0.0 | 0.5 | 0.528 |
| SP2 | 0.908 | 0.431 | 0.693 | 0.532 | 0.825 | 0.595 |
| SPIB | 0.979 | 0.825 | 0.931 | 0.875 | 0.963 | 0.918 |
| SRF | 0.926 | 0.551 | 0.972 | 0.703 | 0.983 | 0.946 |
| STAT1 | 0.977 | 0.717 | 0.969 | 0.824 | 0.983 | 0.957 |
| STAT1STAT2 | 0.858 | 0.334 | 0.899 | 0.487 | 0.934 | 0.777 |
| STAT3 | 0.993 | 0.898 | 0.976 | 0.935 | 0.987 | 0.969 |
| TCF12 | 0.975 | 0.724 | 0.87 | 0.79 | 0.93 | 0.814 |
| TCF3 | 0.976 | 0.734 | 0.872 | 0.797 | 0.931 | 0.827 |
| TCF4 | 0.973 | 0.767 | 0.85 | 0.806 | 0.92 | 0.831 |
| TCF7 | 0.98 | 0.762 | 0.917 | 0.832 | 0.955 | 0.877 |
| TCF7L2 | 0.852 | 0.321 | 0.993 | 0.485 | 0.995 | 0.978 |
| TEAD1 | 0.988 | 0.872 | 0.96 | 0.914 | 0.978 | 0.948 |
| TEAD2 | 0.856 | 0.299 | 0.898 | 0.448 | 0.92 | 0.644 |
| TEAD4 | 0.988 | 0.86 | 0.945 | 0.901 | 0.971 | 0.929 |
| TFAP2A | 0.982 | 0.821 | 0.98 | 0.893 | 0.989 | 0.973 |
| TFAP2C | 0.981 | 0.812 | 0.975 | 0.886 | 0.986 | 0.963 |
| TFDP1 | 0.966 | 0.635 | 0.881 | 0.738 | 0.935 | 0.815 |
| TP53 | 0.86 | 0.349 | 0.995 | 0.517 | 0.996 | 0.981 |
| TP63 | 0.973 | 0.811 | 0.951 | 0.875 | 0.973 | 0.956 |
| TWIST1 | 0.984 | 0.83 | 0.957 | 0.889 | 0.975 | 0.914 |
| USF1 | 0.988 | 0.832 | 0.978 | 0.899 | 0.988 | 0.966 |
| USF2 | 0.992 | 0.902 | 0.964 | 0.932 | 0.981 | 0.956 |
| YY1 | 0.987 | 0.861 | 0.938 | 0.898 | 0.968 | 0.948 |
| YY2 | 0.976 | 0.765 | 0.806 | 0.785 | 0.899 | 0.803 |
| ZBTB12 | 0.963 | 0.612 | 0.902 | 0.729 | 0.944 | 0.788 |
| ZBTB14 | 0.935 | 0.432 | 0.289 | 0.346 | 0.633 | 0.343 |
| ZBTB26 | 0.896 | 0.412 | 0.895 | 0.564 | 0.928 | 0.691 |
| ZBTB33 | 0.901 | 0.428 | 0.964 | 0.593 | 0.976 | 0.912 |
| ZBTB6 | 0.944 | 0.54 | 0.95 | 0.688 | 0.969 | 0.87 |
| ZBTB7A | 0.981 | 0.81 | 0.929 | 0.866 | 0.961 | 0.873 |
| ZEB1 | 0.957 | 0.513 | 0.82 | 0.631 | 0.902 | 0.733 |
| ZFP42 | 0.964 | 0.753 | 0.977 | 0.851 | 0.987 | 0.963 |
| ZFP57 | 0.965 | 0.674 | 0.904 | 0.772 | 0.945 | 0.814 |
| ZIM3 | 0.987 | 0.892 | 0.938 | 0.915 | 0.967 | 0.942 |
| ZKSCAN1 | 0.984 | 0.781 | 0.937 | 0.852 | 0.967 | 0.927 |
| ZKSCAN5 | 0.96 | 0.688 | 0.786 | 0.734 | 0.886 | 0.773 |
| ZNF135 | 0.941 | 0.552 | 0.812 | 0.657 | 0.899 | 0.828 |
| ZNF136 | 0.99 | 0.894 | 0.977 | 0.933 | 0.988 | 0.979 |
| ZNF140 | 0.964 | 0.764 | 0.949 | 0.846 | 0.973 | 0.968 |
| ZNF148 | 0.94 | 0.0 | 0.0 | 0.0 | 0.5 | 0.53 |
| ZNF16 | 0.945 | 0.689 | 0.956 | 0.801 | 0.976 | 0.968 |
| ZNF189 | 0.976 | 0.762 | 0.909 | 0.829 | 0.951 | 0.881 |
| ZNF24 | 0.944 | 0.615 | 0.356 | 0.451 | 0.671 | 0.488 |
| ZNF263 | 0.903 | 0.572 | 0.312 | 0.404 | 0.644 | 0.46 |
| ZNF317 | 0.976 | 0.759 | 0.877 | 0.814 | 0.934 | 0.849 |
| ZNF331 | 0.963 | 0.681 | 0.893 | 0.772 | 0.942 | 0.882 |
| ZNF341 | 0.983 | 0.809 | 0.943 | 0.871 | 0.968 | 0.9 |
| ZNF382 | 0.975 | 0.86 | 0.949 | 0.903 | 0.974 | 0.971 |
| ZNF384 | 0.972 | 0.782 | 0.73 | 0.755 | 0.861 | 0.791 |
| ZNF449 | 0.984 | 0.842 | 0.945 | 0.89 | 0.97 | 0.934 |
| ZNF460 | 0.942 | 0.619 | 0.714 | 0.663 | 0.848 | 0.748 |
| ZNF528 | 0.942 | 0.597 | 0.986 | 0.744 | 0.992 | 0.987 |
| ZNF549 | 0.973 | 0.734 | 0.858 | 0.791 | 0.925 | 0.855 |
| ZNF610 | 0.928 | 0.491 | 0.828 | 0.617 | 0.901 | 0.738 |
| ZNF652 | 0.989 | 0.875 | 0.958 | 0.915 | 0.977 | 0.943 |
| ZNF680 | 0.973 | 0.749 | 0.967 | 0.844 | 0.982 | 0.953 |
| ZNF682 | 0.967 | 0.711 | 0.987 | 0.827 | 0.992 | 0.973 |
| ZNF707 | 0.975 | 0.759 | 0.978 | 0.855 | 0.988 | 0.982 |
| ZNF708 | 0.959 | 0.656 | 0.968 | 0.782 | 0.981 | 0.948 |
| ZNF8 | 0.98 | 0.945 | 0.85 | 0.895 | 0.924 | 0.925 |
| ZNF816 | 0.953 | 0.69 | 0.87 | 0.77 | 0.93 | 0.873 |
| ZNF85 | 0.936 | 0.559 | 0.991 | 0.715 | 0.995 | 0.99 |
| ZNF93 | 0.959 | 0.896 | 0.55 | 0.682 | 0.774 | 0.764 |

**Supplementary Table S7. The performance evaluation of Matching on the 188 transcription factor binding site datasets.**

| TFs | Acc | Pre | Rec | F1-S | AUC | AUPR |
| --- | --- | --- | --- | --- | --- | --- |
| AR | 0.964 | 0.841 | 0.641 | 0.727 | **-** | **-** |
| ASCL1 | 0.952 | 0.639 | 0.587 | 0.612 | **-** | **-** |
| ATF2 | 0.988 | 0.93 | 0.874 | 0.901 | **-** | **-** |
| ATF4 | 0.989 | 0.927 | 0.91 | 0.918 | **-** | **-** |
| BACH2 | 0.941 | 0.693 | 0.273 | 0.392 | **-** | **-** |
| BATF | 0.974 | 0.844 | 0.656 | 0.739 | **-** | **-** |
| BATF3 | 0.977 | 0.818 | 0.753 | 0.784 | **-** | **-** |
| BATFJUN | 0.969 | 0.691 | 0.792 | 0.738 | **-** | **-** |
| BHLHE22 | 0.900 | 0.276 | 0.613 | 0.381 | **-** | **-** |
| CDX2 | 0.977 | 0.719 | 0.956 | 0.821 | **-** | **-** |
| CEBPA | 0.977 | 0.709 | 0.984 | 0.824 | **-** | **-** |
| CEBPB | 0.962 | 0.886 | 0.349 | 0.5 | **-** | **-** |
| CEBPD | 0.98 | 0.885 | 0.793 | 0.836 | **-** | **-** |
| CEBPG | 0.984 | 0.900 | 0.879 | 0.890 | **-** | **-** |
| CREB1 | 0.955 | 0.712 | 0.422 | 0.529 | **-** | **-** |
| CREM | 0.97 | 0.895 | 0.705 | 0.789 | **-** | **-** |
| CTCFL | 0.97 | 0.811 | 0.745 | 0.776 | **-** | **-** |
| DUX4 | 0.995 | 0.933 | 0.985 | 0.958 | **-** | **-** |
| E2F1 | 0.976 | 0.696 | 0.991 | 0.817 | **-** | **-** |
| E2F4 | 0.979 | 0.732 | 0.987 | 0.841 | **-** | **-** |
| E2F6 | 0.966 | 0.623 | 0.946 | 0.751 | **-** | **-** |
| EBF1 | 0.96 | 0.604 | 0.819 | 0.695 | **-** | **-** |
| EBF3 | 0.979 | 0.879 | 0.785 | 0.829 | **-** | **-** |
| EGR1 | 0.917 | 0.446 | 0.77 | 0.565 | **-** | **-** |
| EHF | 0.953 | 0.459 | 0.947 | 0.618 | **-** | **-** |
| ELF1 | 0.972 | 0.736 | 0.89 | 0.806 | **-** | **-** |
| ELF3 | 0.972 | 0.837 | 0.754 | 0.793 | **-** | **-** |
| ELK4 | 0.984 | 0.783 | 0.991 | 0.875 | **-** | **-** |
| ESR2 | 0.986 | 0.852 | 0.985 | 0.914 | **-** | **-** |
| ETV1 | 0.977 | 0.861 | 0.805 | 0.832 | **-** | **-** |
| FLI1 | 0.967 | 0.764 | 0.587 | 0.664 | **-** | **-** |
| FOS | 0.993 | 0.881 | 0.999 | 0.936 | **-** | **-** |
| FOSL1 | 0.978 | 0.719 | 0.983 | 0.83 | **-** | **-** |
| FOSL2 | 0.988 | 0.827 | 0.994 | 0.903 | **-** | **-** |
| FOXA1 | 0.967 | 0.808 | 0.727 | 0.766 | **-** | **-** |
| FOXA2 | 0.971 | 0.82 | 0.611 | 0.7 | **-** | **-** |
| FOXA3 | 0.966 | 0.828 | 0.487 | 0.614 | **-** | **-** |
| FOXH1 | 0.996 | 0.938 | 0.999 | 0.968 | **-** | **-** |
| FOXK1 | 0.98 | 0.906 | 0.801 | 0.851 | **-** | **-** |
| FOXK2 | 0.97 | 0.845 | 0.546 | 0.663 | **-** | **-** |
| FOXP2 | 0.994 | 0.905 | 1.0 | 0.95 | **-** | **-** |
| GABPA | 0.976 | 0.852 | 0.788 | 0.819 | **-** | **-** |
| GATA1 | 0.965 | 0.767 | 0.519 | 0.619 | **-** | **-** |
| GATA2 | 0.978 | 0.909 | 0.759 | 0.827 | **-** | **-** |
| GATA3 | 0.995 | 0.882 | 1.000 | 0.937 | **-** | **-** |
| GATA4 | 0.971 | 0.818 | 0.667 | 0.735 | **-** | **-** |
| GATA6 | 0.976 | 0.810 | 0.818 | 0.814 | **-** | **-** |
| GRHL2 | 0.976 | 0.909 | 0.762 | 0.829 | **-** | **-** |
| HAND2 | 0.984 | 0.846 | 0.843 | 0.845 | **-** | **-** |
| HIF1A | 0.978 | 0.88 | 0.641 | 0.741 | **-** | **-** |
| HLF | 0.987 | 0.925 | 0.881 | 0.902 | **-** | **-** |
| HNF4A | 0.987 | 0.856 | 0.996 | 0.921 | **-** | **-** |
| HNF4G | 0.949 | 0.729 | 0.5 | 0.593 | **-** | **-** |
| HOXB13 | 0.969 | 0.73 | 0.891 | 0.802 | **-** | **-** |
| IKZF1 | 0.968 | 0.709 | 0.794 | 0.749 | **-** | **-** |
| IRF1 | 0.925 | 0.585 | 0.993 | 0.736 | **-** | **-** |
| JUN | 0.995 | 0.925 | 0.997 | 0.959 | **-** | **-** |
| JUNB | 0.98 | 0.745 | 0.968 | 0.842 | **-** | **-** |
| JUND | 0.985 | 0.791 | 0.993 | 0.881 | **-** | **-** |
| KLF15 | 0.949 | 0.522 | 0.93 | 0.669 | **-** | **-** |
| KLF4 | 0.969 | 0.732 | 0.694 | 0.712 | **-** | **-** |
| KLF5 | 0.953 | 0.515 | 0.985 | 0.676 | **-** | **-** |
| KLF9 | 0.925 | 0.457 | 0.84 | 0.592 | **-** | **-** |
| LHX2 | 0.977 | 0.861 | 0.702 | 0.773 | **-** | **-** |
| MAFF | 0.966 | 0.872 | 0.724 | 0.791 | **-** | **-** |
| MAFGNFE2L1 | 0.993 | 0.956 | 0.952 | 0.954 | **-** | **-** |
| MAFK | 0.954 | 0.809 | 0.507 | 0.624 | **-** | **-** |
| MAFNFE2 | 0.993 | 0.934 | 0.982 | 0.957 | **-** | **-** |
| MAX | 0.988 | 0.811 | 0.999 | 0.895 | **-** | **-** |
| MAZ | 0.961 | 0.596 | 0.901 | 0.718 | **-** | **-** |
| MEF2A | 0.991 | 0.893 | 1.0 | 0.943 | **-** | **-** |
| MEF2C | 0.988 | 0.868 | 0.995 | 0.928 | **-** | **-** |
| MEIS2 | 0.976 | 0.895 | 0.773 | 0.830 | **-** | **-** |
| MITF | 0.989 | 0.945 | 0.935 | 0.94 | **-** | **-** |
| MXI1 | 0.976 | 0.885 | 0.723 | 0.796 | **-** | **-** |
| MYB | 0.985 | 0.894 | 0.8 | 0.844 | **-** | **-** |
| MYC | 0.975 | 0.876 | 0.677 | 0.764 | **-** | **-** |
| MYCN | 0.984 | 0.859 | 0.87 | 0.864 | **-** | **-** |
| MYF5 | 0.97 | 0.732 | 0.786 | 0.758 | **-** | **-** |
| MYOD1 | 0.977 | 0.846 | 0.797 | 0.821 | **-** | **-** |
| MYOG | 0.971 | 0.715 | 0.858 | 0.78 | **-** | **-** |
| MZF1 | 0.966 | 0.748 | 0.727 | 0.737 | **-** | **-** |
| NEUROD1 | 0.966 | 0.702 | 0.837 | 0.764 | **-** | **-** |
| NEUROG2 | 0.972 | 0.832 | 0.713 | 0.767 | **-** | **-** |
| NFIB | 0.995 | 0.971 | 0.98 | 0.976 | **-** | **-** |
| NFIC | 0.969 | 0.909 | 0.483 | 0.631 | **-** | **-** |
| NFIL3 | 0.975 | 0.876 | 0.716 | 0.788 | **-** | **-** |
| NFKB1 | 0.990 | 0.846 | 0.994 | 0.914 | **-** | **-** |
| NFYA | 0.867 | 0.403 | 0.997 | 0.574 | **-** | **-** |
| NFYB | 0.912 | 0.461 | 0.997 | 0.631 | **-** | **-** |
| NFYC | 0.965 | 0.772 | 0.505 | 0.611 | **-** | **-** |
| NKX2-2 | 0.973 | 0.883 | 0.714 | 0.789 | **-** | **-** |
| NKX2-5 | 0.969 | 0.875 | 0.508 | 0.643 | **-** | **-** |
| NR1H4 | 0.972 | 0.862 | 0.584 | 0.696 | **-** | **-** |
| NR2F2 | 0.968 | 0.726 | 0.659 | 0.691 | **-** | **-** |
| NR4A1 | 0.97 | 0.691 | 0.705 | 0.698 | **-** | **-** |
| NRF1 | 0.938 | 0.467 | 0.909 | 0.617 | **-** | **-** |
| ONECUT1 | 0.972 | 0.786 | 0.898 | 0.838 | **-** | **-** |
| OSR2 | 0.966 | 0.826 | 0.551 | 0.661 | **-** | **-** |
| OTX2 | 0.973 | 0.841 | 0.682 | 0.753 | **-** | **-** |
| PAX5 | 0.994 | 0.940 | 1.000 | 0.969 | **-** | **-** |
| PBX2 | 0.977 | 0.890 | 0.695 | 0.781 | **-** | **-** |
| PBX3 | 0.982 | 0.914 | 0.867 | 0.89 | **-** | **-** |
| PHOX2B | 0.943 | 0.593 | 0.919 | 0.721 | **-** | **-** |
| PKNOX1 | 0.976 | 0.908 | 0.764 | 0.83 | **-** | **-** |
| POU2F2 | 0.992 | 0.889 | 0.998 | 0.940 | **-** | **-** |
| POU2F3 | 0.976 | 0.756 | 0.923 | 0.831 | **-** | **-** |
| POU5F1 | 0.968 | 0.806 | 0.55 | 0.654 | **-** | **-** |
| PRDM1 | 0.967 | 0.697 | 0.995 | 0.820 | **-** | **-** |
| RBPJ | 0.985 | 0.862 | 0.844 | 0.853 | **-** | **-** |
| RELB | 0.965 | 0.869 | 0.429 | 0.575 | **-** | **-** |
| RFX1 | 0.953 | 0.859 | 0.391 | 0.538 | **-** | **-** |
| RFX2 | 0.961 | 0.832 | 0.74 | 0.783 | **-** | **-** |
| RFX5 | 0.992 | 0.910 | 0.997 | 0.952 | **-** | **-** |
| RUNX2 | 0.894 | 0.414 | 0.991 | 0.584 | **-** | **-** |
| RUNX3 | 0.963 | 0.779 | 0.531 | 0.632 | **-** | **-** |
| SCRT1 | 0.989 | 0.941 | 0.915 | 0.928 | **-** | **-** |
| SCRT2 | 0.986 | 0.932 | 0.893 | 0.912 | **-** | **-** |
| SIX1 | 0.982 | 0.905 | 0.753 | 0.822 | **-** | **-** |
| SIX2 | 0.984 | 0.917 | 0.876 | 0.896 | **-** | **-** |
| SNAI2 | 0.983 | 0.868 | 0.873 | 0.87 | **-** | **-** |
| SOX10 | 0.982 | 0.858 | 0.808 | 0.832 | **-** | **-** |
| SOX13 | 0.974 | 0.827 | 0.667 | 0.738 | **-** | **-** |
| SOX2 | 0.967 | 0.805 | 0.532 | 0.64 | **-** | **-** |
| SP1 | 0.919 | 0.405 | 0.987 | 0.574 | **-** | **-** |
| SP2 | 0.940 | 0.555 | 0.973 | 0.707 | **-** | **-** |
| SPIB | 0.976 | 0.82 | 0.898 | 0.857 | **-** | **-** |
| SRF | 0.974 | 0.936 | 0.768 | 0.843 | **-** | **-** |
| STAT1 | 0.982 | 0.753 | 0.992 | 0.856 | **-** | **-** |
| STAT1STAT2 | 0.961 | 0.661 | 0.992 | 0.793 | **-** | **-** |
| STAT3 | 0.993 | 0.884 | 0.998 | 0.938 | **-** | **-** |
| TCF12 | 0.957 | 0.765 | 0.316 | 0.447 | **-** | **-** |
| TCF3 | 0.961 | 0.769 | 0.428 | 0.55 | **-** | **-** |
| TCF4 | 0.965 | 0.759 | 0.683 | 0.719 | **-** | **-** |
| TCF7 | 0.975 | 0.848 | 0.663 | 0.744 | **-** | **-** |
| TCF7L2 | 0.994 | 0.923 | 1.0 | 0.96 | **-** | **-** |
| TEAD1 | 0.983 | 0.912 | 0.821 | 0.864 | **-** | **-** |
| TEAD2 | 0.968 | 0.862 | 0.607 | 0.713 | **-** | **-** |
| TEAD4 | 0.977 | 0.869 | 0.73 | 0.794 | **-** | **-** |
| TFAP2A | 0.978 | 0.903 | 0.793 | 0.844 | **-** | **-** |
| TFAP2C | 0.956 | 0.819 | 0.526 | 0.641 | **-** | **-** |
| TFDP1 | 0.965 | 0.704 | 0.623 | 0.661 | **-** | **-** |
| TP53 | 0.976 | 0.757 | 0.994 | 0.859 | **-** | **-** |
| TP63 | 0.911 | 0.956 | 0.117 | 0.208 | **-** | **-** |
| TWIST1 | 0.979 | 0.879 | 0.784 | 0.829 | **-** | **-** |
| USF1 | 0.974 | 0.707 | 0.901 | 0.793 | **-** | **-** |
| USF2 | 0.961 | 0.813 | 0.368 | 0.506 | **-** | **-** |
| YY1 | 0.965 | 0.675 | 0.806 | 0.734 | **-** | **-** |
| YY2 | 0.972 | 0.825 | 0.613 | 0.703 | **-** | **-** |
| ZBTB12 | 0.97 | 0.815 | 0.583 | 0.68 | **-** | **-** |
| ZBTB14 | 0.958 | 0.61 | 0.816 | 0.698 | **-** | **-** |
| ZBTB26 | 0.959 | 0.707 | 0.783 | 0.743 | **-** | **-** |
| ZBTB33 | 0.98 | 0.793 | 1.0 | 0.884 | **-** | **-** |
| ZBTB6 | 0.972 | 0.776 | 0.807 | 0.791 | **-** | **-** |
| ZBTB7A | 0.972 | 0.829 | 0.719 | 0.77 | **-** | **-** |
| ZEB1 | 0.911 | 0.321 | 0.87 | 0.469 | **-** | **-** |
| ZFP42 | 0.985 | 0.948 | 0.909 | 0.928 | **-** | **-** |
| ZFP57 | 0.968 | 0.742 | 0.783 | 0.762 | **-** | **-** |
| ZIM3 | 0.982 | 0.836 | 0.949 | 0.889 | **-** | **-** |
| ZKSCAN1 | 0.988 | 0.849 | 0.916 | 0.882 | **-** | **-** |
| ZKSCAN5 | 0.953 | 0.631 | 0.805 | 0.708 | **-** | **-** |
| ZNF135 | 0.962 | 0.664 | 0.919 | 0.771 | **-** | **-** |
| ZNF136 | 0.994 | 0.957 | 0.967 | 0.962 | **-** | **-** |
| ZNF140 | 0.993 | 0.987 | 0.946 | 0.966 | **-** | **-** |
| ZNF148 | 0.919 | 0.412 | 0.815 | 0.547 | **-** | **-** |
| ZNF16 | 0.997 | 0.988 | 0.982 | 0.985 | **-** | **-** |
| ZNF189 | 0.972 | 0.785 | 0.781 | 0.783 | **-** | **-** |
| ZNF24 | 0.881 | 0.345 | 0.917 | 0.501 | **-** | **-** |
| ZNF263 | 0.79 | 0.27 | 0.588 | 0.37 | **-** | **-** |
| ZNF317 | 0.973 | 0.758 | 0.806 | 0.781 | **-** | **-** |
| ZNF331 | 0.972 | 0.808 | 0.793 | 0.801 | **-** | **-** |
| ZNF341 | 0.971 | 0.847 | 0.621 | 0.716 | **-** | **-** |
| ZNF382 | 0.983 | 0.903 | 0.961 | 0.931 | **-** | **-** |
| ZNF384 | 0.921 | 0.425 | 0.918 | 0.581 | **-** | **-** |
| ZNF449 | 0.98 | 0.857 | 0.855 | 0.856 | **-** | **-** |
| ZNF460 | 0.942 | 0.593 | 0.887 | 0.711 | **-** | **-** |
| ZNF528 | 0.995 | 0.968 | 0.976 | 0.972 | **-** | **-** |
| ZNF549 | 0.973 | 0.835 | 0.676 | 0.747 | **-** | **-** |
| ZNF610 | 0.925 | 0.481 | 0.941 | 0.637 | **-** | **-** |
| ZNF652 | 0.981 | 0.906 | 0.763 | 0.828 | **-** | **-** |
| ZNF680 | 0.985 | 0.884 | 0.922 | 0.903 | **-** | **-** |
| ZNF682 | 0.995 | 0.959 | 0.986 | 0.972 | **-** | **-** |
| ZNF707 | 0.993 | 0.953 | 0.957 | 0.955 | **-** | **-** |
| ZNF708 | 0.99 | 0.939 | 0.925 | 0.932 | **-** | **-** |
| ZNF8 | 0.994 | 0.978 | 0.965 | 0.971 | **-** | **-** |
| ZNF816 | 0.975 | 0.82 | 0.931 | 0.872 | **-** | **-** |
| ZNF85 | 0.996 | 0.959 | 0.991 | 0.975 | **-** | **-** |
| ZNF93 | 0.951 | 0.627 | 0.955 | 0.757 | **-** | **-** |

**Supplementary Table S8. Performance comparison of multi-task learning and single-task learning on 188 TFs datasets**

| Model | Acc | Pre | Rec | F1-S | AUC | AUPR |
| --- | --- | --- | --- | --- | --- | --- |
| BertSNR-single | 0.984 | 0.844 | 0.944 | 0.890 | 0.994 | 0.946 |
| BertSNR-multi | 0.985 | 0.852 | 0.942 | 0.892 | 0.995 | 0.949 |

**Supplementary Table S9. Performance comparison of different k-mer pre-training models**

| Model | Acc | Pre | Rec | F1-S | AUC | AUPR |
| --- | --- | --- | --- | --- | --- | --- |
| 3-mer | 0.985±0.011 | 0.854±0.073 | 0.940±0.090 | 0.893±0.075 | 0.994±0.010 | 0.949±0.058 |
| 4-mer | 0.985±0.010 | 0.852±0.069 | 0.944±0.091 | 0.893±0.076 | 0.993±0.009 | 0.945±0.061 |
| 5-mer | 0.985±0.012 | 0.849±0.099 | 0.936±0.113 | 0.887±0.101 | 0.991±0.029 | 0.940±0.088 |
| 6-mer | 0.985±0.011 | 0.846±0.097 | 0.945±0.110 | 0.891±0.100 | 0.991±0.030 | 0.944±0.087 |

**Supplementary Table S10. Motif logos comparison of various models.**

| TFs | BertSNR | D-AEDNet | DeepSNR |
| --- | --- | --- | --- |
| BATFJUN | 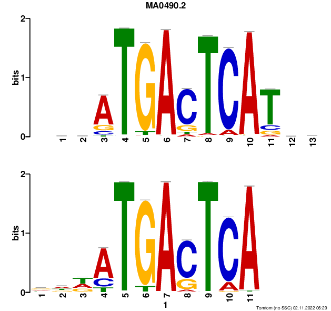 | 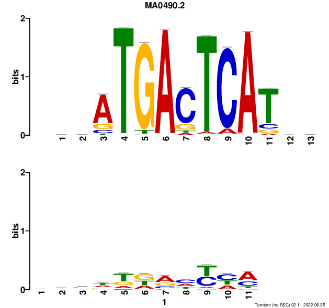 | 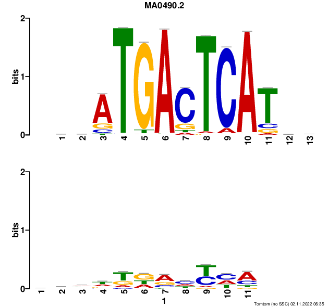 |
| CDX2 | 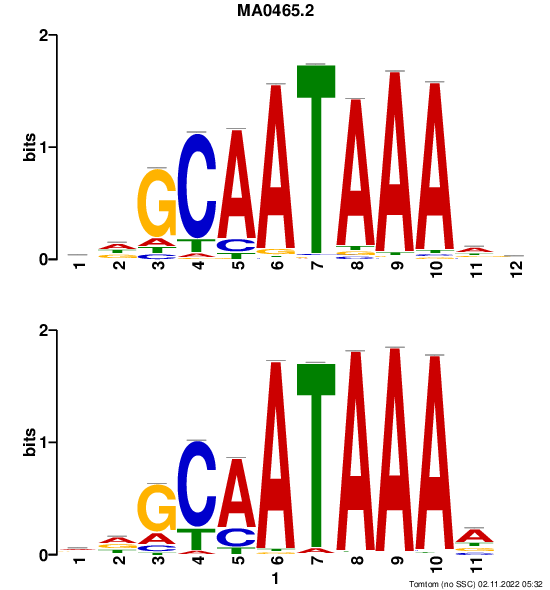 | 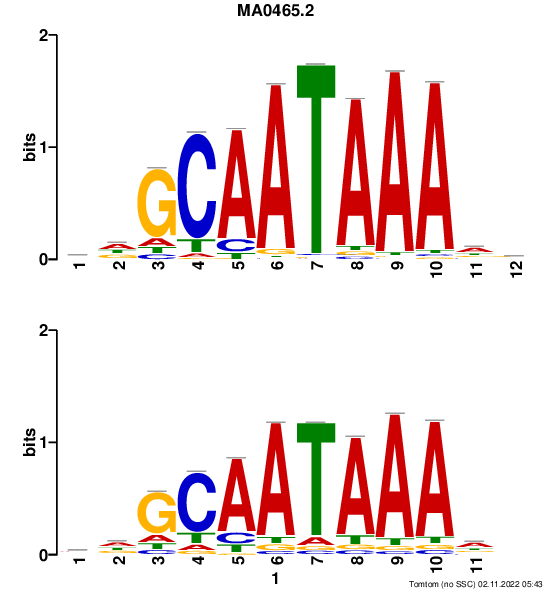 | 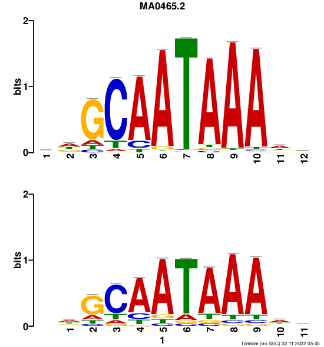 |
| CEBPA | 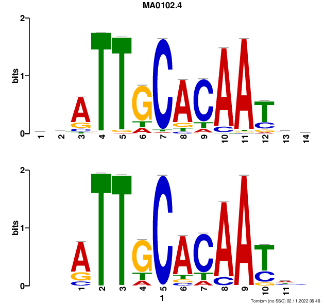 | 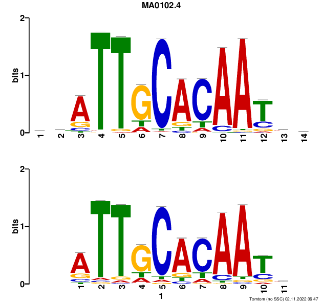 | 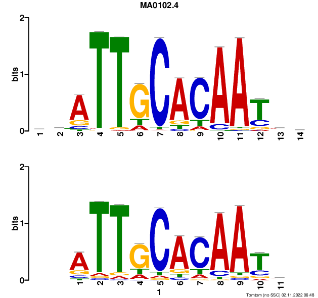 |
| CTCFL | 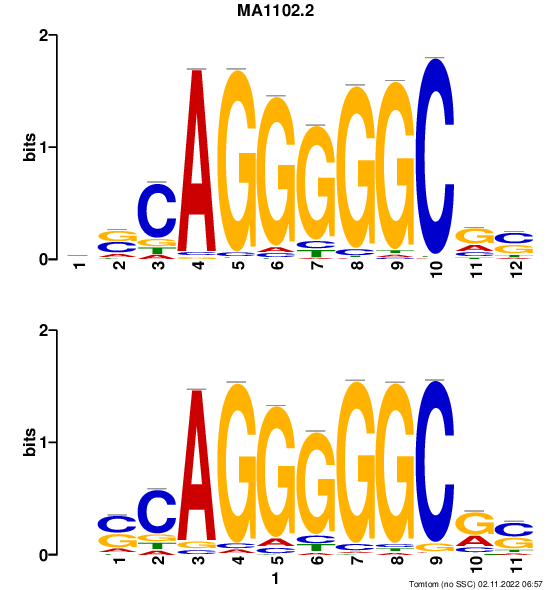 | 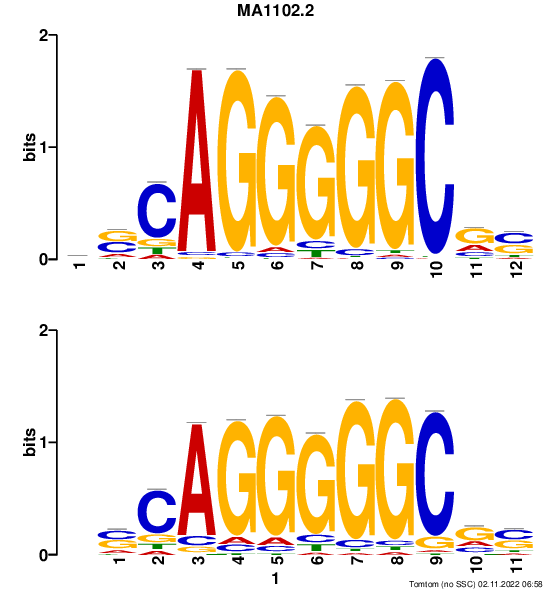 | 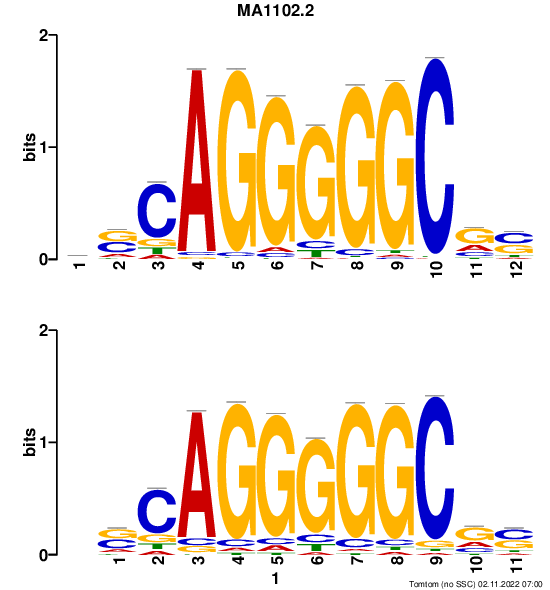 |
| E2F6 | 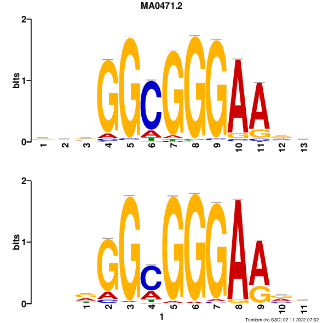 | 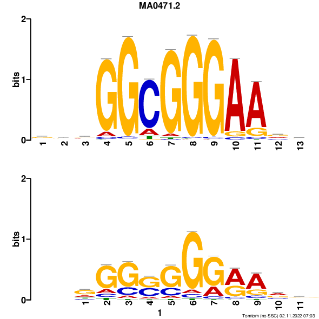 | 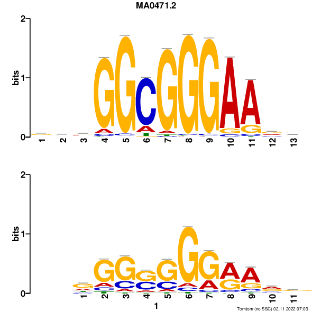 |
| EBF1 | 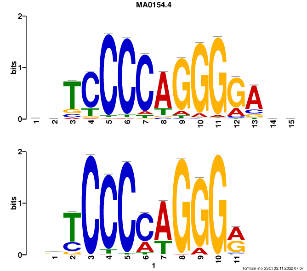 | 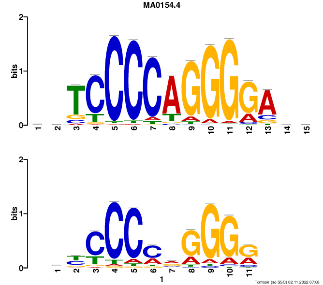 | 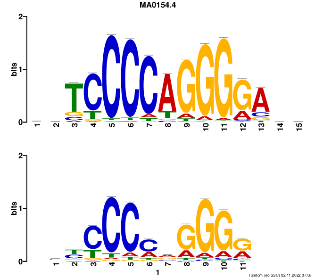 |
| EGR1 | 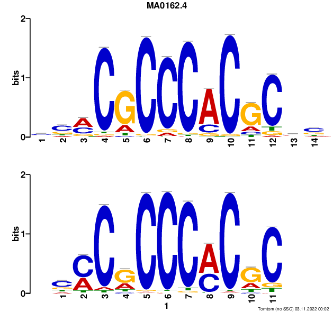 | 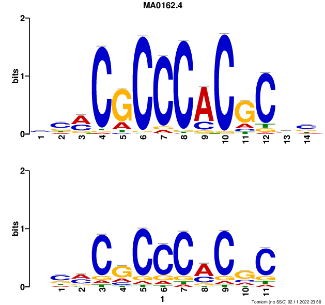 | 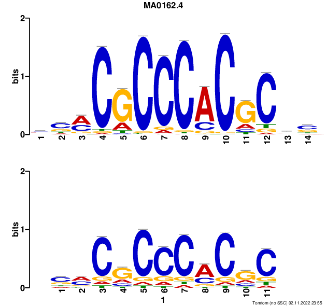 |
| ELF1 | 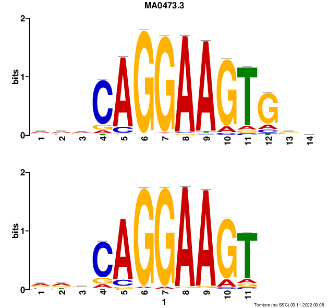 | 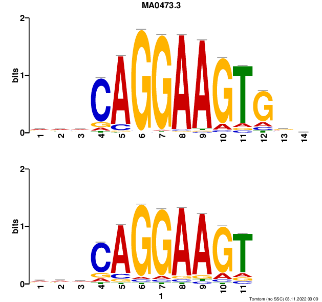 | 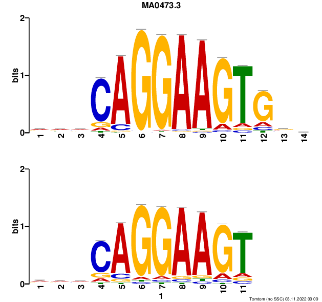 |
| FOSL1 | 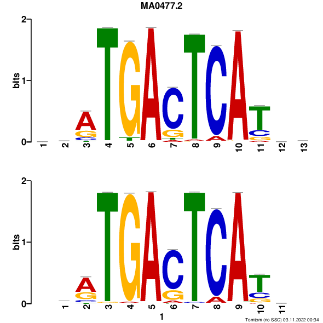 | 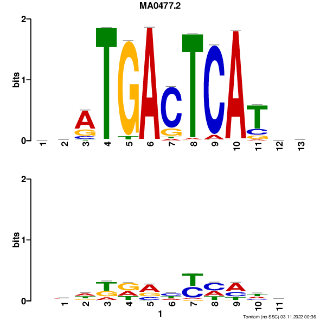 | 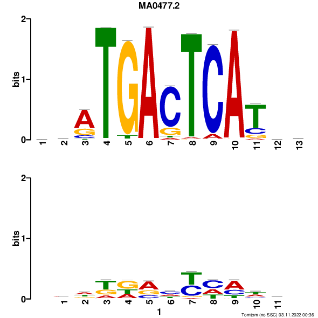 |
| FOXA1 | 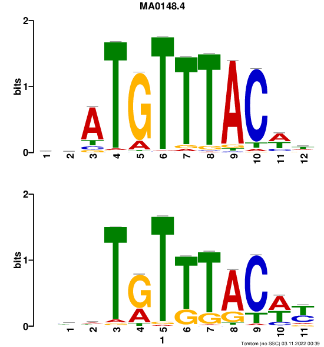 | - | - |
| FOXK2 | 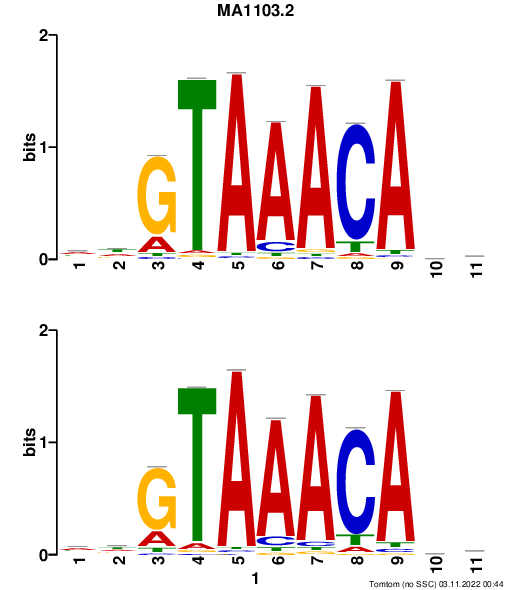 | 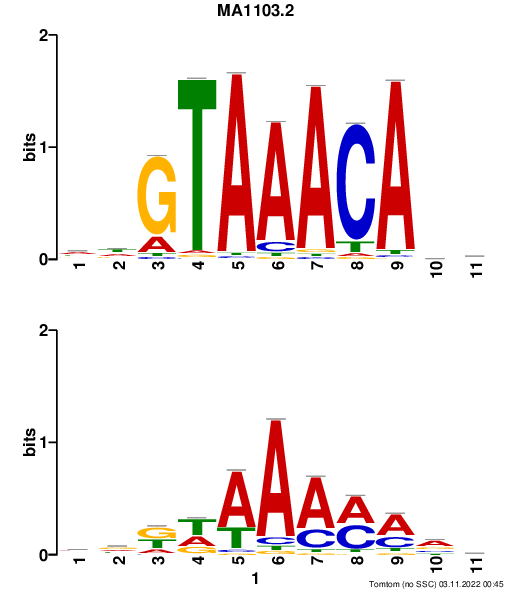 | 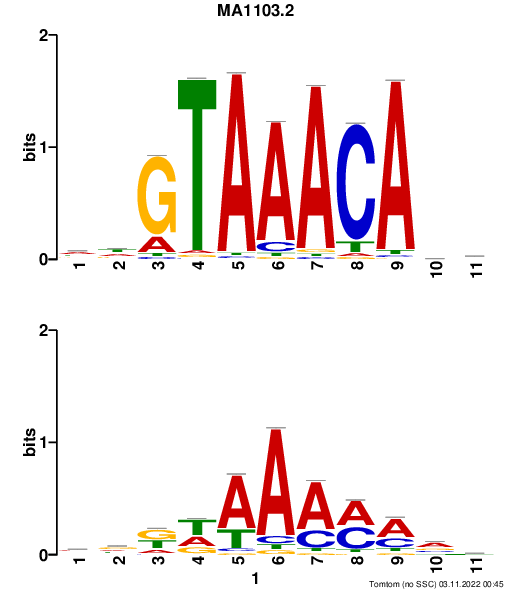 |
| GATA2 | 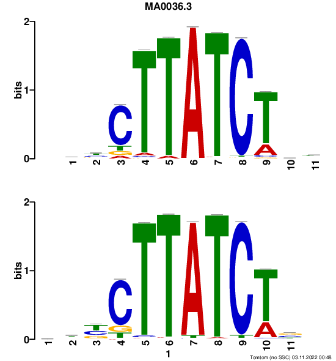 | 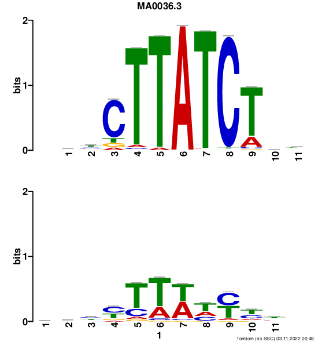 | 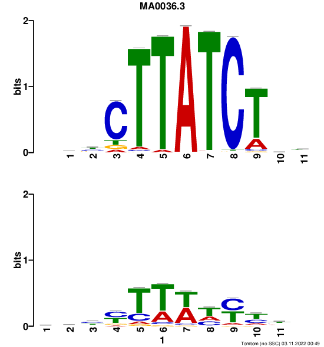 |
| GATA6 | 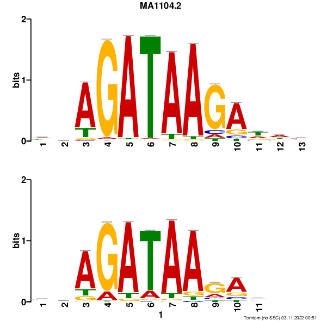 | - | - |
| GRHL2 | 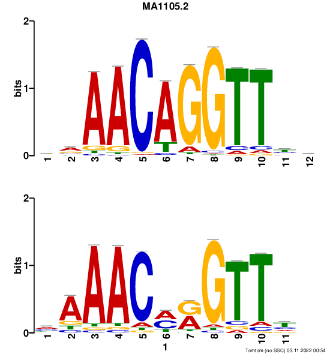 | 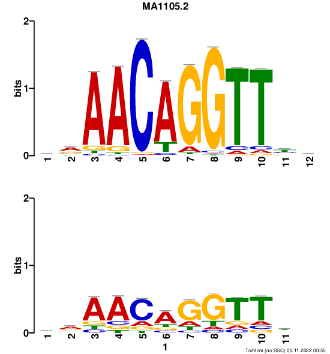 | 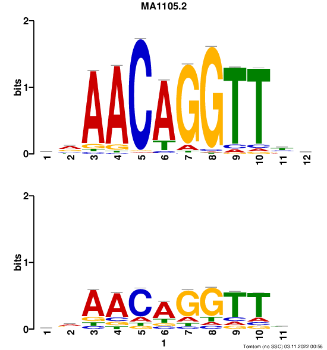 |
| HNF4A | 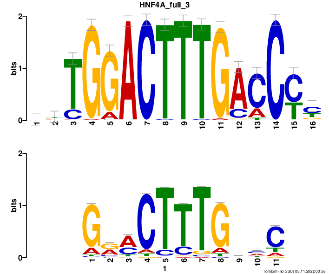 | - | - |
| HNF4G | 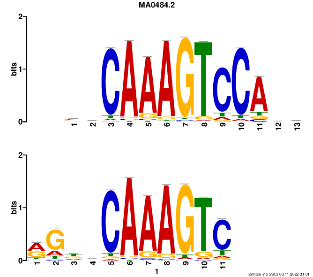 | 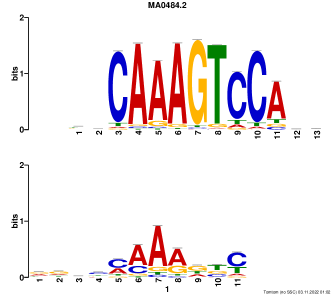 | 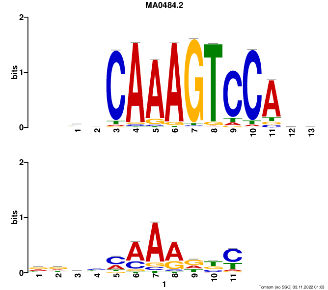 |
| JUNB | 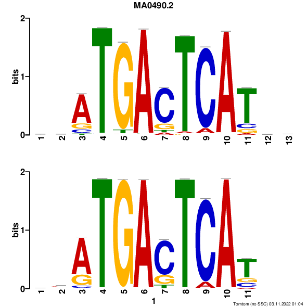 | 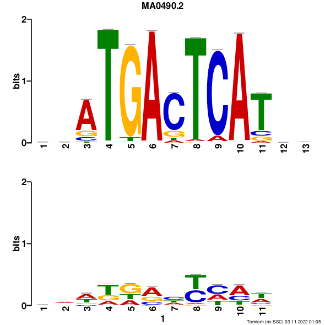 | 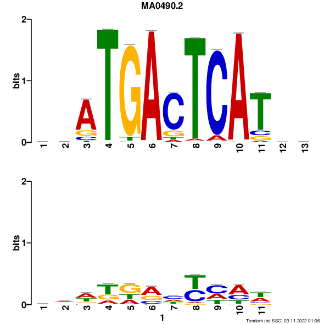 |
| JUND | 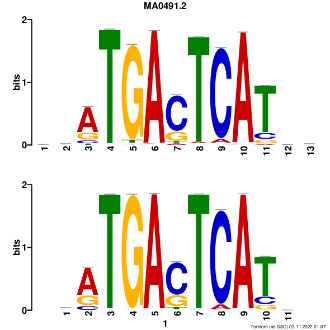 | 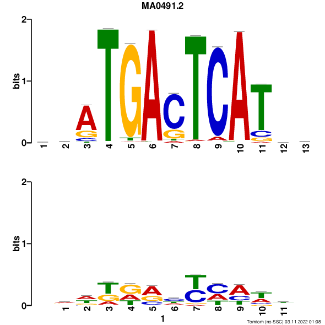 | 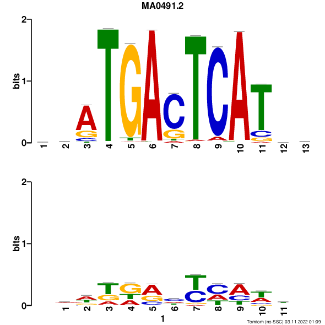 |
| KLF4 | 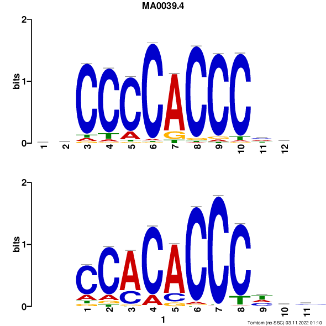 | 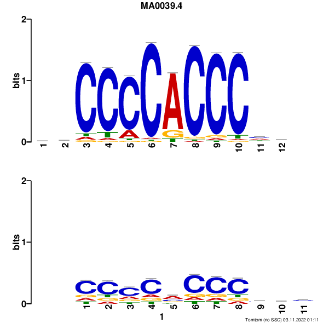 | 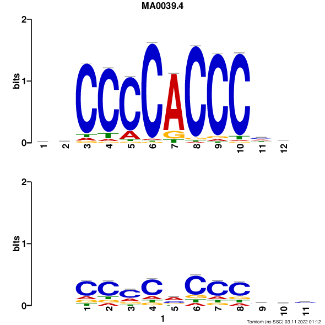 |
| KLF9 | 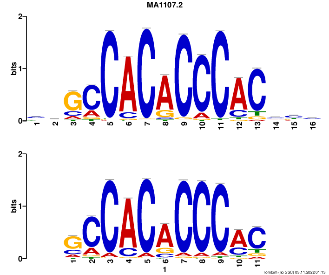 | 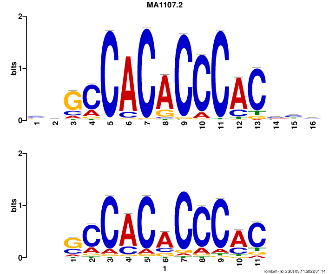 | 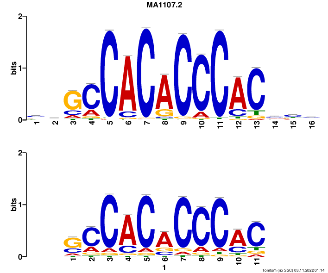 |
| MAFF | 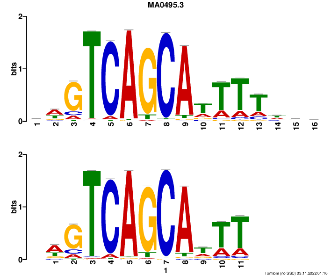 | 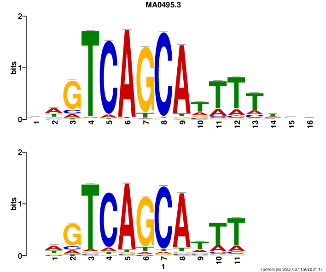 | 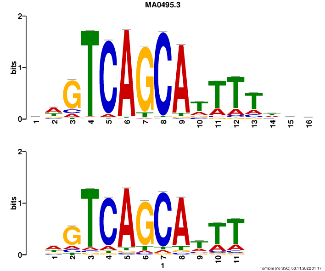 |
| MAFK | 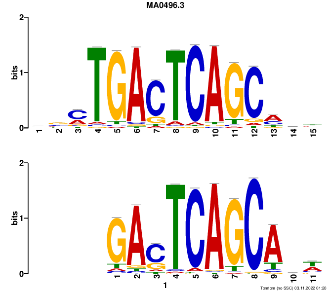 | 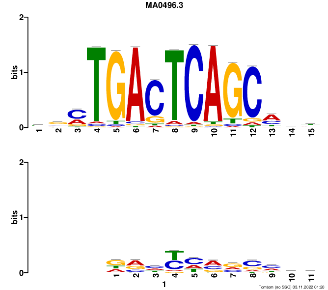 | 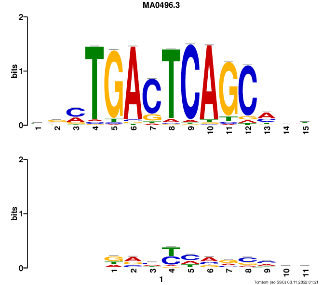 |
| MEF2A | 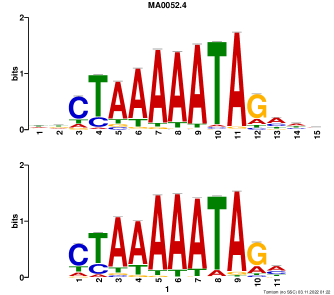 | 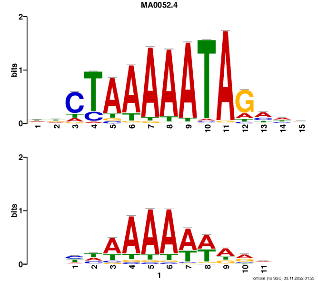 | 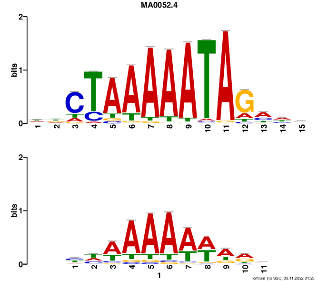 |
| MITF | 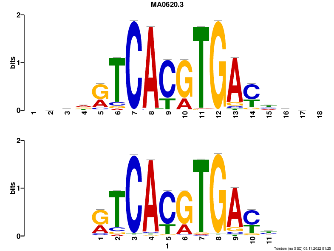 | 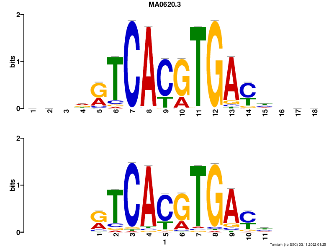 | 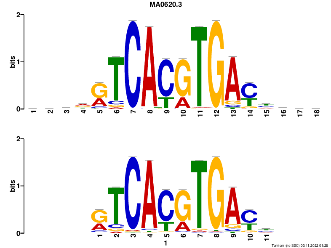 |
| MXI1 | 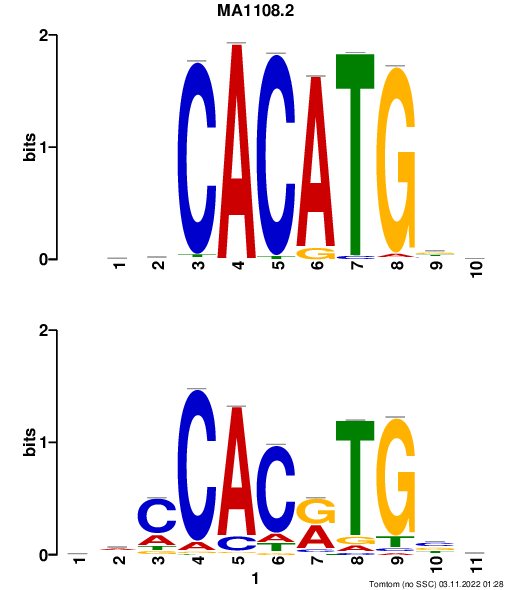 | 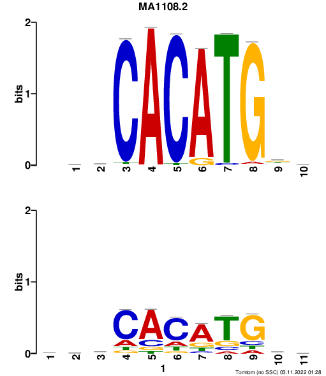 | 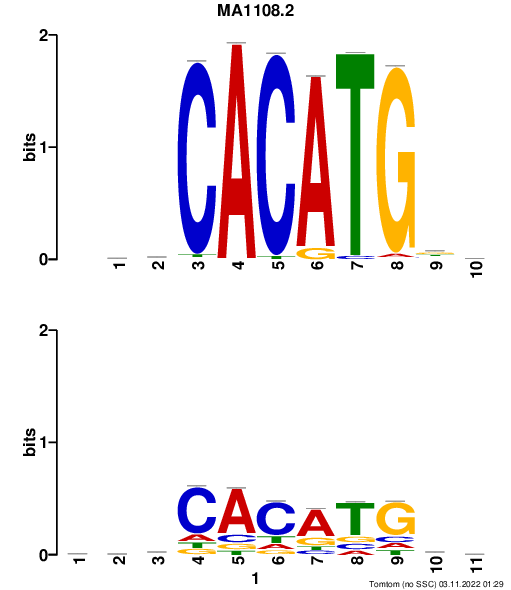 |
| NFYA | 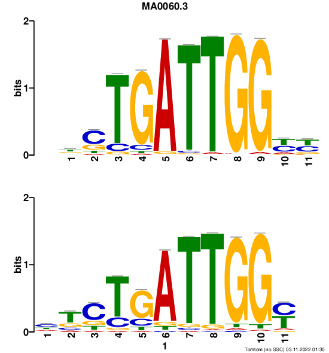 | - | - |
| NFYB | 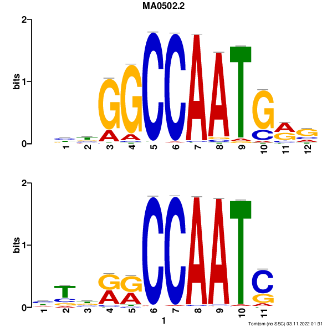 | - | - |
| NR4A1 | 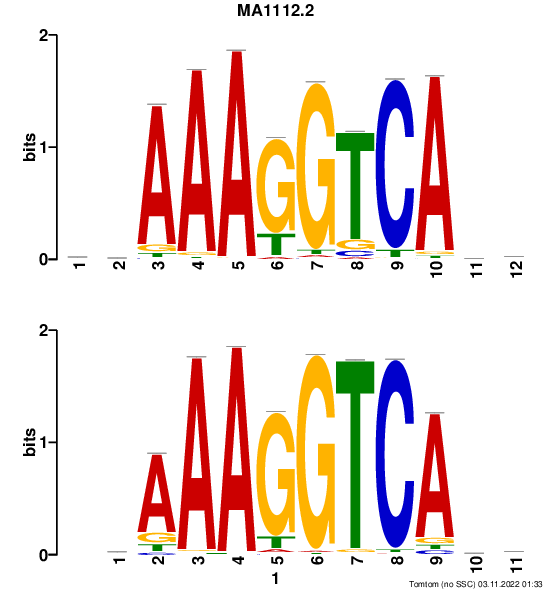 | 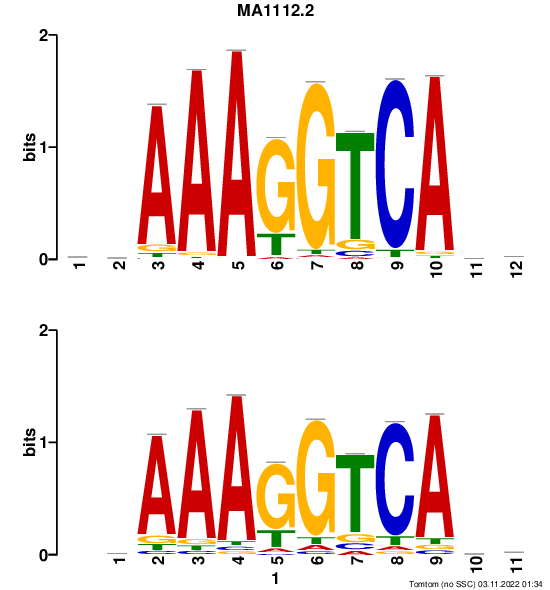 | 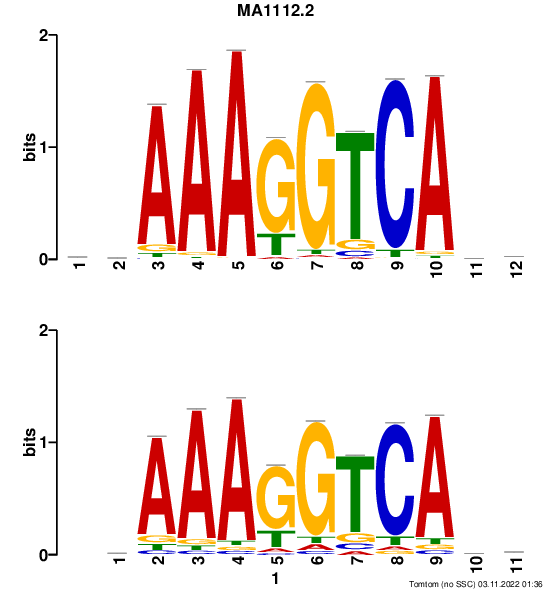 |
| PRDM1 | 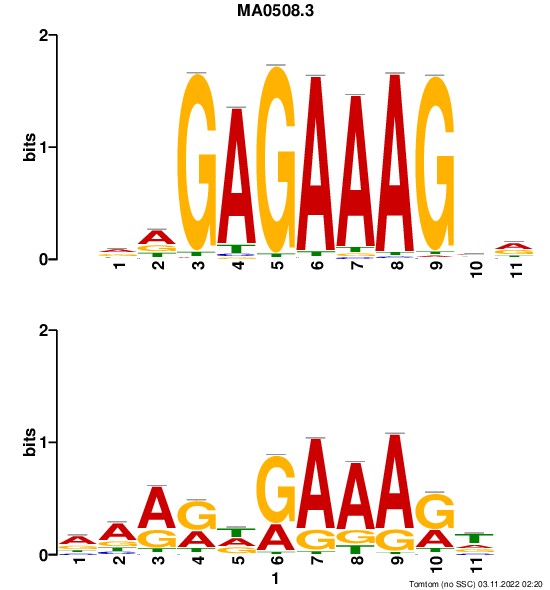 | - | - |
| TFAP2A | 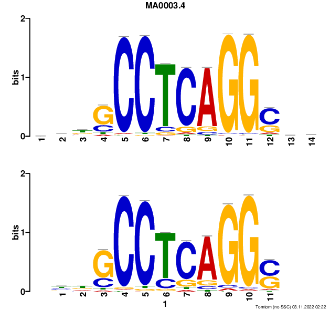 | 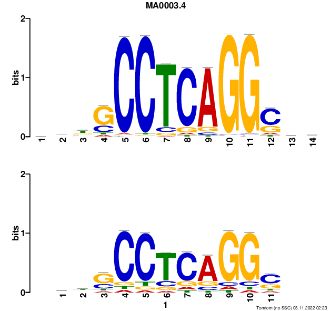 | 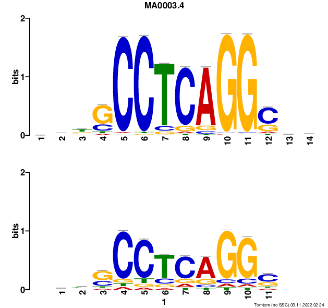 |
| USF1 | 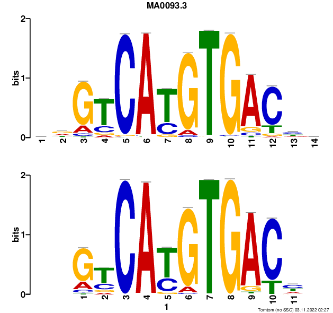 | 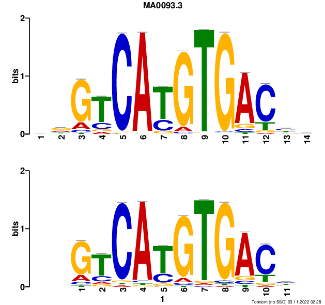 | 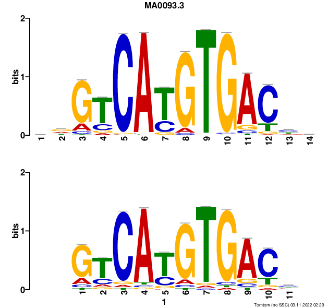 |
| USF2 | 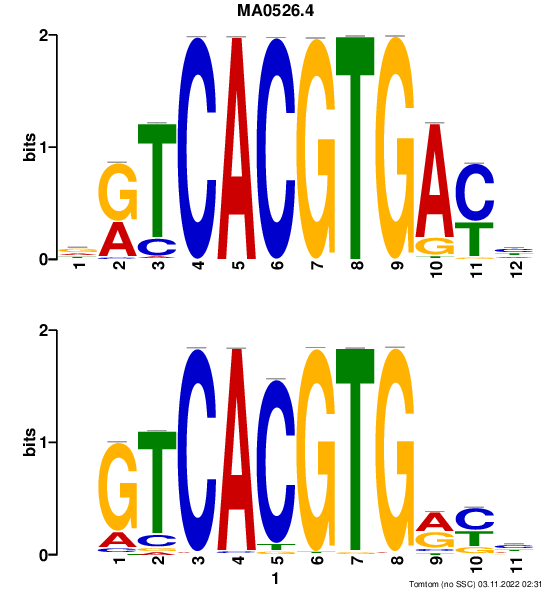 | 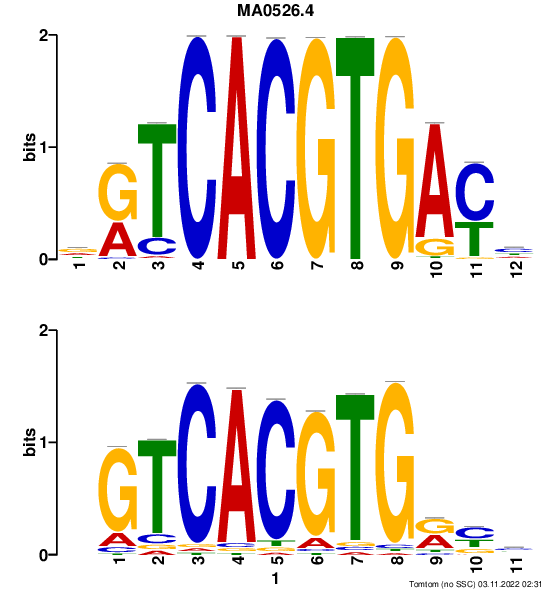 | 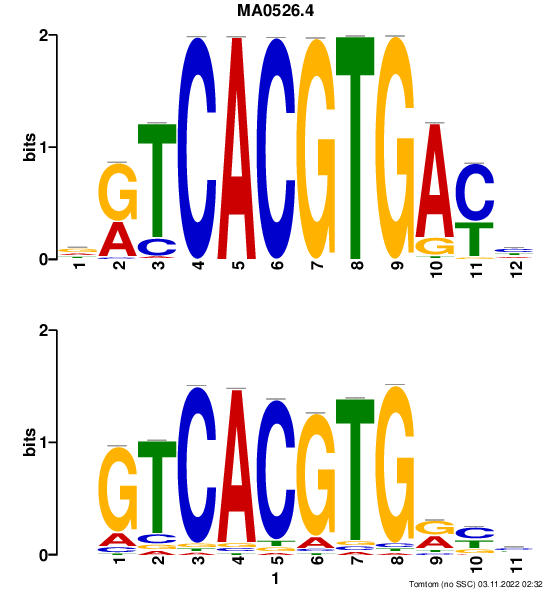 |
| ZEB1 | 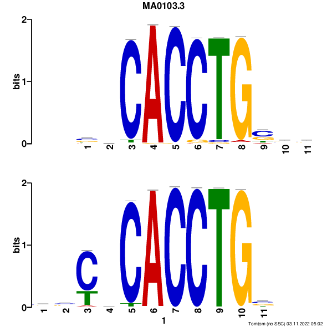 | 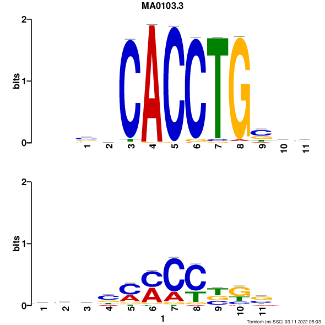 | 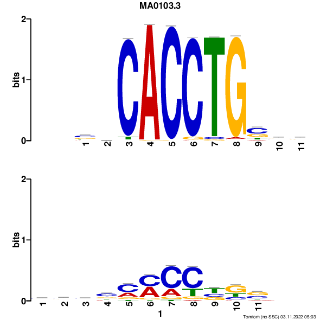 |

**Supplementary Table S11. The detailed p-value, e-value and q-value of various models.**

| Dataset | Model | p-value | e-value | q-value |
| --- | --- | --- | --- | --- |
| BATFJUN | DeepSNR | 1.02E-04 | 2.12E-01 | 2.74E-01 |
|  | D-AEDNet | 1.69E-04 | 3.50E-01 | 3.88E-01 |
|  | BertSNR | 2.09E-06 | 4.32E-03 | 2.85E-03 |
| CDX2 | DeepSNR | 3.24E-12 | 6.71E-09 | 1.31E-08 |
|  | D-AEDNet | 8.84E-12 | 1.83E-08 | 3.56E-08 |
|  | BertSNR | 6.54E-13 | 1.35E-09 | 2.63E-09 |
| CEBPA | DeepSNR | 1.60E-10 | 3.30E-07 | 6.57E-07 |
|  | D-AEDNet | 4.55E-12 | 9.41E-09 | 1.87E-08 |
|  | BertSNR | 1.04E-13 | 2.14E-10 | 4.26E-10 |
| CTCFL | DeepSNR | 2.92E-16 | 6.05E-13 | 1.18E-12 |
|  | D-AEDNet | 1.57E-15 | 3.25E-12 | 6.34E-12 |
|  | BertSNR | 2.17E-14 | 4.50E-11 | 8.76E-11 |
| E2F6 | DeepSNR | 8.45E-08 | 1.75E-04 | 3.48E-04 |
|  | D-AEDNet | 2.50E-07 | 5.18E-04 | 1.03E-03 |
|  | BertSNR | 3.22E-12 | 6.66E-09 | 1.33E-08 |
| EBF1 | DeepSNR | 6.77E-06 | 1.40E-02 | 1.45E-02 |
|  | D-AEDNet | 6.46E-06 | 1.34E-02 | 1.72E-02 |
|  | BertSNR | 3.76E-07 | 7.78E-04 | 7.20E-04 |
| EGR1 | DeepSNR | 1.10E-12 | 2.29E-09 | 4.52E-09 |
|  | D-AEDNet | 3.16E-12 | 6.54E-09 | 1.29E-08 |
|  | BertSNR | 2.50E-09 | 5.17E-06 | 1.02E-05 |
| ELF1 | DeepSNR | 6.54E-14 | 1.35E-10 | 2.66E-10 |
|  | D-AEDNet | 2.01E-13 | 4.15E-10 | 8.14E-10 |
|  | BertSNR | 1.37E-16 | 2.85E-13 | 5.58E-13 |
| FOSL1 | DeepSNR | 5.53E-05 | 1.15E-01 | 6.69E-02 |
|  | D-AEDNet | 6.04E-05 | 1.25E-01 | 5.73E-02 |
|  | BertSNR | 2.60E-12 | 5.38E-09 | 1.06E-08 |
| FOXA1 | DeepSNR | - | - | - |
|  | D-AEDNet | - | - | - |
|  | BertSNR | 2.02E-08 | 4.18E-05 | 8.23E-05 |
| FOXK2 | DeepSNR | 6.18E-07 | 1.28E-03 | 1.27E-03 |
|  | D-AEDNet | 2.98E-07 | 6.18E-04 | 6.13E-04 |
|  | BertSNR | 8.05E-15 | 1.67E-11 | 3.28E-11 |
| GATA2 | DeepSNR | 4.40E-04 | 9.10E-01 | 4.98E-01 |
|  | D-AEDNet | 5.17E-04 | 1.07E+00 | 4.75E-01 |
|  | BertSNR | 1.89E-06 | 3.92E-03 | 2.58E-03 |
| GATA6 | DeepSNR | - | - | - |
|  | D-AEDNet | - | - | - |
|  | BertSNR | 4.64E-09 | 9.60E-06 | 9.60E-06 |
| GRHL2 | DeepSNR | 1.16E-09 | 2.41E-06 | 4.80E-06 |
|  | D-AEDNet | 1.55E-08 | 3.21E-05 | 6.41E-05 |
|  | BertSNR | 3.74E-07 | 7.74E-04 | 9.48E-04 |
| HNF4A | DeepSNR | 1 | 1 | 1 |
|  | D-AEDNet | 1 | 1 | 1 |
|  | BertSNR | 3.74E-07 | 7.74E-04 | 9.48E-04 |
| HNF4G | DeepSNR | 1.04E-04 | 2.15E-01 | 4.68E-02 |
|  | D-AEDNet | 5.92E-05 | 1.23E-01 | 2.68E-02 |
|  | BertSNR | 2.64E-05 | 5.46E-02 | 1.72E-02 |
| JUNB | DeepSNR | 3.77E-06 | 7.81E-03 | 1.49E-02 |
|  | D-AEDNet | 4.92E-06 | 1.02E-02 | 1.95E-02 |
|  | BertSNR | 2.23E-10 | 4.61E-07 | 3.03E-07 |
| JUND | DeepSNR | 7.08E-05 | 1.47E-01 | 4.38E-02 |
|  | D-AEDNet | 1.24E-04 | 2.58E-01 | 5.30E-02 |
|  | BertSNR | 1.33E-13 | 2.75E-10 | 2.71E-10 |
| KLF4 | DeepSNR | 9.72E-07 | 2.01E-03 | 1.99E-03 |
|  | D-AEDNet | 1.26E-06 | 2.62E-03 | 2.59E-03 |
|  | BertSNR | 1.55E-05 | 3.21E-02 | 2.11E-02 |
| KLF9 | DeepSNR | 6.63E-14 | 1.37E-10 | 2.72E-10 |
|  | D-AEDNet | 5.40E-13 | 1.12E-09 | 2.21E-09 |
|  | BertSNR | 2.91E-11 | 6.02E-08 | 1.19E-07 |
| MAFF | DeepSNR | 2.07E-13 | 4.28E-10 | 8.52E-10 |
|  | D-AEDNet | 1.91E-12 | 3.95E-09 | 7.87E-09 |
|  | BertSNR | 3.92E-11 | 8.12E-08 | 1.62E-07 |
| MAFK | DeepSNR | 5.51E-07 | 1.14E-03 | 2.28E-03 |
|  | D-AEDNet | 6.88E-07 | 1.42E-03 | 2.85E-03 |
|  | BertSNR | 8.38E-09 | 1.73E-05 | 1.15E-05 |
| MEF2A | DeepSNR | 3.24E-05 | 6.70E-02 | 4.46E-02 |
|  | D-AEDNet | 2.98E-05 | 6.16E-02 | 4.11E-02 |
|  | BertSNR | 9.02E-12 | 1.87E-08 | 3.73E-08 |
| MITF | DeepSNR | 8.21E-18 | 1.70E-14 | 1.62E-14 |
|  | D-AEDNet | 1.21E-15 | 2.51E-12 | 2.40E-12 |
|  | BertSNR | 1.11E-16 | 2.30E-13 | 2.19E-13 |
| MXI1 | DeepSNR | 1.76E-06 | 3.64E-03 | 7.09E-03 |
|  | D-AEDNet | 6.72E-07 | 1.39E-03 | 2.71E-03 |
|  | BertSNR | 4.34E-04 | 8.98E-01 | 1.24E-01 |
| NFYA | DeepSNR | - | - | - |
|  | D-AEDNet | - | - | - |
|  | BertSNR | 4.30E-07 | 8.89E-04 | 8.78E-04 |
| NFYB | DeepSNR | - | - | - |
|  | D-AEDNet | - | - | - |
|  | BertSNR | 5.49E-05 | 1.14E-01 | 1.73E-01 |
| NR4A1 | DeepSNR | 1.45E-12 | 3.00E-09 | 5.91E-09 |
|  | D-AEDNet | 2.75E-11 | 5.70E-08 | 1.12E-07 |
|  | BertSNR | 6.86E-07 | 1.42E-03 | 2.49E-03 |
| PRDM1 | DeepSNR | - | - | - |
|  | D-AEDNet | - | - | - |
|  | BertSNR | 5.90E-05 | 1.22E-01 | 6.64E-02 |
| TFAP2A | DeepSNR | 1.29E-13 | 2.67E-10 | 5.28E-10 |
|  | D-AEDNet | 3.70E-14 | 7.67E-11 | 1.51E-10 |
|  | BertSNR | 2.16E-11 | 4.48E-08 | 8.84E-08 |
| USF1 | DeepSNR | 3.48E-12 | 7.21E-09 | 1.40E-08 |
|  | D-AEDNet | 1.97E-13 | 4.08E-10 | 7.94E-10 |
|  | BertSNR | 1.16E-12 | 2.40E-09 | 4.65E-09 |
| USF2 | DeepSNR | 2.31E-08 | 4.79E-05 | 5.42E-05 |
|  | D-AEDNet | 3.22E-08 | 6.66E-05 | 4.74E-05 |
|  | BertSNR | 3.15E-09 | 6.52E-06 | 9.26E-06 |
| ZEB1 | DeepSNR | 1.03E-04 | 2.14E-01 | 5.26E-02 |
|  | D-AEDNet | 1.65E-04 | 3.42E-01 | 6.14E-02 |
|  | BertSNR | 1.80E-03 | 3.73E+00 | 7.10E-01 |

**Supplementary Table S12. The TFBSs identified by BertSNR in the POU5F1 promoter region.**

| Chromosome | Start | End | Name |
| --- | --- | --- | --- |
| chr6 | 31185494 | 31185507 | ASCL1 |
| chr6 | 31186476 | 31186489 | ASCL1 |
| chr6 | 31186966 | 31186979 | ATF2 |
| chr6 | 31186914 | 31186925 | BATF |
| chr6 | 31185496 | 31185506 | BHLHE22 |
| chr6 | 31187008 | 31187019 | DUX4 |
| chr6 | 31185185 | 31185199 | EGR1 |
| chr6 | 31186279 | 31186292 | ELF1 |
| chr6 | 31186051 | 31186065 | ELF3 |
| chr6 | 31186261 | 31186275 | ELF3 |
| chr6 | 31186686 | 31186700 | ELF3 |
| chr6 | 31186263 | 31186274 | ELK4 |
| chr6 | 31185658 | 31185673 | ESR2 |
| chr6 | 31186913 | 31186924 | FOSL2 |
| chr6 | 31186793 | 31186804 | FOXA3 |
| chr6 | 31186793 | 31186804 | FOXK2 |
| chr6 | 31186262 | 31186276 | GABPA |
| chr6 | 31185672 | 31185683 | GATA1 |
| chr6 | 31186013 | 31186024 | GATA1 |
| chr6 | 31185491 | 31185506 | GRHL2 |
| chr6 | 31185790 | 31185800 | HIF1A |
| chr6 | 31186104 | 31186114 | HIF1A |
| chr6 | 31186180 | 31186195 | HNF4G |
| chr6 | 31186707 | 31186719 | IKZF1 |
| chr6 | 31186574 | 31186589 | MEF2C |
| chr6 | 31186460 | 31186475 | MEIS2 |
| chr6 | 31185576 | 31185589 | MYOD1 |
| chr6 | 31187055 | 31187068 | MYOD1 |
| chr6 | 31185495 | 31185507 | MYOG |
| chr6 | 31185396 | 31185409 | NEUROD1 |
| chr6 | 31186476 | 31186489 | NEUROG2 |
| chr6 | 31186653 | 31186674 | NFIB |
| chr6 | 31185448 | 31185461 | NFIL3 |
| chr6 | 31185661 | 31185673 | OSR2 |
| chr6 | 31186270 | 31186289 | PAX5 |
| chr6 | 31185460 | 31185479 | RFX2 |
| chr6 | 31185657 | 31185676 | RFX2 |
| chr6 | 31187055 | 31187068 | SNAI2 |
| chr6 | 31186562 | 31186573 | SOX13 |
| chr6 | 31186561 | 31186572 | SOX2 |
| chr6 | 31186686 | 31186702 | SPIB |
| chr6 | 31186682 | 31186697 | STAT1STAT2 |
| chr6 | 31185692 | 31185703 | STAT3 |
| chr6 | 31186352 | 31186363 | TCF12 |
| chr6 | 31187056 | 31187067 | TCF12 |
| chr6 | 31187055 | 31187068 | TCF4 |
| chr6 | 31185494 | 31185507 | TWIST1 |
| chr6 | 31186997 | 31187009 | YY1 |
| chr6 | 31186568 | 31186579 | ZBTB12 |
| chr6 | 31187100 | 31187111 | ZBTB12 |
| chr6 | 31186964 | 31186977 | ZBTB6 |
| chr6 | 31187099 | 31187112 | ZBTB6 |
| chr6 | 31187088 | 31187109 | ZNF140 |
| chr6 | 31185599 | 31185613 | ZNF449 |
| chr6 | 31186106 | 31186118 | ZNF549 |
| chr6 | 31186707 | 31186719 | ZNF652 |
| chr6 | 31186280 | 31186295 | ZNF680 |
| chr6 | 31186295 | 31186311 | ZNF682 |
